# Supplementary material for: CEP192 localises mitotic Aurora-A activity by priming its interaction with TPX2
Source: EMBO J. 2024 Sep 26;43(22):5381–420. doi: 10.1038/s44318-024-00240-z (PMC11574021; doi:10.1038/s44318-024-00240-z)
Supplement: Supplementary file 1 — Appendix [file 44318_2024_240_MOESM1_ESM.pdf]

<u>**Appendix Data For:**</u>

CEP192 localises mitotic Aurora-A activity by priming its interaction with TPX2

Contents:

Appendix Figure S1: CEP192 interacts with Aurora-A kinase domain.

Appendix Figure S2: CEP192 assignment and secondary structure predictions

Appendix Figure S3: Mutating Aurora-A and inclusion of a monobody does not affect binding to CEP192

Appendix Figure S4: Testing mutants of Aurora-A and CEP192 in direct binding and competition assays

Appendix Figure S5: Comparison of DFG motif in models

Appendix Figure S6: Characterising the loss of *CEP192(exon11)* in cells

Appendix Figure S7: Binding of Aurora-A to CEP192 is essential for autophosphorylation in U251 cells

Appendix Figure S8: The activity and localisation of PLK1 is unchanged during mitosis in the absence of Aurora-A:CEP192 complexes

Appendix Figure S9: MAFFT alignment of Aurora-A binding region of CEP192 orthologues.

Appendix Figure S10: Comparison of the co-crystal structure with CEP192 fused to Aurora-A

Appendix Table S1: Data collection and refinement statistics

Appendix Table S2: Summary of the KinCoRe analysis of Aurora-A bound to CEP192, in comparison to published structures of Aurora-A.

Appendix Table S3: Summary of biophysical assays

Appendix Table S4: Primers used within this study

Appendix References

Fig S1.

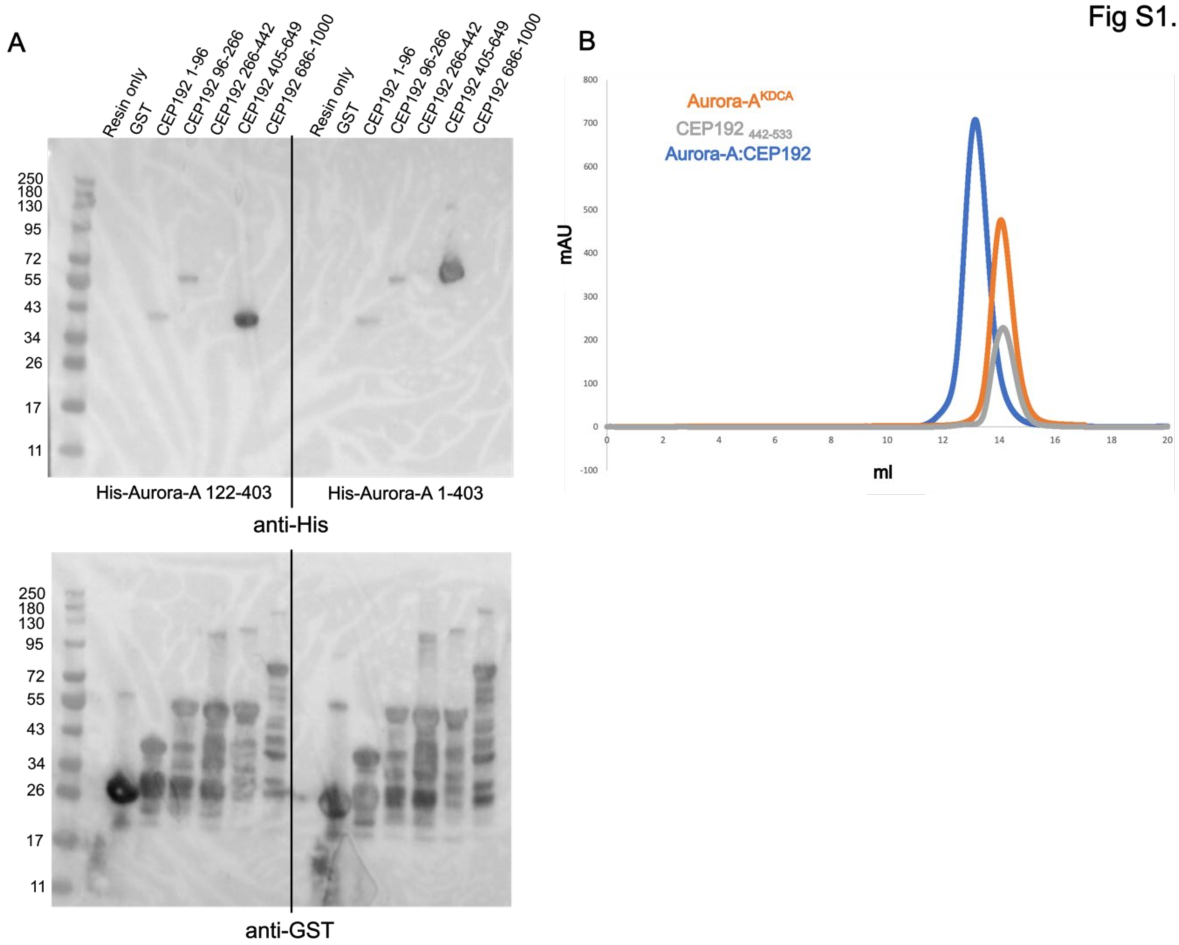

Appendix Figure S1: CEP192 interacts with Aurora-A kinase domain.
A Western blots to show the co-precipitation of histidine tagged Aurora-A kinase domain (122-403) and full-length (1-403) by fragments of CEP192 expressed with a GST tag at the N-terminus. Only GST-tagged CEP192 405-649 was able to interact significantly with Aurora-A.

B Analytical SEC of Aurora-A 122-403 in complex with CEP192 442-533. A clear complex is seen as the elution volume is significantly earlier than either of the proteins alone.

Fig. S2

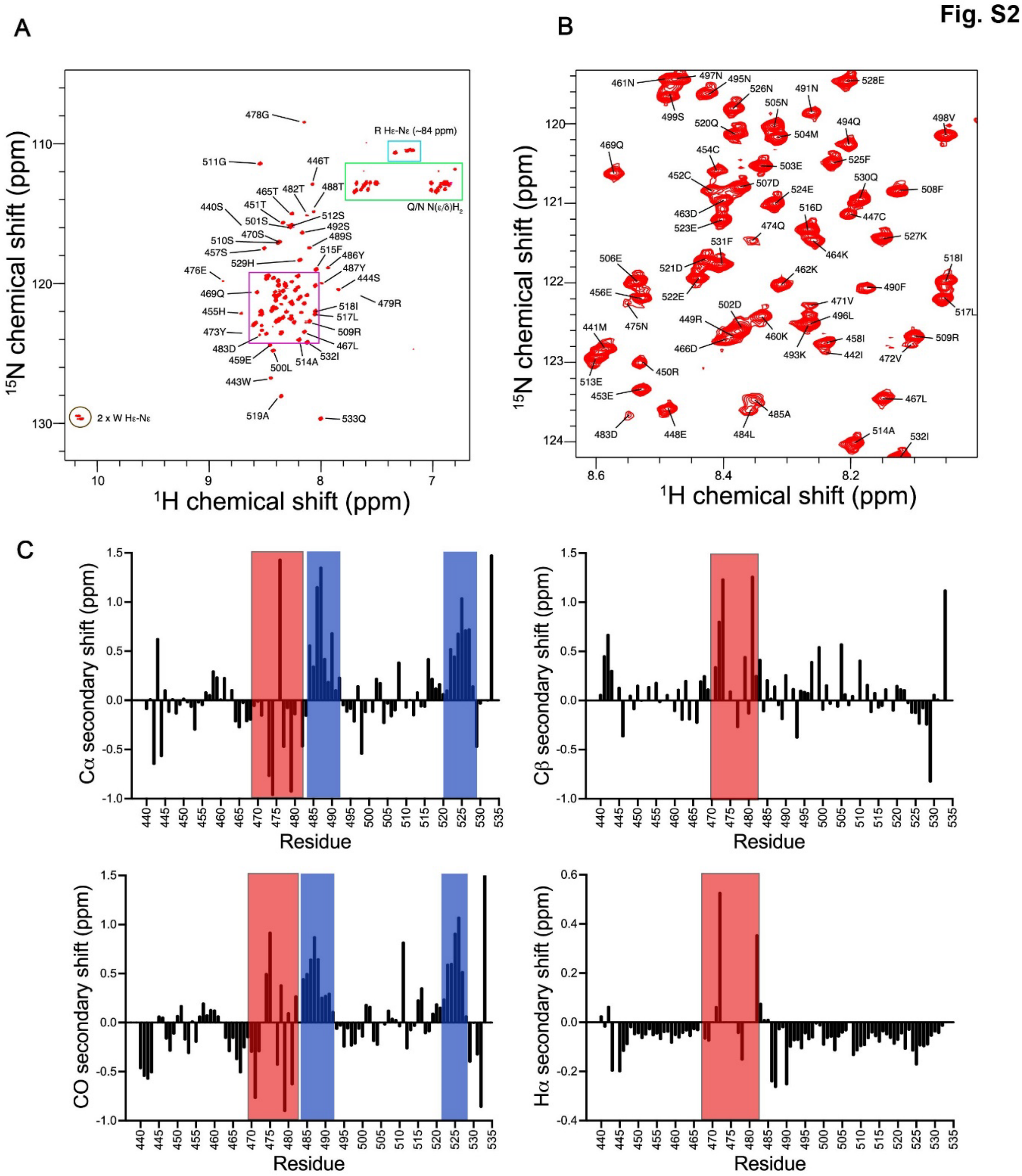

Appendix Figure S2: Full assignment of CEP192 468-533 and further analysis of secondary shifts

A Assignment of the ^{1}H–^{15}N HSQC spectrum for CEP192 (442–533).

B Close up of the centre of the assignment, showing the apo CEP192 spectra in red. ^{15}N-labelled CEP192$_{442\text{-}533}$ appears mostly disordered in solution, as most peaks are clustered near the centre of the ^{1}H-^{15}N HSQC spectrum with poor dispersion in the ^{1}H dimension.

C Plots of Cα, C(O), Cβ and Hα secondary shifts measured for CEP192 442-533 at 10 °C. Positive Cα and carbonyl secondary shifts indicated helical propensity within two regions spanning aa 484–491 and aa 522–528 (shaded in blue). Furthermore, the region close to G478 was predicted to form a β-hairpin based on positive Cβ and Hα, alongside negative Cα, C(O) patterns of the surrounding residues (shaded in red). Assignment spectra were recorded on a 750-MHz Oxford Instruments Magnet equipped with a Bruker Avance III HD console and

a TCI-cryoprobe in NMR-gly buffer (20 mM $(K/H)_2PO_4$, 150 mM NaCl, 1.5% glycerol, pH 6.5) at 10 °C. Regions showing an α-helical propensity are shown in blue, and the region that may form a β-hairpin is shown in red.

Fig. S3

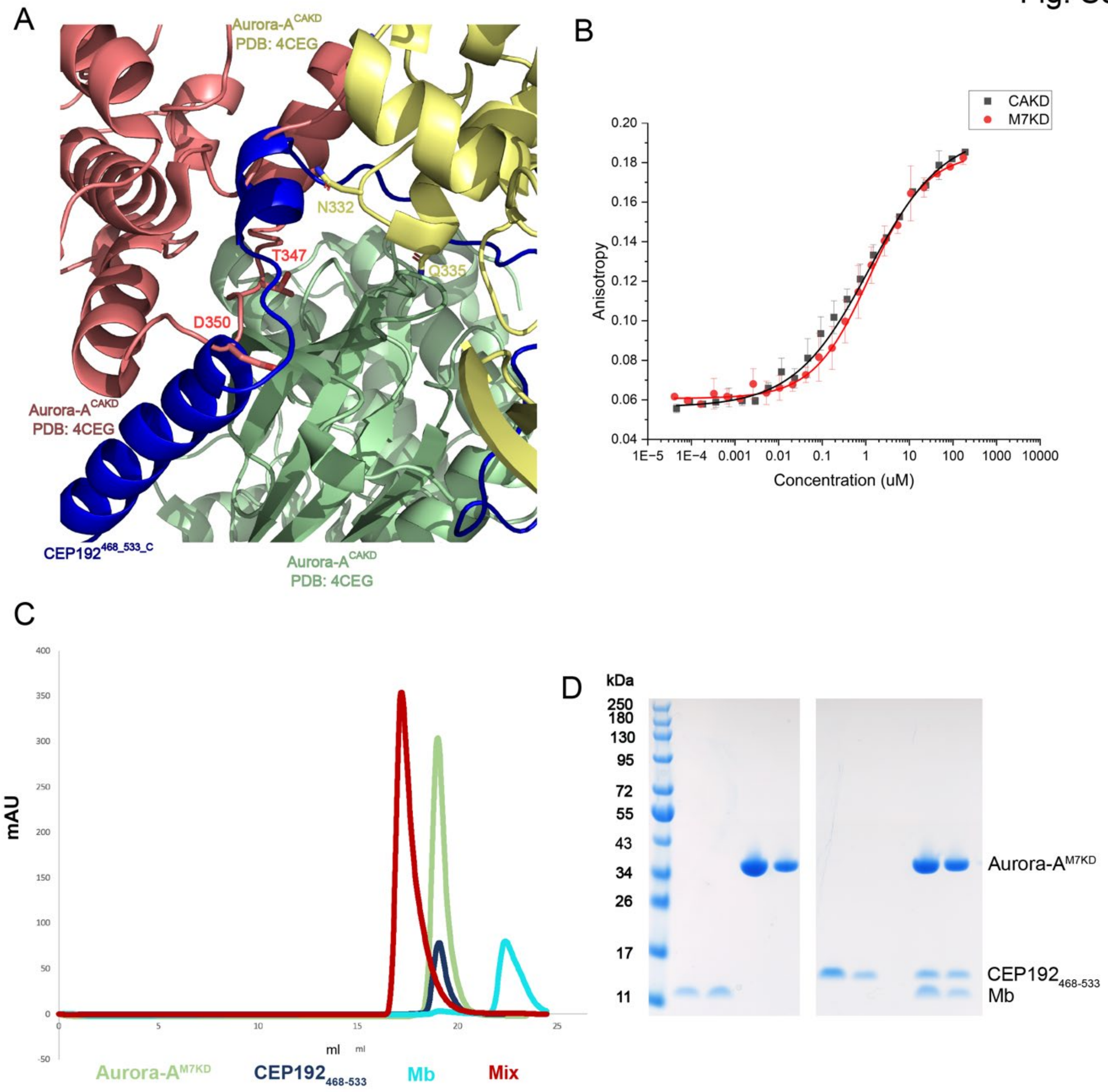

Appendix Figure S3: Mutating Aurora-A and inclusion of a monobody does not affect binding to CEP192

A Mapping of the mutated residues onto the structure of Aurora-A kinase domain (PDB 4CEG, C290A C393A 122-403). All the residues are on the C-lobe and are predicted to prevent crystal contact formation to stop apo-crystal growth. All residues shown are mutated to alanine in Aurora-A^{M7KD} (N332, Q332, T347, D350). Superposition of CEP192$^{468\text{-}533}$ bound to Aurora-A, shows an interaction that overlaps with the crystal contacts in apo-crystals of Aurora-A.

B Direct binding of FITC-CEP192$_{501\text{-}533}$ to Aurora-A^{M7KD} (K_d 1.25 μM ± 0.15 μM) and Aurora-A^{CAKD} (K_d 1.17 μM ± 0.07 μM), showing that the crystal contact mutants do not affect the interaction with CEP192.

C Analytical SEC of Aurora-A^{M7KD} (light green), CEP192 $_{468\text{-}533}$ (dark blue) and the inhibitory monobody (light blue) and all three mixed (red). A clear complex is seen as the elution volume is significantly earlier than any of the proteins alone.

D SDS-PAGE of the separate proteins and the three-way complex formed between Aurora-A^{M7KD}, inhibitory monobody and CEP192$_{468\text{-}533}$.

Data information: In **B**, displayed data points and Kd values represent the average anisotropy for each reaction with the standard deviation for the mean shown as errors bars (n = 3 independent experimental samples).

Fig. S4

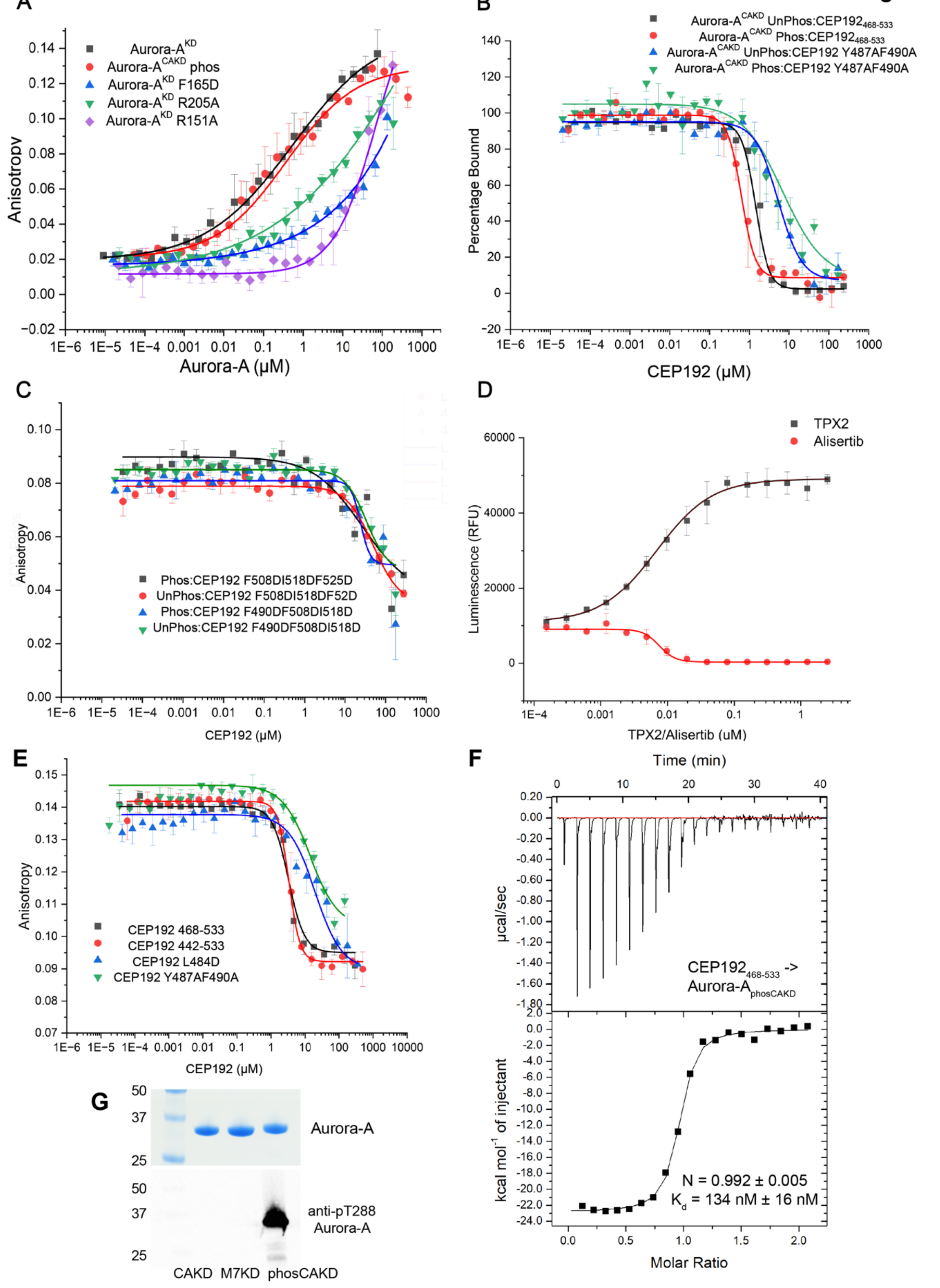

Appendix Figure S4: Testing mutants of Aurora-A and CEP192 in direct binding and competition assays

A Direct binding of Aurora-A^{KD} point mutants (F151A, R205A, F165D) and Aurora-A^{CAKD} phosphorylated protein to FAM-CEP192 500-533. The measured K_d for the dephosphorylated

Aurora-A^{KD} was 420 nM ± 244 nM, whereas for the phosphorylated Aurora-A^{CAKD} it was 370 nM ± 130 nM.

B Fluorescence anisotropy-based competition assay with $CEP192_{468\text{-}533}$ WT and Y487A/F490A competing for binding with FAM-CEP192 500-533 onto either phosphorylated or unphosphorylated Aurora-A^{CAKD}. The IC_{50} for WT $CEP192_{468\text{-}533}$ and unphosphorylated Aurora-A^{CAKD} was 1.42 μM ± 0.56 μM, whilst for phosphorylated Aurora-A^{CAKD} it was 0.63 μM ± 0.12 μM. The IC_{50} for Y487A/F490A and phosphorylated Aurora-A^{CAKD} was 5.39 μM ± 1.55 μM, and to the unphosphorylated Aurora-A^{CAKD} it was 6.9 μM ± 4.4 μM.

C Fluorescence anisotropy-based competition assay with $CEP192_{468\text{-}533}$ F508D/I518D/F525D or F490D/F508D/I518D competing for binding with FAM-CEP192 500-533 and either phosphorylated or unphosphorylated Aurora-A^{CAKD}.

D Control ADP-Glo kinase activity assay, with TPX2 stimulating the activity of Aurora-A^{CAKD} (EC_{50} of 6.8 nM ± 0.5 nM) and Alisertib inhibiting the activity (IC_{50} 3.7 nM ± 0.1 nM).

E Fluorescence anisotropy-base competition assay with CEP192 WT and mutants competing for binding with FAM-TPX2 7-43 onto unphosphorylated Aurora-A^{CAKD}. The IC_{50} for WT $CEP192_{468\text{-}533}$ binding to Aurora-A^{CAKD} was 3.24 μM ± 0.42 μM, whilst for WT $CEP192_{442\text{-}533}$ it was 3.33 μM ± 0.09 μM. The IC_{50} for $CEP192_{468\text{-}533}$ Y487AF490A binding to Aurora-A^{CAKD} was 14.1 μM ± 6.66 μM, and for the $CEP192_{468\text{-}533}$ mutant L484D was 19.9 μM ± 5.18 μM.

F ITC titration of $CEP192_{468\text{-}533}$ into Aurora-$A^{phosCAKD}$. The parameters of the fit are listed.

G SDS-PAGE gel and western blot of versions of Aurora-A used in this study alongside the phosphorylation state. Only the protein that hasn't been co-expressed with lambda phosphatase shows phosphorylation on Thr288 in the activation loop.

Data information: In **A**, **B**, **C** and **E**, displayed data points and IC_{50}/EC_{50}/Kd values represent the average anisotropy for each reaction with the standard deviation for the mean shown as errors bars (n = 3 independent experimental samples).

Displayed data points in **D** and EC_{50} values represent the average luminescence for each reaction condition with standard deviations of the mean as error bars (n = 3 independent experiment samples).

Fig. S5

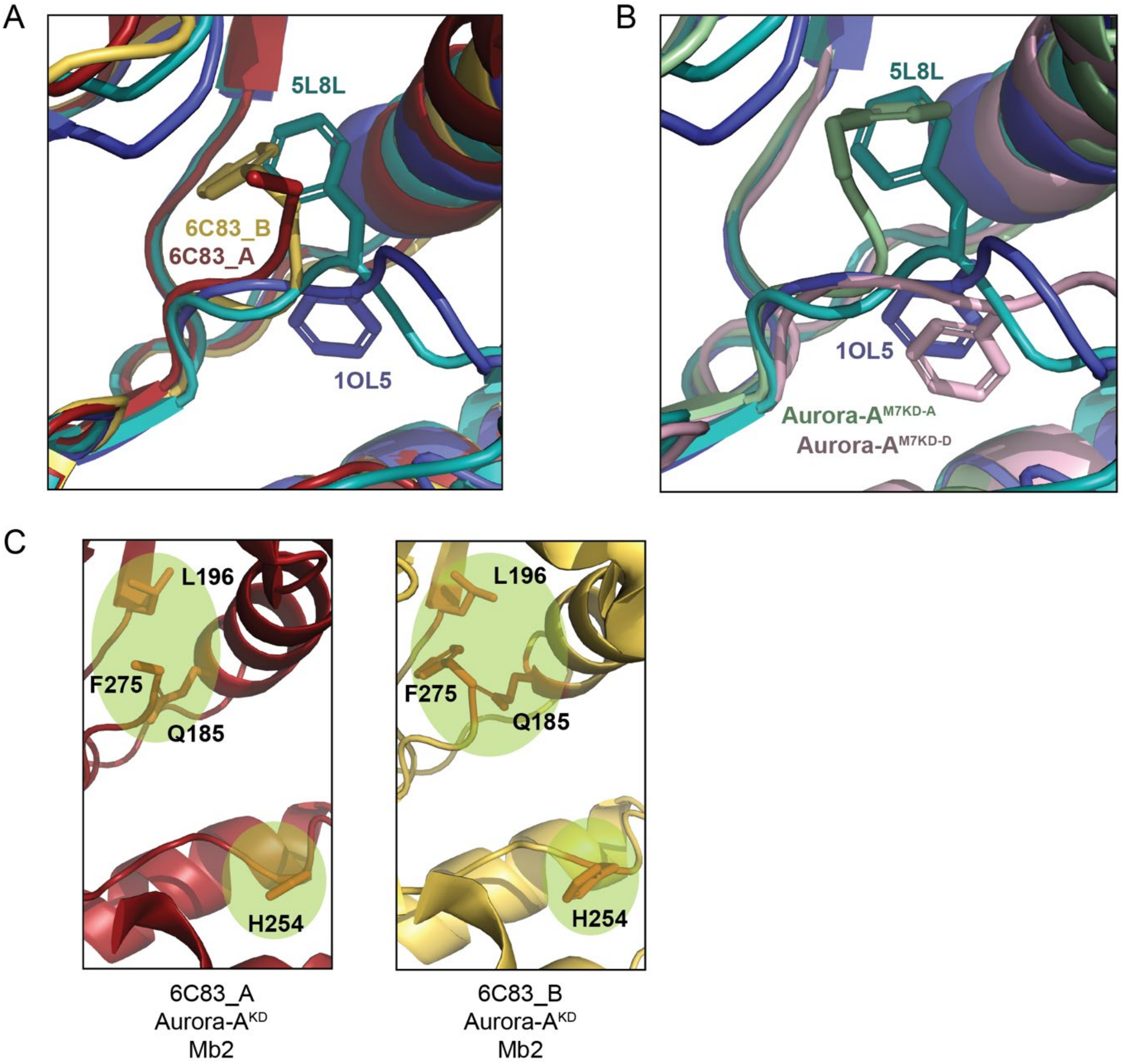

Appendix Figure S5: Further comparisons of DFG-motifs

A Comparison of the DFG motif in the original structure of the inhibitory monobody (Mb2) bound to Aurora-A kinase domain (Zorba et al., 2019). In both copies of Aurora-A in the structure F275 appears to be in the inter position (shown in yellow and maroon), compared to the examples of another DFG-inter structure (5L8L, Aurora-A bound to a VNAR (Burgess *et al*, 2016) shown in teal) and an example of an active structure with DFG-in (1OL5, Aurora-A bound to TPX2 (Bayliss *et al*, 2003) shown in blue).

B Comparison of the DFG motif from the copies of Aurora-A^{M7KD} bound to $CEP192_{468\text{-}533}$. In Aurora-$A^{M7KD\text{-}A}$ chain A, which is modelled bound to $CEP192^{468\text{-}531}$, F275 appears to be closest to the DFG-inter structure (shown in light green). Whereas in the second copy, Aurora-$A^{M7KD\text{-}D}$ bound to just αL of $CEP192_{505\text{-}527}$, F275 appears to be in a DFG-in active position (shown in light pink).

C Comparison of the disrupted R-Spine assembly in the two copies of Aurora-A bound to the inhibitory monobody Mb2 (PDB 6C83 (Zorba *et al*, 2019)).

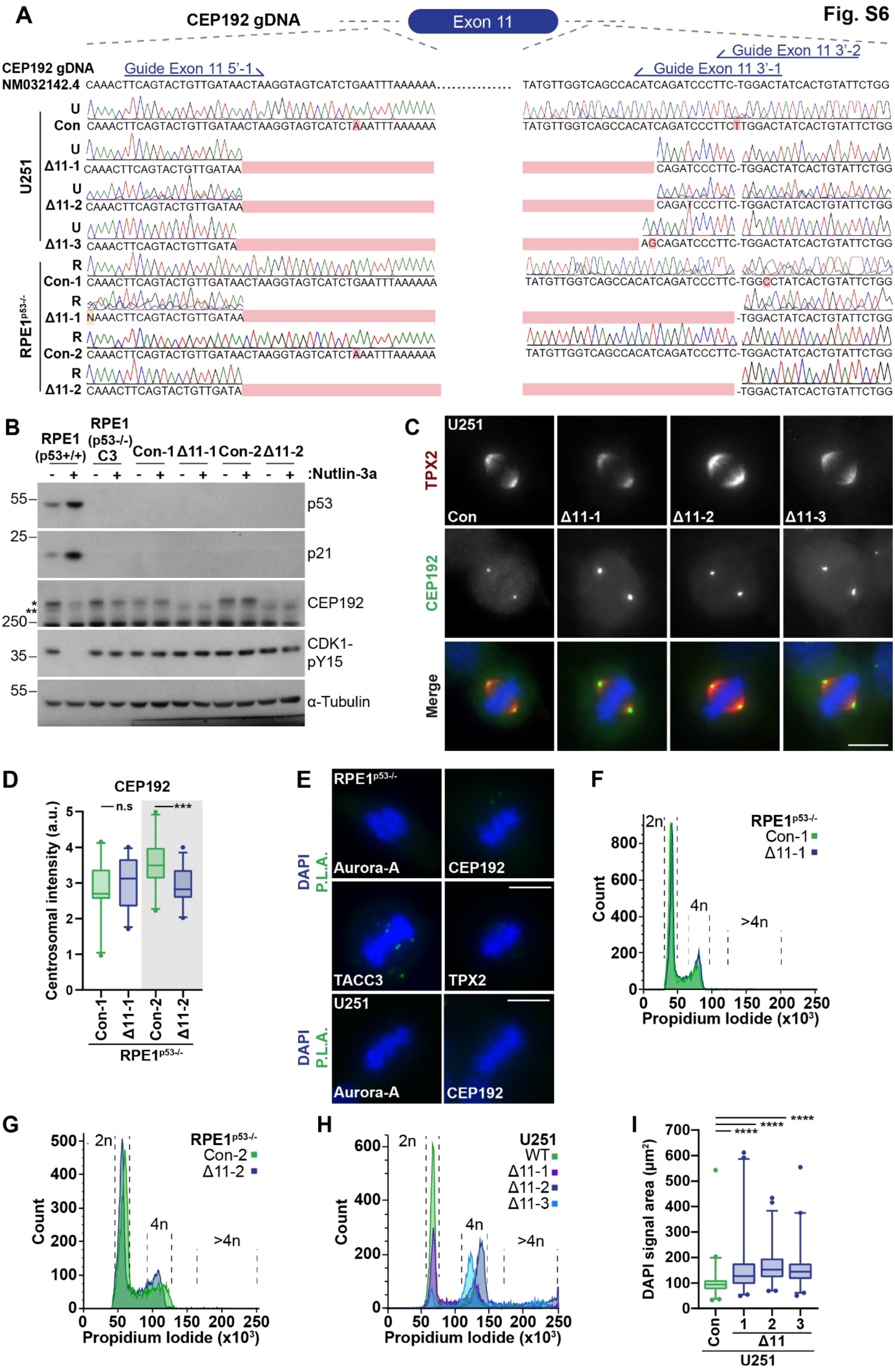
Fig. S6
A
CEP192 gDNA
Exon 11
Guide Exon 11 5'-1
Guide Exon 11 3'-1
Guide Exon 11 3'-2
NM032142.4
U251
RPE1p53-/-
B
RPE1 (p53+/+)
RPE1 (p53-/-) C3
Con-1 Δ11-1 Con-2 Δ11-2
:Nutlin-3a
p53
p21
CEP192
CDK1-pY15
α-Tubulin
C
U251
TPX2
CEP192
Merge
Con
Δ11-1
Δ11-2
Δ11-3
D
CEP192
Centrosomal intensity (a.u.)
n.s
***
RPE1p53-/-
E
RPE1p53-/-
DAPI P.L.A.
Aurora-A
CEP192
TACC3
TPX2
U251
F
RPE1p53-/-
Con-1
Δ11-1
Count
Propidium Iodide (x10³)
2n
4n
>4n
G
RPE1p53-/-
Con-2
Δ11-2
H
U251
WT
Δ11-1
Δ11-2
Δ11-3
I
DAPI signal area (μm²)
****
Con
Δ11
U251

Appendix Figure S6: Characterising the loss of *CEP192(exon11)* in cells

A Sequencing confirming removal of CEP192 exon 11 from U251 and RPE1 gDNA.

B Western blot analysis confirming the p53 status of cell lines following 20 hour treatment with either DMSO control or 2.5 μM Nutlin-3a. RPE1 indicates the parental $p53^{+/+}$ line from which other clones were derived. Con-3 is an additional $RPE1^{p53-/-}$ control cell line generated previously within the lab. A band-shift between CEP192-WT and Δ11 proteins can be observed, these forms are marked with * and **, respectively.

C Immunofluorescence images of control and Δ11 U251 cells stained with antibodies against TPX2 and CEP192 are red and green in merged images, respectively, with DNA stained with DAPI (blue).

D Box plot of CEP192 centrosomal signal intensity in $RPE1^{p53-/-}$ cells, with representative images shown in **5C** (n=2, 10 cells/repeat). Exact p values (L-R): 0.4959, 0.0004.

E Representative immunofluorescence images from proximity ligation assays in $RPE1^{p53-/-}$ and U251 cells. These individual antibody controls were obtained by incubating coverslips with only the indicated primary antibody followed by both of the labelled-secondary antibodies provided.

F-H Representative histogram of propidium iodide intensity as determined by FACS of control and Δ11 **F)** $RPE1^{p53-/-}$ pair 1, **G)** $RPE1^{p53-/-}$ pair 2 or **H)** U251 cells.

I Box plot of DAPI signal area from interphase control and Δ11 U251 cells (n=3, ≥40 cells/repeat, ~200 cells total).

Data information Box plots in **D** and **I** indicate the median and interquartile ranges with coloured bars representing 5th-95th and 1st-99th percentile ranges, respectively. Grey shading in **D** denotes independently completed biological replicates. p values are denoted as follows: **** p<0.0001, *** p<0.001, n.s not significant (Mann-Witney test). Scale bars in **C** and **E** represent 10 μm.

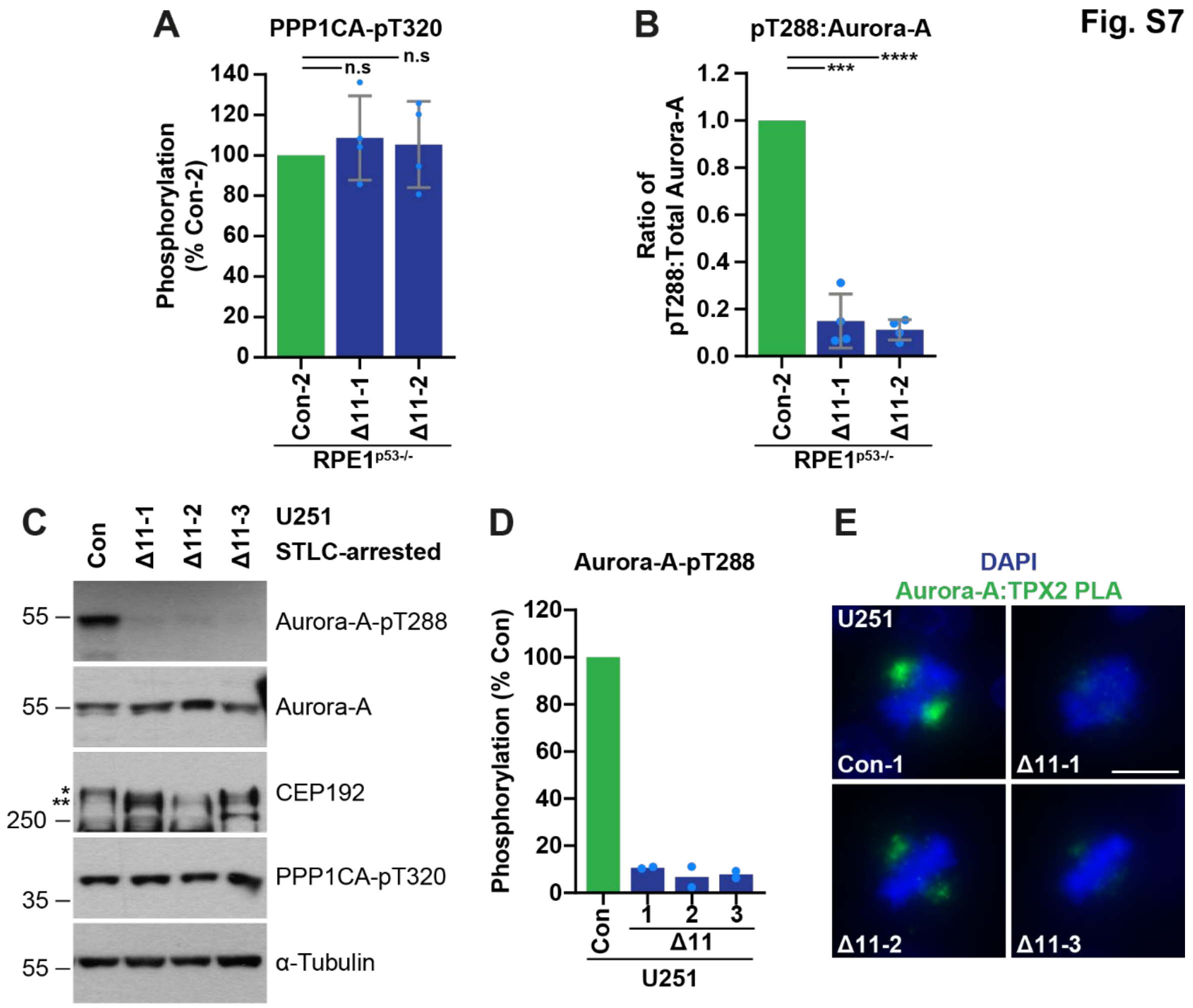

Appendix Figure S7: Binding of Aurora-A to CEP192 is essential for autophosphorylation in U251 cells

A Densitometric quantification of PPP1CA-pT320 signal from **6E** (n=4 biological replicates). Exact p values (L-R): 0.6513, 0.4722.

B Ratio of the pT288:total Aurora-A signals obtained through densitometric quantification of the Aurora-A-pT288 and Aurora-A western blots in **6E** (n=4 biological replicates). Exact p values (L-R): 0.0007, <0.0001.

C Western blot of control and Δ11 U251 cells synchronized in mitosis with 10 μM STLC (20 h). A band-shift between CEP192-WT and Δ11 proteins can be observed, these forms are marked with * and **, respectively.

D Densitometric quantification of Aurora A-pT288 signal from **C** (n=2 biological replicates).

E Proximity ligation assay (PLA) between Aurora-A and TPX2 specific antibodies in control and Δ11 U251 cells. PLA signal is green in merged images with DNA stained with DAPI (blue). Quantification is found in figure **6I**.

Data information Grey bars in **A-B** indicate mean±S.D. Bars in **D** depict the means of data points. p values are denoted as follows: **** p<0.0001, *** p<0.001, n.s not significant (Welch's t-test). Scale bar in **E** represents 10 μm.

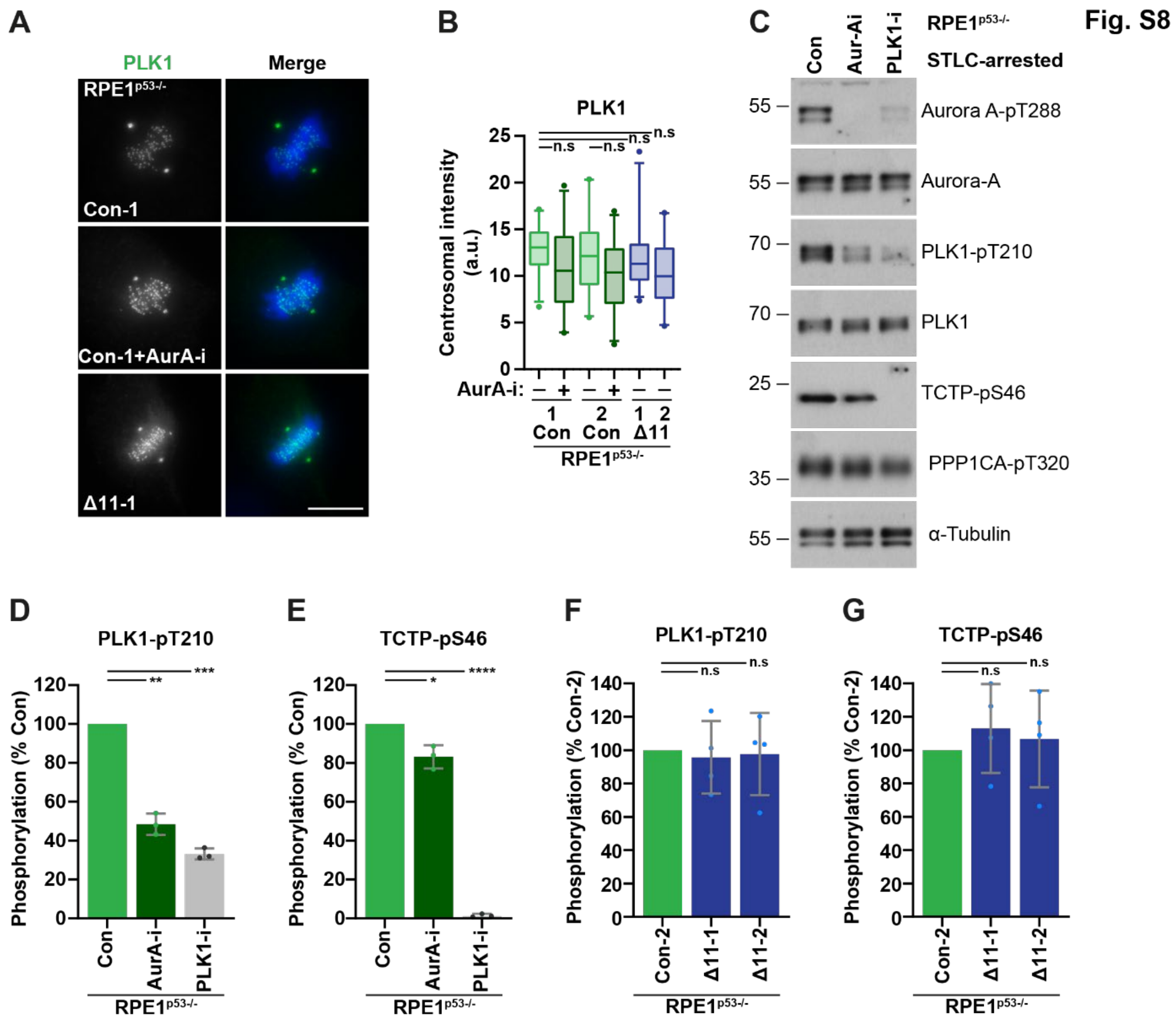

Appendix Figure S8: The activity and localisation of PLK1 is unchanged during mitosis in the absence of Aurora-A:CEP192 complexes

A Immunofluorescence images of control and Δ11 RPE1$^{p53-/-}$ cells treated with either DMSO control or Aurora-A inhibitor (30 min) prior to methanol fixation. An antibody against PLK1 is green in merged images with DNA stained with DAPI (blue).

B Box plot of the PLK1 centrosomal signal intensity in RPE1$^{p53-/-}$ cells, with representative images shown in **A** (n=2, ≥10 cells/biological replicate). Statistical comparison of both control cell lines against the Δ11 clones was determined to be not significant therefore only the comparison with Con-1 is shown for clarity. Note that the brief (30 min) Aurora-A inhibitor treatment does not cause a significant reduction in centrosomal Plk1 levels, albeit there is a negative trend. Exact p values (L-R): 0.3505, >0.9999, >0.9999, 0.09.

C Western blot analysis of RPE1$^{p53-/-}$ cells arrested in mitosis with STLC (20 h), prior to the addition of a proteasome inhibitor, MG-132 (20 min). Cells were then additionally treated with either DMSO control or one of Aurora-A or PLK1 inhibitors for 30 minutes prior to lysis.

D-E Densitometric quantification of **D)** PLK1-pT210 and **E)** TCTP-pS46 signal from **C** (n=3 biological replicates). Exact p values from **D)** (L-R): 0.0038, 0.0006. Exact p values from **E)** (L-R): 0.0392, <0.0001.

F-G Densitometric quantification of **F)** PLK1-pT210 and **G)** TCTP-pS46 signal from figure **6E** (n=4 biological replicates). Exact p values from **F)** (L-R): 0.7349, 0.8626. Exact p values from **G)** (L-R): 0.4018, 0.6741.

Data information Box plot in **B** indicates the median and interquartile ranges (25th-75th percentile) with coloured whiskers representing 5th-95th percentile ranges. Grey bars in **D-G** indicate mean±S.D. p values are denoted as follows: **** $p<0.0001$, *** $p<0.001$, ** $p<0.01$, * $p<0.05$, n.s not significant (**B** Kruskal-Wallis, **D-G** Welch's t-test). Scale bar in A represents 10 μm.

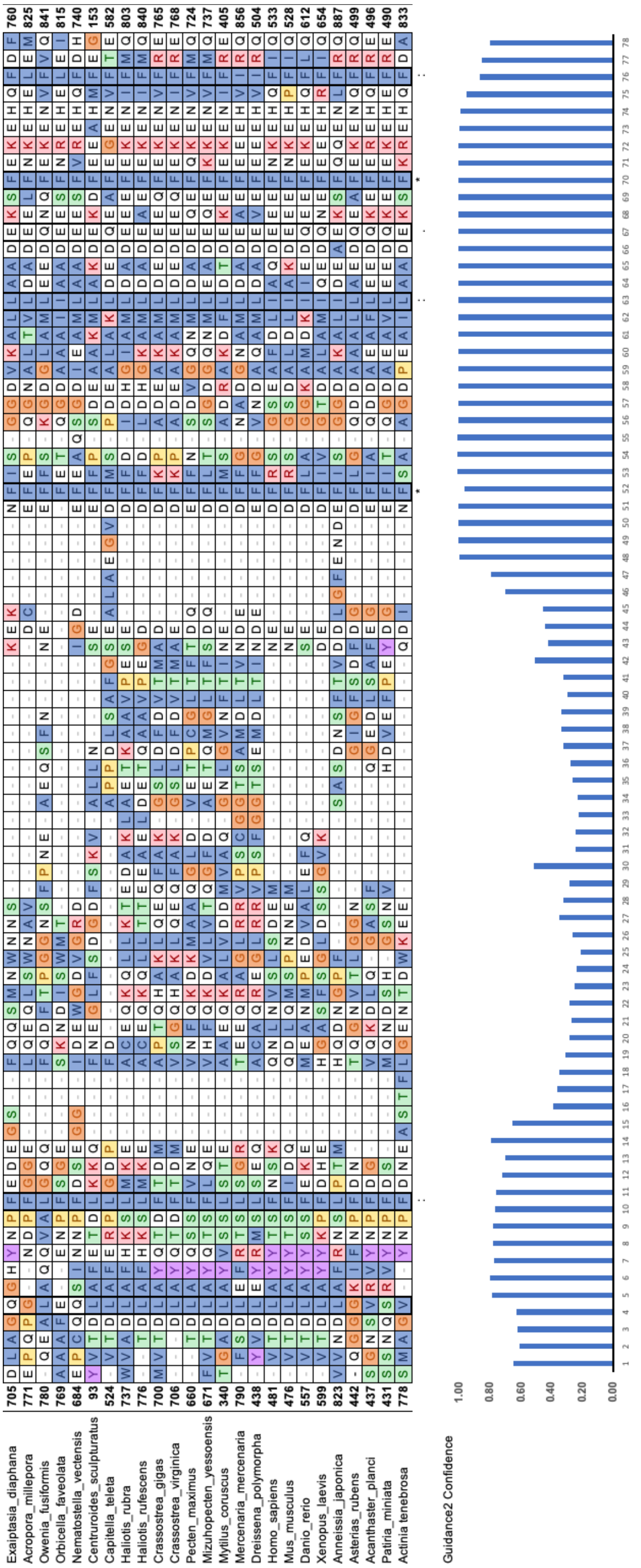

Appendix Figure S9: MAFFT alignment of Aurora-A binding region of CEP192 orthologues.

Residues involved in the interaction are highlighted with black edging. The GUIDANCE2 confidence in the alignment is shown below.

Fig. S10

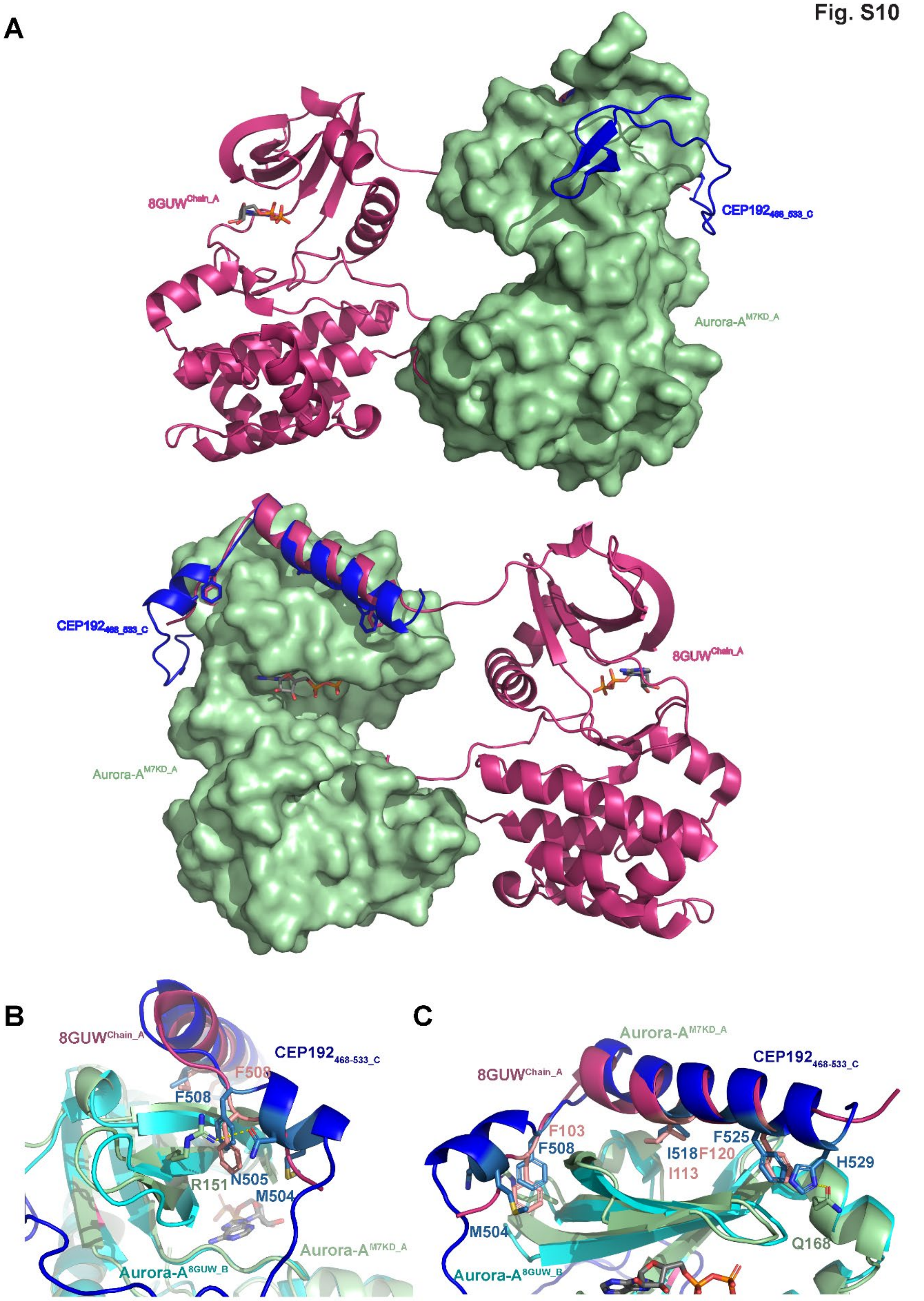

Appendix Figure S10: Comparison of the co-crystal structure with CEP192 fused to Aurora-A

A Comparison of the published structure of CEP192 fused to Aurora-A kinase domain (8GUW, dark pink chain A) with the co-crystallisation of $CEP192_{468-533}$ (dark blue) bound to Aurora-A^{M7KD_A} (light green). Only the αL helix is present in the region of CEP192 fused to Aurora-A. Helix αS is not present in the fused structure due to earlier truncation at residue 506, and the position of Phe508 is shifted.

B Further comparison of the two structures, focusing on the interactions of the αS helix of CEP192 ($CEP192_{468-533}$ in dark blue, 8GUW chain A in dark pink, 8GUW chain B in teal and Aurora-A^{M7KD} in light green). In the context of the co-crystal structure, Asn505 from CEP192 can form a H-bond with Arg151 in Aurora-A (shown as a dashed yellow line), which is not seen in the fused structure.

C Further comparison of the αL helix of CEP192 between the two structures ($CEP192_{468-533}$ in dark blue, 8GUW chain A in dark pink, 8GUW chain B in teal and Aurora-A^{M7KD} in light green). In the co-crystal structure, His529 at the C-terminal end of the αL helix can form a H-bond with Gln168 in the Aurora-A kinase domain (shown as a dashed yellow line), but this residue is not present in the CEP192 fusion construct.

	Aurora-A^{M7KD}:CEP192$_{468-533}$:Mb2
**(PDB accession code)**	8PR7
**Data collection**	DLS i04
Space group	P 4_3 2_1 2
Cell dimensions	
a, *b*, *c* (Å)	112.8, 112.58, 216.31
α, β, γ (°)	90.00, 90.00, 90.00
Resolution (Å)	64.11 – 2.76 (2.81 – 2.76)
R_{sym} or R_{merge}	0.129
R-pim	0.026
$CC_{1/2}$	1.0
Completeness (%)	100 (100)
Redundancy	25.9 (27.4)
Wavelength	0.9537
Observations	947862
**Refinement**	
Resolution (Å)	2.76
No. reflections	34548
R_{work} / R_{free}	0.203 / 0.258
No. atoms	11657
Protein	5830
Ligand/ion	61
Water	89
B-factors	
Protein	106.01
Ligand/ion	133.2
Water	90.48
R.m.s. deviations	
Bond lengths (Å)	0.0089
Bond angles (°)	1.63

(Values in parentheses are for highest-resolution shells)

Appendix Table S1: Data collection and refinement statistics

Model	Chain	Modelled region in structure	Activity label	Spatial label	Dihedral label	Chelix-SaltBridge	R spine	Ligand name:id	Reference
8PR7	A	Aurora-A 122-403 with CEP192 468_533 and Inhibitory Monobody Mb2	Inactive	DFGinter	Unassigned	out-out	InActive HRD out	ADP:401	This paper
8PR7	D	Aurora-A 122-403 with CEP192 506-527 and Inhibitory Monobody Mb2	Inactive	DFGin	BLBminus	out-none	Active	ADP:401	This paper
6C83	A	Aurora-A 122-403 with Inhibitory Monobody Mb2	Inactive	None	Unassigned	out-none	InActive HRD out	ACP:401	(Zorba *et al.*, 2019)
6C83	B	Aurora-A 122-403 with Inhibitory Monobody Mb2	Inactive	DFGinter	Unassigned	out-out	InActive HRD out	ACP:401	(Zorba *et al.*, 2019)
1OL5	A	Aurora-A 122-403 with TPX2 1-43	Active	DFGin	BLAminus	in-in	Active	ADP:1388	(Bayliss *et al.*, 2003)
8GUW	A	CEP192 506-527 fused to Aurora-A 123-403	Inactive	DFGin	ABAminus	out-out	Active	ADP:501	(Park *et al*, 2023)
8GUW	C	CEP192 506-527 fused to Aurora-A 123-403	Inactive	DFGin	ABAminus	out-out	Active	ADP:501	(Park *et al*, 2023)
8GUW	B	CEP192 506-527 fused to Aurora-A 123-403	Inactive	DFGin	ABAminus	out-out	Active	ADP:501	(Park *et al*, 2023)
6CPG	A	Aurora-A 122-403 with Inhibitory Monobody Mb2	Inactive	DFGinter	Unassigned	out-out	InActive HRD out	AT9283	(Pitsawong *et al*, 2018)
6CPG	D	Aurora-A 122-403 with Inhibitory Monobody Mb2	Inactive	DFGinter	Unassigned	out-out	InActive HRD out	AT9283	(Pitsawong *et al.*, 2018)

Appendix Table S2: Summary of the KinCoRe analysis of Aurora-A bound to CEP192, in comparison to published structures of Aurora-A.

**Ligand 1**	**Ligand 2**	**Competitor**	**Value**	**Exp**	**Figure**
FAM-CEP192 $_{501\text{-}533}$	Aurora-A^{CAKD} unphos	-	K_d 420 nM ± 244 nM	FA	S4A
FAM-CEP192 $_{501\text{-}533}$	Aurora-A^{CAKD} phos	-	K_d 370 nM ± 130 nM	FA	S4A
FAM-CEP192 $_{501\text{-}533}$	Aurora A^{KD} R151A	-	Kd > 5 µM	FA	S4A
FAM-CEP192 $_{501\text{-}533}$	Aurora-A^{KD} R205A	-	Kd > 1 µM	FA	S4A
FAM-CEP192 $_{501\text{-}533}$	Aurora-A^{KD} F165D	-	Kd > 5 µM	FA	S4A
FAM-CEP192 $_{501\text{-}533}$	Aurora-A^{M7KD}	-	Kd 1.25 µM ± 0.15 µM	FA	S3D
FAM-CEP192 $_{501\text{-}533}$	Aurora-A^{CAKD} unphos	-	Kd 1.17 µM ± 0.07µM	FA	S3D
CEP192$_{442\text{-}533}$	Aurora-A^{CAKD} D274N	-	K_d 72 nM ± 20 nM	ITC	EV2B
CEP192$_{468\text{-}533}$	Aurora-A^{CAKD} D274N	-	K_d 80.6 nM ± 21 nM	ITC	3C
CEP192$_{468\text{-}533}$	Aurora-A^{KD} F165DR205A	-	K_d 26 µM ± 16 µM	ITC	3F
CEP192$_{468\text{-}533}$ Y487AF490A	Aurora-A^{CAKD}	-	K_d 2 µM ± 0.1 µM	ITC	3D
CEP192$_{468\text{-}533}$ F490DF508DI518D	Aurora-A^{CAKD}	-	-	ITC	3E
CEP192$_{468\text{-}533}$	Aurora-A^{CAKD} phos	-	Kd 134 nM ± 16 nM	ITC	S4F
FAM-CEP192 $_{501\text{-}533}$	Aurora-A^{CAKD} unphos	CEP192$_{468\text{-}533}$ Y487AF490A	IC_{50} 5.39 µM ±1.5 µM	Comp FA	S4B
FAM-CEP192 $_{501\text{-}533}$	Aurora-A^{CAKD} phos	CEP192$_{468\text{-}533}$ Y487AF490A	IC_{50} 6.92 µM ± 4.3 µM	Comp FA	S4B
FAM-CEP192 $_{501\text{-}533}$	Aurora-A^{CAKD} unphos	CEP192$_{468\text{-}533}$	IC_{50} 0.63 µM ± 0.122 µM	Comp FA	S4B
FAM-CEP192 $_{501\text{-}533}$	Aurora-A^{CAKD} phos	CEP192$_{468\text{-}533}$	IC_{50} 1.42 µM ± 0.56 µM	Comp FA	S4B
FAM-CEP192 $_{501\text{-}533}$	Aurora-A^{CAKD} unphos	CEP192$_{468\text{-}533}$ F490DF508DI518D	IC_{50} > 10 µM	Comp FA	S4C
FAM-CEP192 $_{501\text{-}533}$	Aurora-A^{CAKD} phos	CEP192$_{468\text{-}533}$ F490DF508DI518D	IC_{50} > 10 µM	Comp FA	S4C
FAM-CEP192 $_{501\text{-}533}$	Aurora-A^{CAKD} unphos	CEP192$_{468\text{-}533}$ F508DI518DF525D	IC_{50} > 10 µM	Comp FA	S4C
FAM-CEP192 $_{501\text{-}533}$	Aurora-A^{CAKD} phos	CEP192$_{468\text{-}533}$ F508DI518DF525D	IC_{50} > 10 µM	Comp FA	S4C

FAM-TPX2 $_{7\text{-}43}$	Aurora-A^{CAKD}	CEP192$_{442\text{-}533}$	IC$_{50}$ 4.2 μM ± 0.42 μM	Comp FA	2G
FAM-TPX2 $_{7\text{-}43}$	Aurora-A^{CAKD}	CEP192$_{468\text{-}533}$	IC$_{50}$ 3.7 μM ± 0.4 μM	Comp FA	2G
FAM-TPX2 $_{7\text{-}43}$	Aurora-A^{CAKD}	CEP192$_{501\text{-}533}$	-	Comp FA	2G
FAM-TACC3 $_{522\text{-}536}$	Aurora-A^{CAKD}	CEP192$_{442\text{-}533}$	IC$_{50}$ 5.7 μM ± 1 μM	Comp FA	2H
FAM-TACC3 $_{522\text{-}536}$	Aurora-A^{CAKD}	CEP192$_{468\text{-}533}$	IC$_{50}$ 5 μM ± 0.5 μM	Comp FA	2H
FAM-TACC3 $_{522\text{-}536}$	Aurora-A^{CAKD}	CEP192$_{501\text{-}533}$	IC$_{50}$ 29 μM ± 3 μM	Comp FA	2H
FAM-TPX2 $_{7\text{-}43}$	Aurora-A^{CAKD}	CEP192$_{468\text{-}533}$ Y487AF490A	IC$_{50}$ 14.1 μM ± 6.6 μM	Comp FA	S4E
FAM-TPX2 $_{7\text{-}43}$	Aurora-A^{CAKD}	CEP192$_{468\text{-}533}$ L484D	IC$_{50}$ 19.9 μM ± 5 μM	Comp FA	S4E
Aurora-A^{CAKD} phos	-	CEP192$_{468\text{-}533}$	IC$_{50}$ 18 nM ± 5 nM	ADP-Glo	4A
Aurora-A^{CAKD} phos	-	CEP192$_{442\text{-}533}$	IC$_{50}$ 32 nM ± 7 nM	ADP-Glo	4A
Aurora-A^{CAKD} phos	-	CEP192$_{500\text{-}533}$	-	ADP-Glo	4A
Aurora-A^{CAKD} phos	-	CEP192$_{468\text{-}533}$ Y487AF490A	IC$_{50}$ > 1 μM	ADP-Glo	4A
Aurora-A^{CAKD} phos	-	CEP192$_{468\text{-}533}$ L484D	IC$_{50}$ 222 nM ± 44 nM	ADP-Glo	4A
Aurora-A^{CAKD} phos	-	TPX2$_{1\text{-}43}$	EC$_{50}$ 6.8 nM ± 0.5 nM	ADP-Glo	S4D
Aurora-A^{CAKD} phos	-	Alisertib	IC$_{50}$ 3.7 nM ± 0.1 nM	ADP-Glo	S4D

Appendix Table S3: Summary of biophysical assays

Purpose	Sequence
Subcloning CEP192 442-533 into petSUMO	5' gatacggatccatgatttggtcaccaacttgtgaaaggcgaaca 3'<br>5' ctccaactcgagtcactgtataaattgatgctctttattaaa 3'
Subcloning CEP192 468-533 into petSUMO	5' ggtccggatccatgccacagagtgtggtctatcaaaatgaa 3'<br>5' ctccaactcgagtcactgtataaattgatgctctttattaaa 3'
CEP192 468-533 Q5 mutagenesis Y487AF490A	5' tctgctaatagcaaacaaaatttaaatgtgtc 3'<br>5' tgtggcataggcaaggtctgtgac 3'
CEP192 468-533 Quikchange mutagenesis F490D F508D I518D	**F490D**<br>5' gacacatttaaattttgtttgctattatcagatgtgtaataggcaaggtctgtgac 3'<br>5' gtcacagaccttgcctattacacatctgataatagcaaacaaaatttaaatgtgtc 3'<br>**F508D**<br>5' caaatgcttcagaaccagatctgtcgtcttcattcatctcatcactta 3'<br>5' taagtgatgagatgaatgaagacgacagatctggttctgaagcatttg 3'<br>**I518D**<br>5' attaaattcttcttcatcttgtgcatccaaatcaaatgcttcagaaccagatc 3'<br>5' gatctggttctgaagcatttgatttggatgcacaagatgaagaagaatttaat 3'
CEP192 468-533 Quikchange mutagenesis F508D I518D F525D	**F508D**<br>5' caaatgcttcagaaccagatctgtcgtcttcattcatctcatcactta 3'<br>5' taagtgatgagatgaatgaagacgacagatctggttctgaagcatttg 3'<br>**I518D**<br>5' attaaattcttcttcatcttgtgcatccaaatcaaatgcttcagaaccagatc 3'<br>5' gatctggttctgaagcatttgatttggatgcacaagatgaagaagaatttaat 3'<br>**F525D on top of other two mutations**<br>5' cactgtataaattgatgctctttattatcttcttcttcatcttgtgcatccaaatc 3'<br>5' gatttggatgcacaagatgaagaagaagataataaagagcatcaatttatacagtg 3'
CEP192 468-533 Q5 mutagenesis L484D	**L484D**<br>5' ggtcacagacgatgcctattacac 3'<br>5' cacctaccctcttcattttg 3'
Aurora-A 122-403 Quikchange mutagenesis S155R	**S155R**<br>5' agaaaagcaaagaaagtttattctg 3'<br>5' cttgccaaataaacattacc 3'
Aurora-A 122-403 Quikchange mutagenesis F165D	**F165D**<br>5' gctttctccagctgagctttatctaacactttaagagccagaataaacttgc 3'<br>5' gcaagtttattctggctcttaaagtgttagataaagctcagctggagaaagc 3'
Aurora-A 122-403 N332A Q335A T347A D350A C290A C393A D274N for use in crystallography, Quikchange mutagenesis (Q335A C290A C393A mutants already present in library construct)	**N332A over Q335A**<br>5' gtaggtctctgcgtatgtggctgcctcaaaaggaggcttc 3'<br>5' gaagcctccttttgaggcagccacatacgcagagacctac 3'<br>**T347A D350A**<br>5' ccctctgttacaaaggcagggaatgcgaattcaacccgtga 3'<br>5' tcacgggttgaattcgcattccctgcctttgtaacagaggg 3'<br>**D274N**<br>5' tactgaccacccaaaatttgcaattttaagctctccagct 3'<br>5' agctggagagcttaaaattgcaaattttgggtggtcagta 3'

CEP192 Exon 11_5'_1<br>gRNA sequence used to generate CEP192	TTCAGTACTGTTGATAACTA
CEP192 Exon 11_3'_1<br>gRNA sequence used to generate CEP192	AGTCCAGAAGGGATCTGATG
CEP192 Exon 11_3'_2<br>gRNA sequence used to generate CEP192	AATACAGTGATAGTCCAGAA
CEP192 Exon 11 Screening Primer F	GTACAGGGAGGATTGCTCAGAAATGG
CEP192 Exon 11 Screening Primer R	CAGGACATTTCCTTTGCTCAGGTCAC

Appendix Table S4: Primers used within this study

Appendix References

Bayliss R, Sardon T, Vernos I, Conti E (2003) Structural basis of Aurora-A activation by TPX2 at the mitotic spindle. *Mol Cell* 12: 851-862

Burgess SG, Oleksy A, Cavazza T, Richards MW, Vernos I, Matthews D, Bayliss R (2016) Allosteric inhibition of Aurora-A kinase by a synthetic vNAR domain. *Open Biol* 6: 160089

Park JG, Jeon H, Shin S, Song C, Lee H, Kim NK, Kim EE, Hwang KY, Lee BJ, Lee IG (2023) Structural basis for CEP192-mediated regulation of centrosomal AURKA. *Sci Adv* 9: eadf8582

Pitsawong W, Buosi V, Otten R, Agafonov RV, Zorba A, Kern N, Kutter S, Kern G, Padua RA, Meniche X *et al* (2018) Dynamics of human protein kinase Aurora A linked to drug selectivity. *Elife* 7

Zorba A, Nguyen V, Koide A, Hoemberger M, Zheng Y, Kutter S, Kim C, Koide S, Kern D (2019) Allosteric modulation of a human protein kinase with monobodies. *Proc Natl Acad Sci U S A* 116: 13937-13942
