# Supplementary material for: CEP192 localises mitotic Aurora-A activity by priming its interaction with TPX2
Source: EMBO J. 2024 Sep 26;43(22):5381–420. doi: 10.1038/s44318-024-00240-z (PMC11574021; doi:10.1038/s44318-024-00240-z)
Supplement: Supplementary file 3 — Source data Fig. 1 [file 44318_2024_240_MOESM3_ESM.zip › Figure 1/1A/Source data_Figure 1A_Western blots.pdf]

Source Data Figure 1A

1A Western blots. Region of interest was rotated as required to crop a horizontal row of bands.
NB/ The lane marked with * corresponds to a test for an additional siTPX2 oligonucleotide, which was not used within this study.

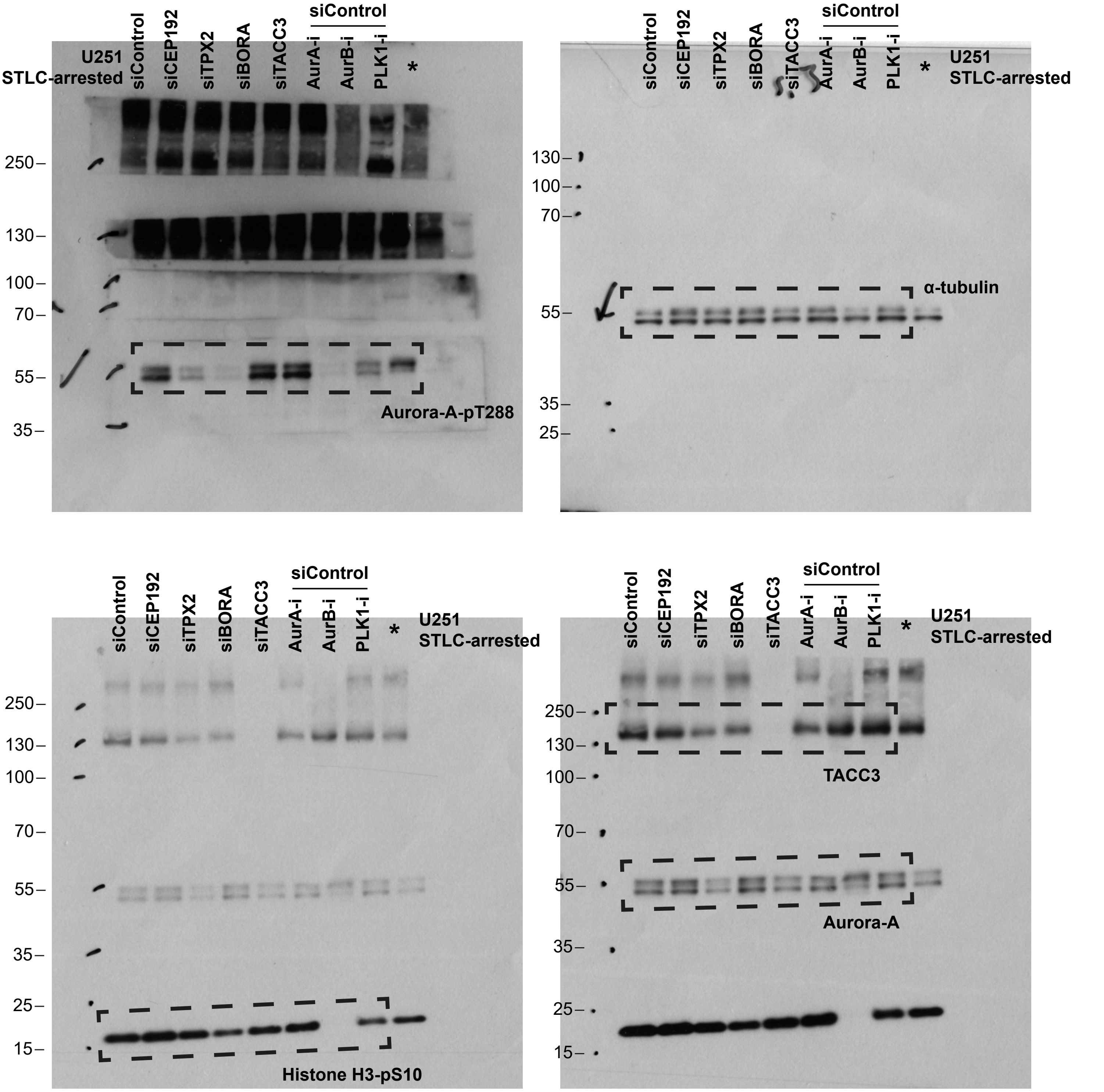

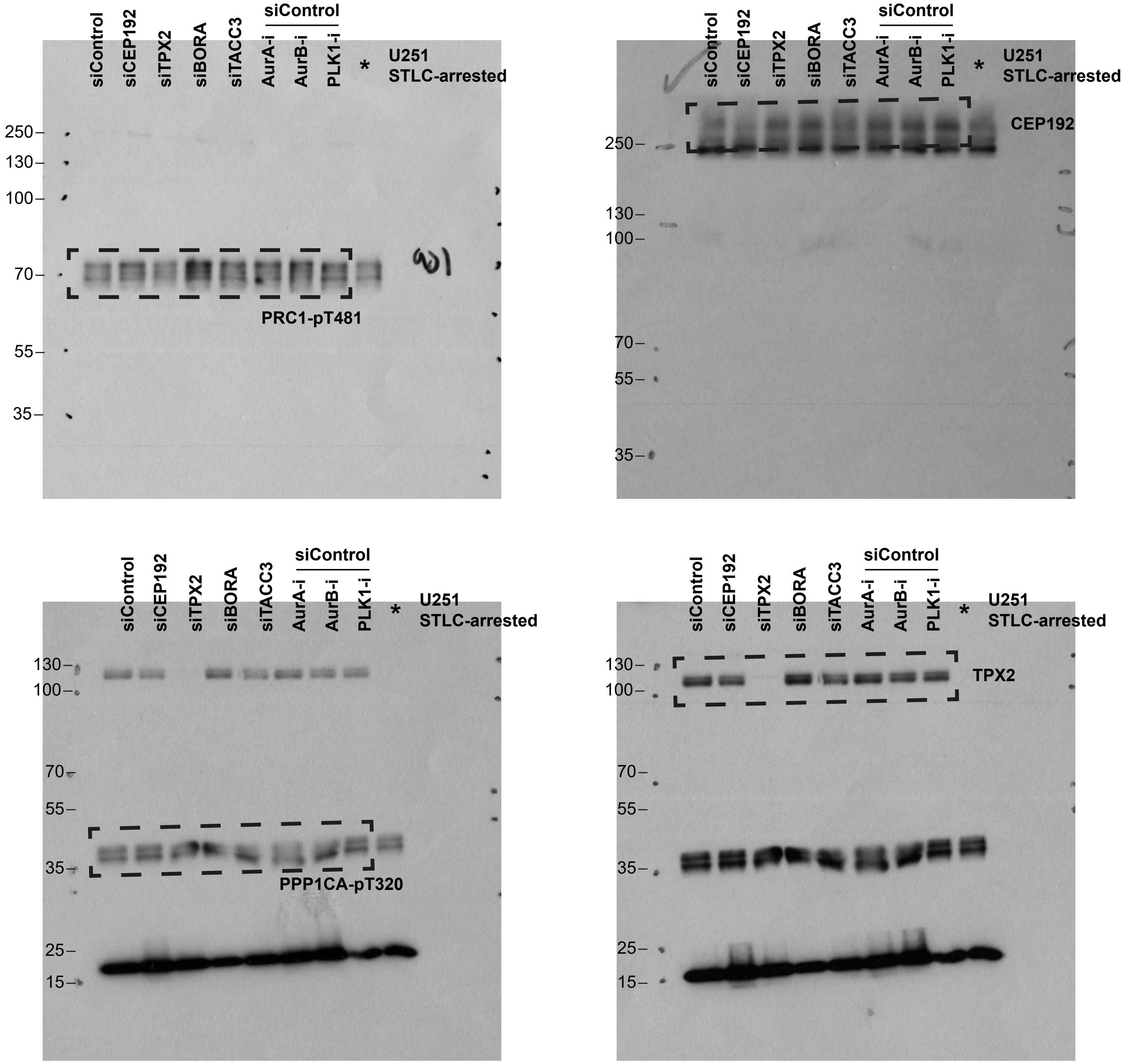
siControl
siCEP192
siTPX2
siBORA
siTACC3
siControl
AurA-i
AurB-i
PLK1-i
*
U251
STLC-arrested
250
130
100
70
55
35
PRC1-pT481
CEP192
PPP1CA-pT320
TPX2
25
15
