# Supplementary material for: CEP192 localises mitotic Aurora-A activity by priming its interaction with TPX2
Source: EMBO J. 2024 Sep 26;43(22):5381–420. doi: 10.1038/s44318-024-00240-z (PMC11574021; doi:10.1038/s44318-024-00240-z)
Supplement: Supplementary file 4 — Source data Fig. 2 [file 44318_2024_240_MOESM4_ESM.zip › Figure 2/2G/Source_Data_Fig_2G_Peptides.docx]

**FAM-Ahx-TPX2(7-43)**

Sequence: FAM-Ahx-SYSYDAPSDFINFSSLDDEGDTQNIDSWFEEKANLEN

HR-MS (ESI) m/z: [M+H]^+^ Calculated= 4732.9591, Found: 4732.9937

HR-QToF(ESI)MS analysis


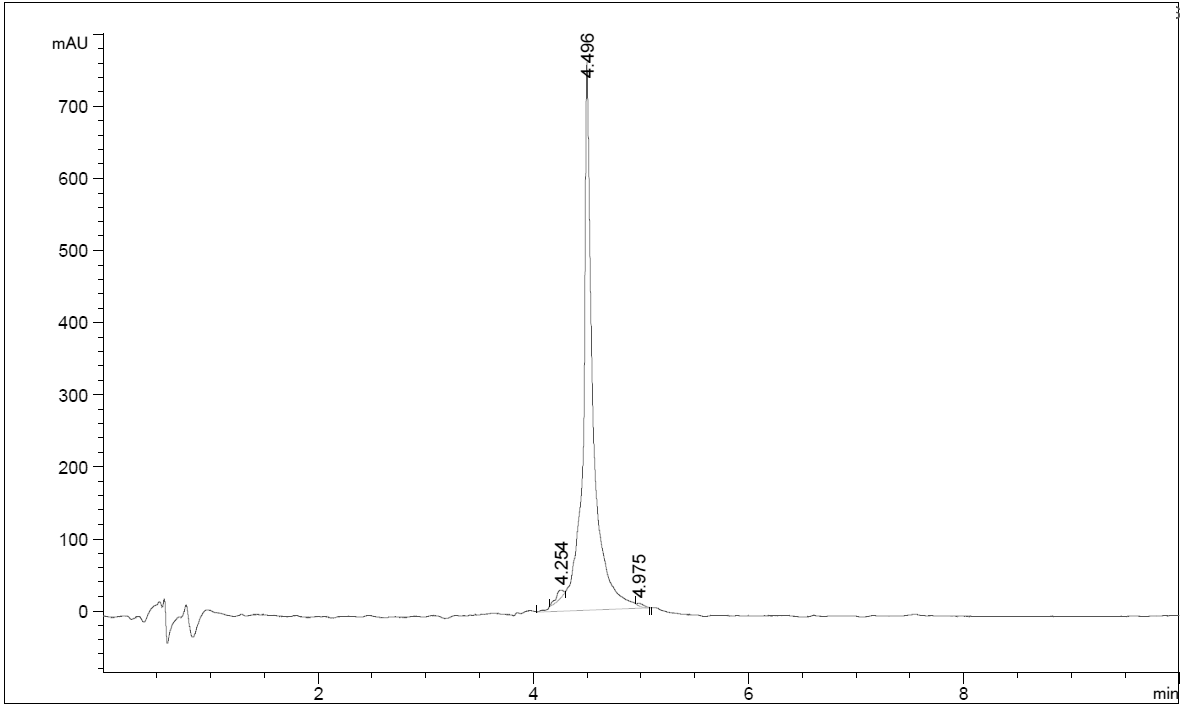


Analytical HPLC trace at λ= 220 nm

**FAM-Ahx-TACC3(522-536)**

Sequence: FAM-Ahx-EESFRDPAEVLGTGA

HR-MS (ESI) m/z: [M+H]^+^ Calculated= 2047.8920, Found: 2047.8911


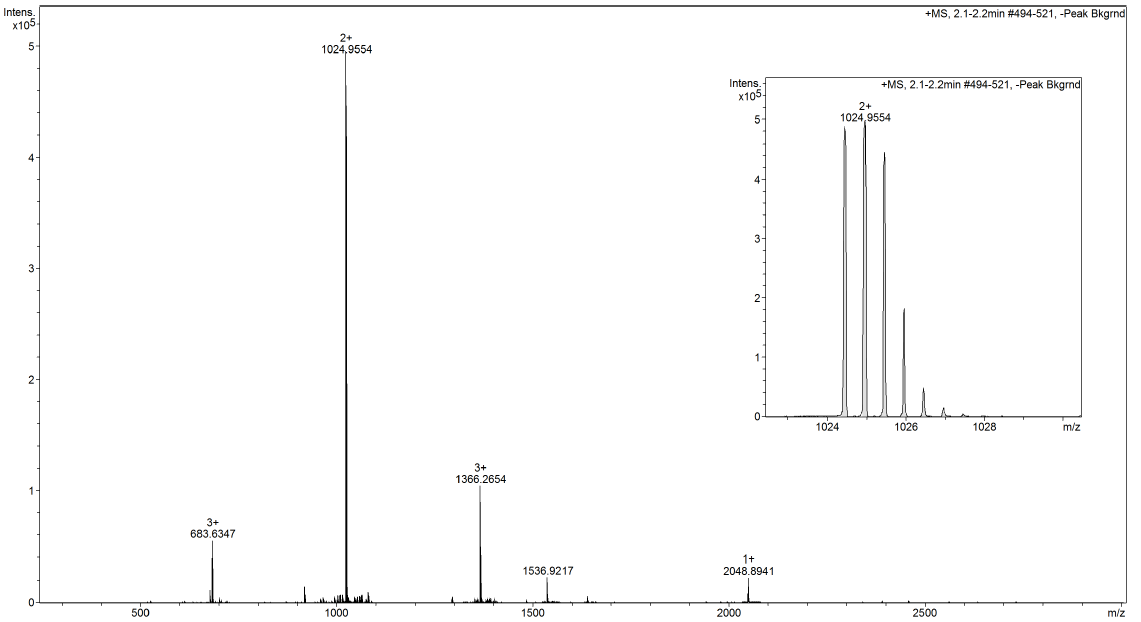


HR-QToF(ESI+)MS analysis of purified FAM-Ahx-TACC3 _522-536_

Analytical HPLC trace at λ= 220 nm of purified FAM-Ahx-TACC3 _522-536_
