# Supplementary material for: CEP192 localises mitotic Aurora-A activity by priming its interaction with TPX2
Source: EMBO J. 2024 Sep 26;43(22):5381–420. doi: 10.1038/s44318-024-00240-z (PMC11574021; doi:10.1038/s44318-024-00240-z)

Source Data Figure 6E

6E Western blots. Region of interest was rotated as required to crop a horizontal row of bands.

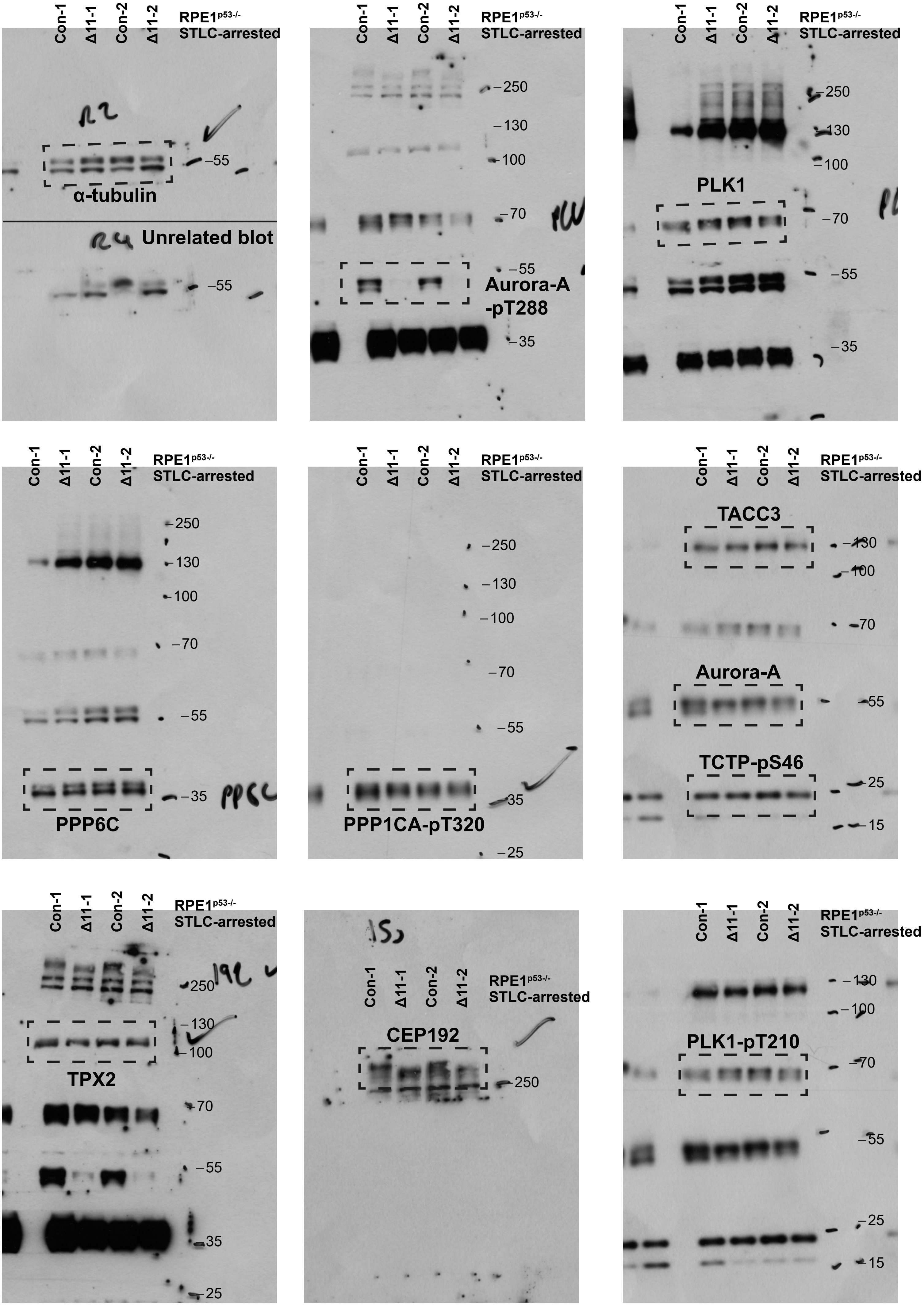

Supplement: Supplementary file 8 — Source data Fig. 6 [file 44318_2024_240_MOESM8_ESM.zip › Figure 6/6E/Source data_Figure 6E_Western blots.pdf]
