# Supplementary material for: CEP192 localises mitotic Aurora-A activity by priming its interaction with TPX2
Source: EMBO J. 2024 Sep 26;43(22):5381–420. doi: 10.1038/s44318-024-00240-z (PMC11574021; doi:10.1038/s44318-024-00240-z)
Supplement: Supplementary file 10 — EV and Appendix Figure Source Data [file 44318_2024_240_MOESM10_ESM.zip › Appendix/S3/S3C/Source_Data_Fig_S3C_Peptide.docx]

**FAM-Ahx -CEP192(501-533)**

Sequence: FAM-Ahx- SDEMNEDFRSGSEAFDLIAQDEEEFNKEHQFIQ

HR-MS (ESI) m/z: [M+H]^+^ Calculated= 4404.8467, Found: 4404.8875

HR-QToF(ESI)MS analysis


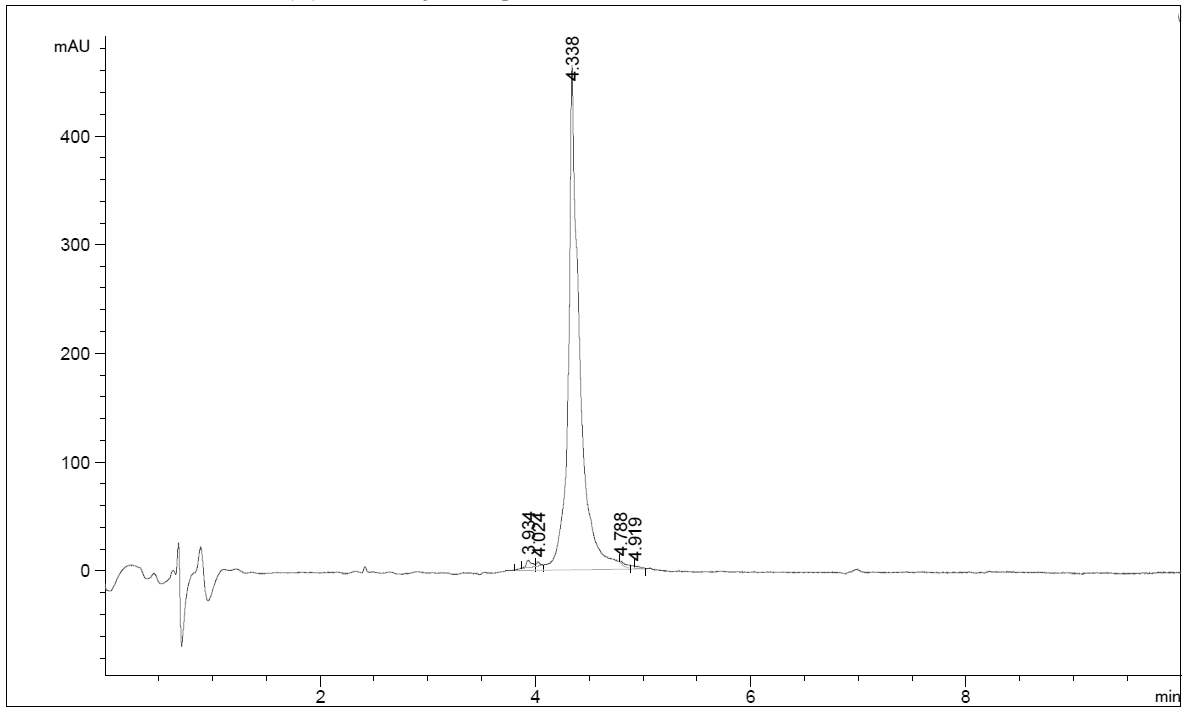


Analytical HPLC trace at λ= 220 nm
