# Supplementary material for: CEP192 localises mitotic Aurora-A activity by priming its interaction with TPX2
Source: EMBO J. 2024 Sep 26;43(22):5381–420. doi: 10.1038/s44318-024-00240-z (PMC11574021; doi:10.1038/s44318-024-00240-z)

Source Data Appendix Figure S7C

S7C Western blots. Region of interest was rotated as required to crop a horizontal row of bands.
NB/ The lanes marked with * correspond to additional U251 clones not used within this study. Lanes marked with an X indicate an immuno-precipitation experiment not included in this study.

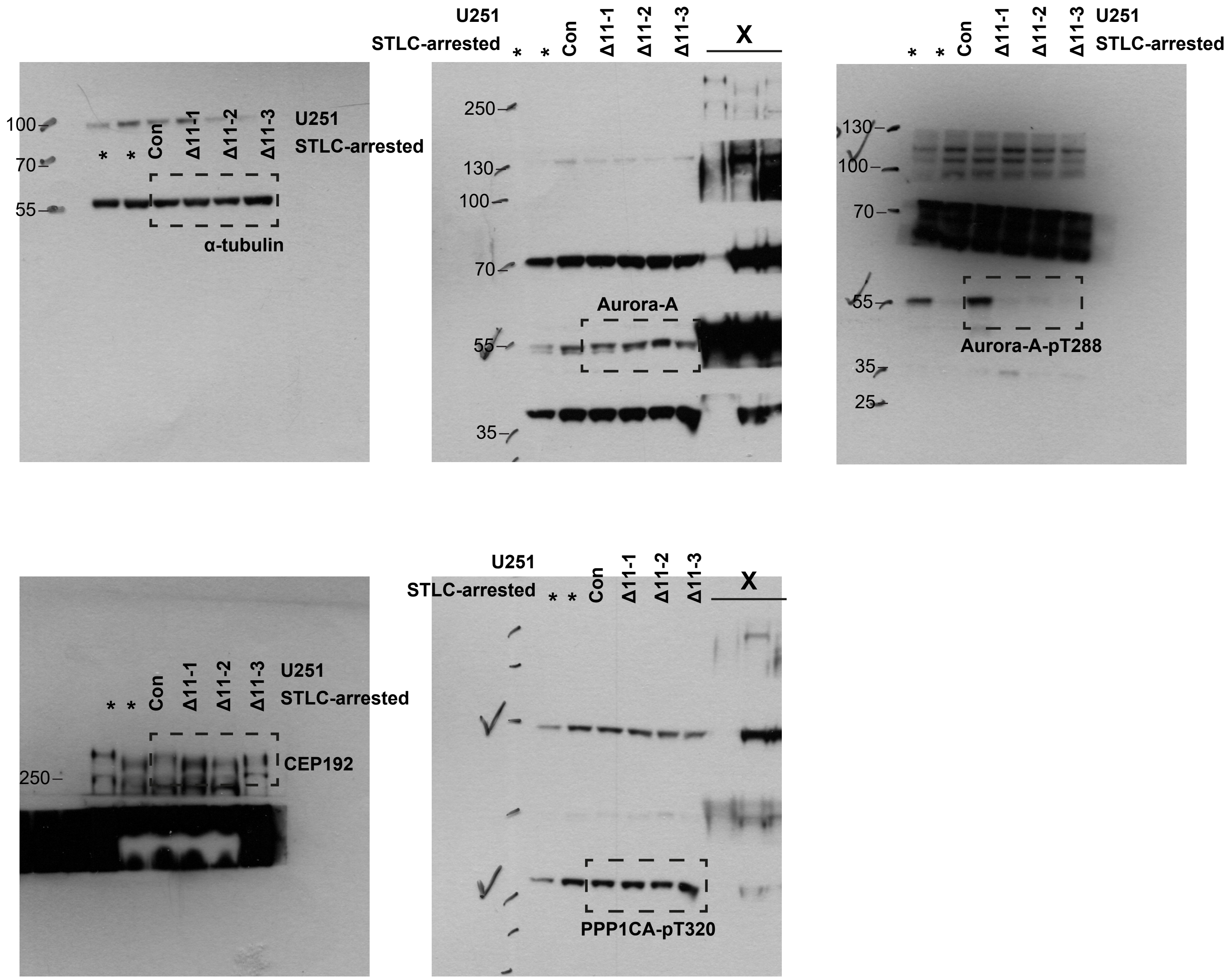

Supplement: Supplementary file 10 — EV and Appendix Figure Source Data [file 44318_2024_240_MOESM10_ESM.zip › Appendix/S7/S7C/Source data_Appendix Figure S7C_Western blots.pdf]
