# Supplementary material for: CEP192 localises mitotic Aurora-A activity by priming its interaction with TPX2
Source: EMBO J. 2024 Sep 26;43(22):5381–420. doi: 10.1038/s44318-024-00240-z (PMC11574021; doi:10.1038/s44318-024-00240-z)
Supplement: Supplementary file 10 — EV and Appendix Figure Source Data [file 44318_2024_240_MOESM10_ESM.zip › Appendix/S8/S8C/Source data_Appendix Figure S8C_western blots.pdf]

Source Data Appendix Figure S8C
S8C - Rotation of crop area was adjusted slightly as required to obtain horizontally aligned bands.

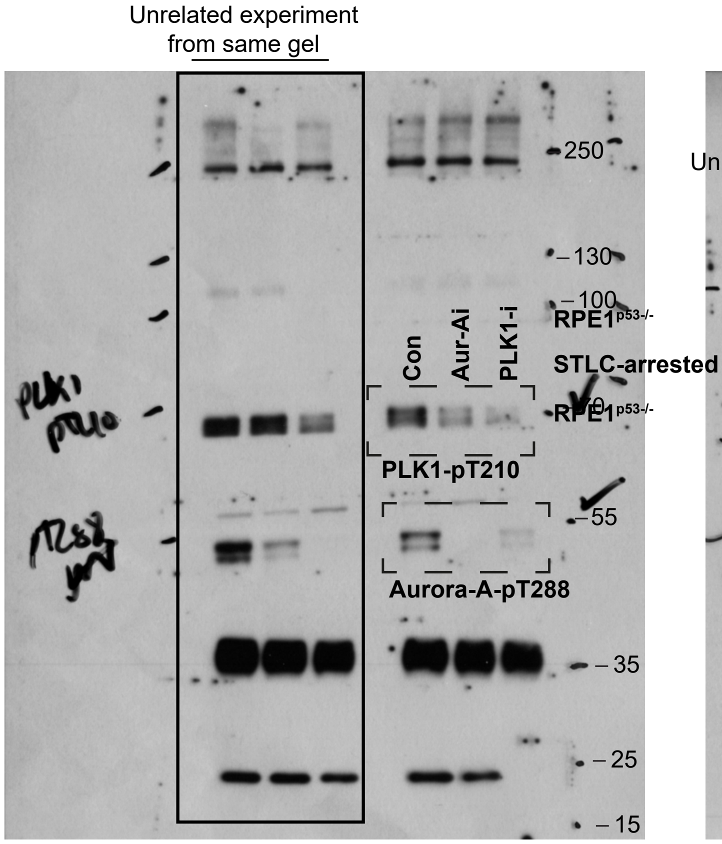

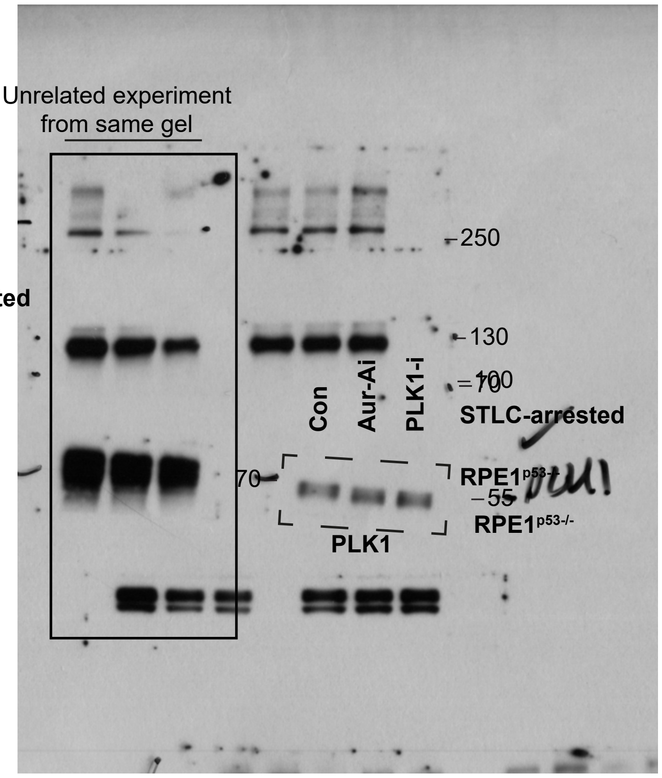

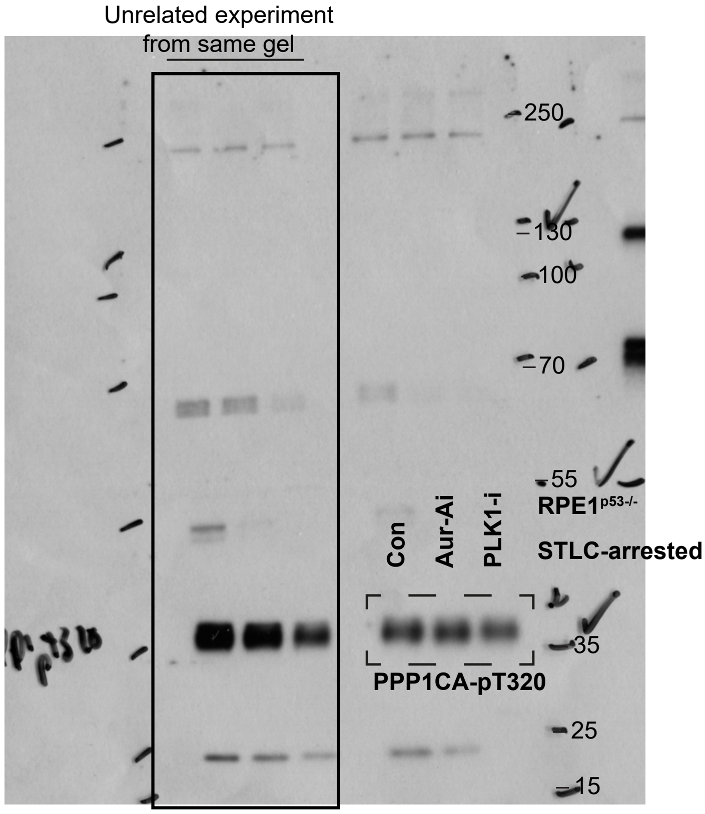

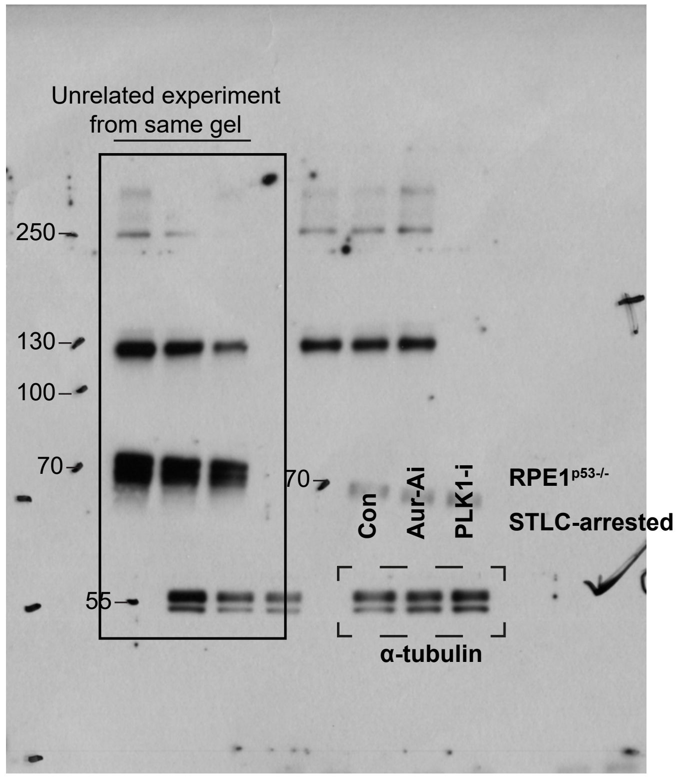

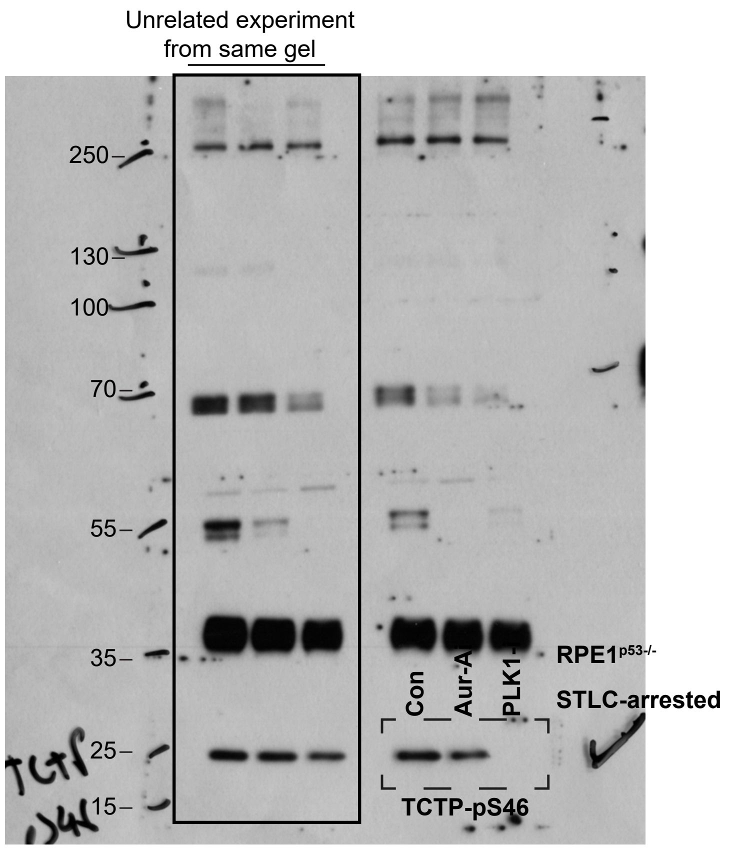
Unrelated experiment
from same gel
250
130
100
70
55
35
25
15
Con
Aur-A
PLK1-i
RPE1p53-/-
STLC-arrested
TCTP-pS46

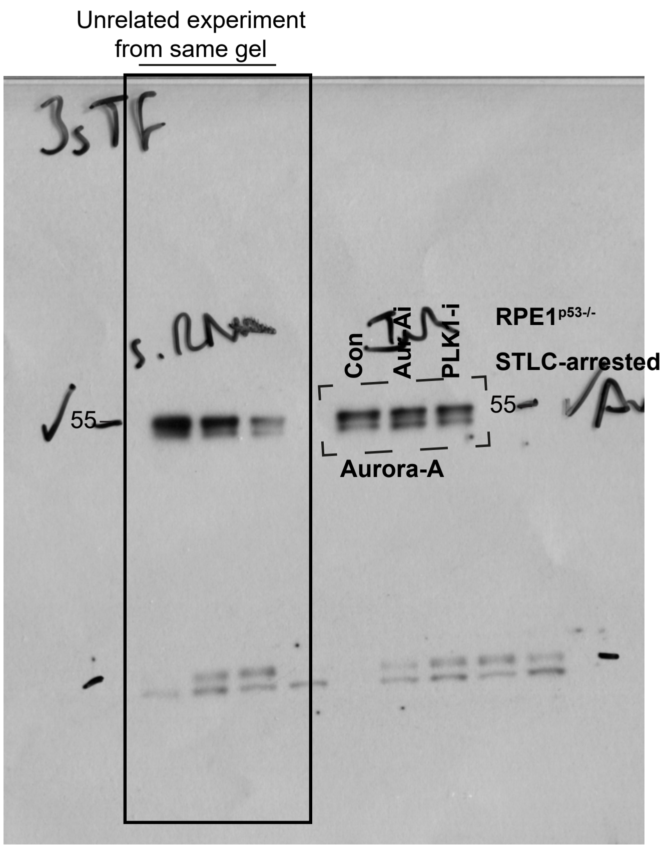
Unrelated experiment
from same gel
55
Con
Aur-Ai
PLK1-i
RPE1p53-/-
STLC-arrested
Aurora-A
55
