# Supplementary material for: CEP192 localises mitotic Aurora-A activity by priming its interaction with TPX2
Source: EMBO J. 2024 Sep 26;43(22):5381–420. doi: 10.1038/s44318-024-00240-z (PMC11574021; doi:10.1038/s44318-024-00240-z)
Supplement: Supplementary file 10 — EV and Appendix Figure Source Data [file 44318_2024_240_MOESM10_ESM.zip › Expanded View/EV1/EV1A/Source data_EV1A_western blots.pdf]

Source Data EV Figure 1

EV1A - Rotation of crop area was adjusted slightly as required to obtain horizontally aligned bands.

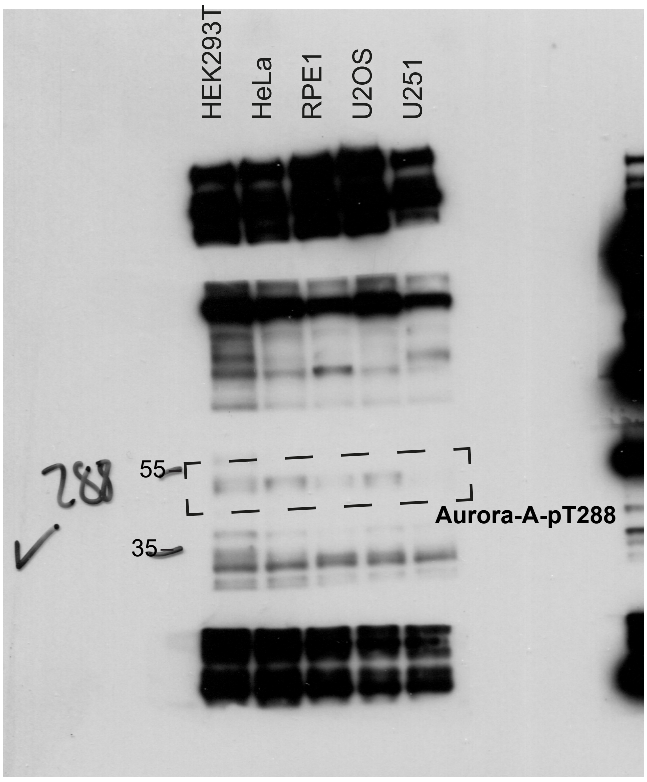

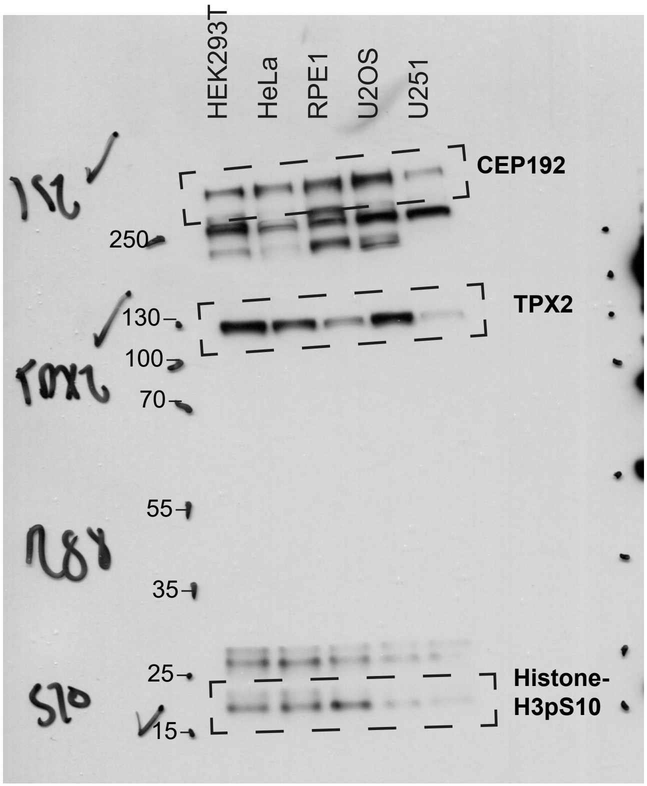

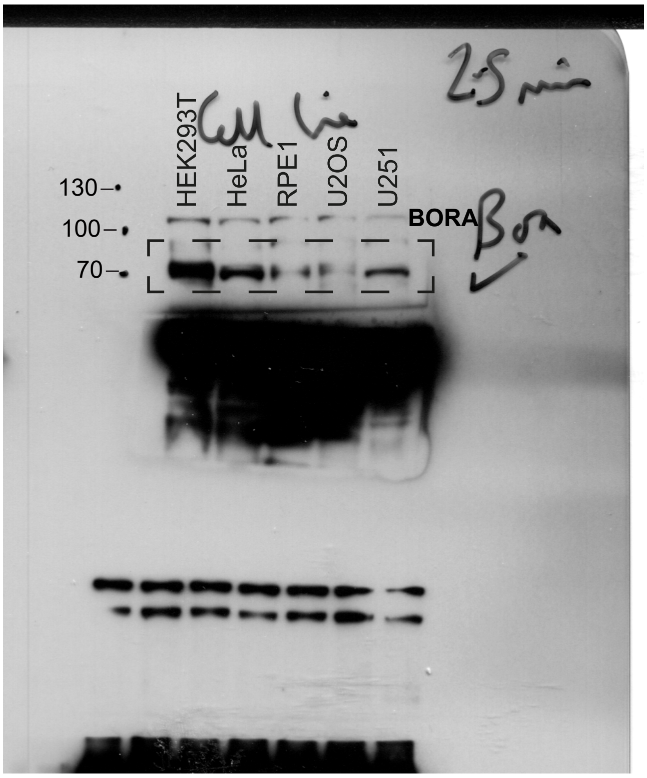

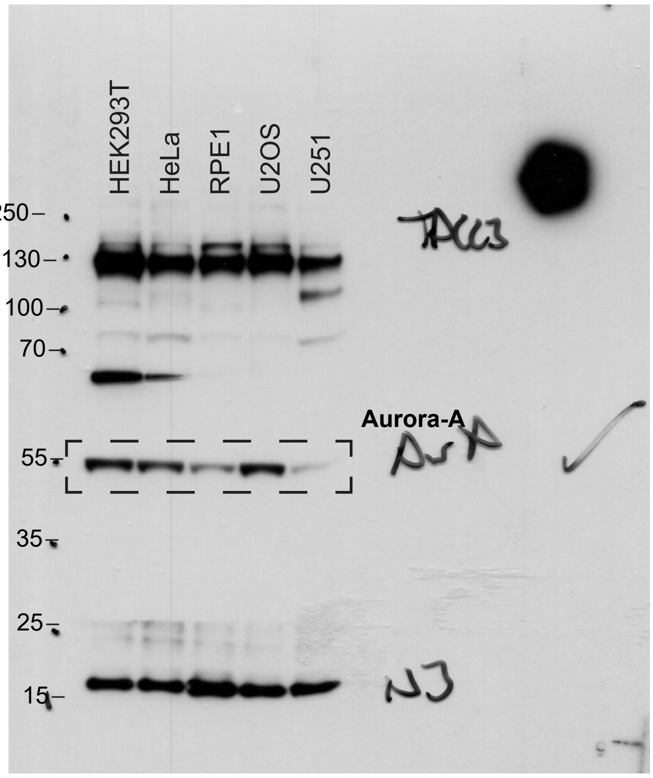

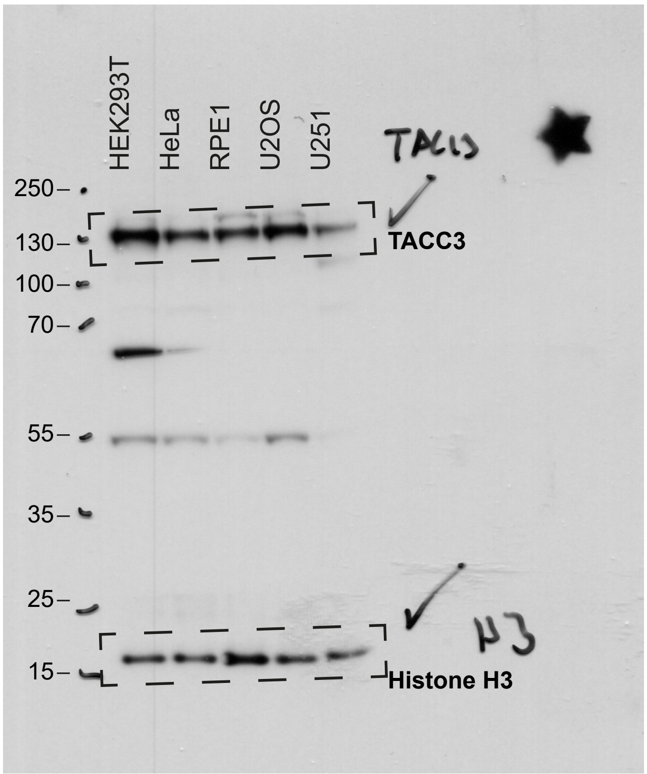
HEK293T
HeLa
RPE1
U2OS
U251
250
130
100
70
55
35
25
15
TACC3
Histone H3

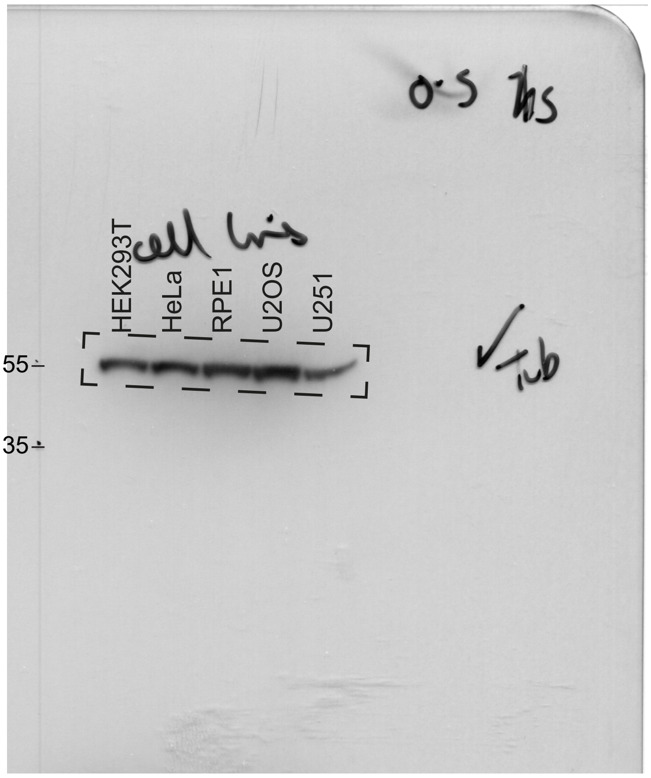
HEK293T
HeLa
RPE1
U2OS
U251
55
35
