# Supplementary material for: CEP192 localises mitotic Aurora-A activity by priming its interaction with TPX2
Source: EMBO J. 2024 Sep 26;43(22):5381–420. doi: 10.1038/s44318-024-00240-z (PMC11574021; doi:10.1038/s44318-024-00240-z)
Supplement: Supplementary file 10 — EV and Appendix Figure Source Data [file 44318_2024_240_MOESM10_ESM.zip › Expanded View/EV1/EV1B/Source data_EV1B_western blots.pdf]

Source Data EV Figure 1

EV1B - Rotation of crop area was adjusted slightly as required to obtain horizontally aligned bands.

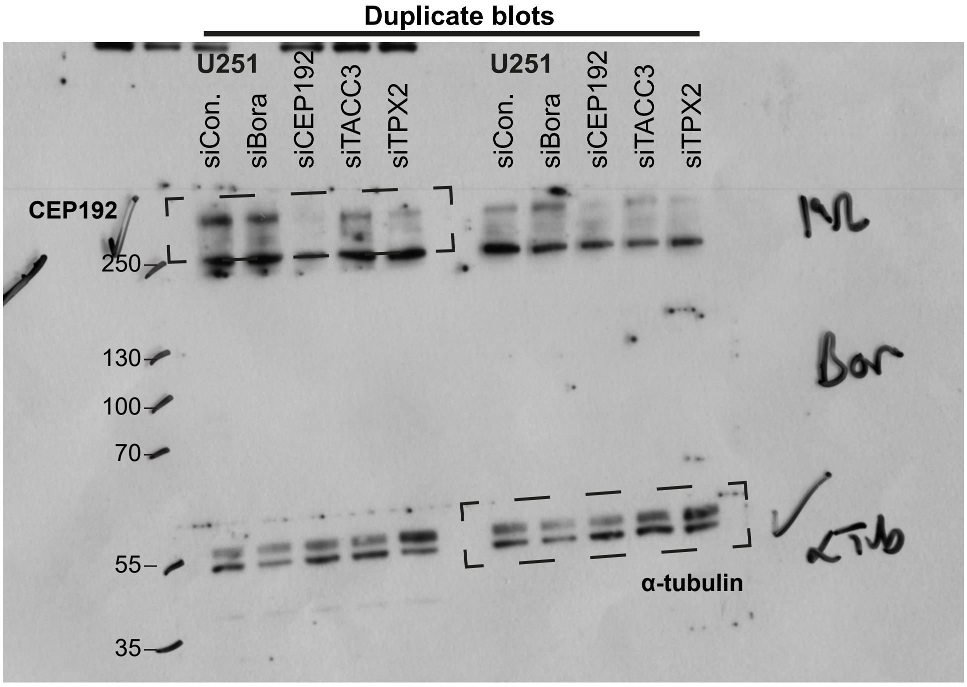

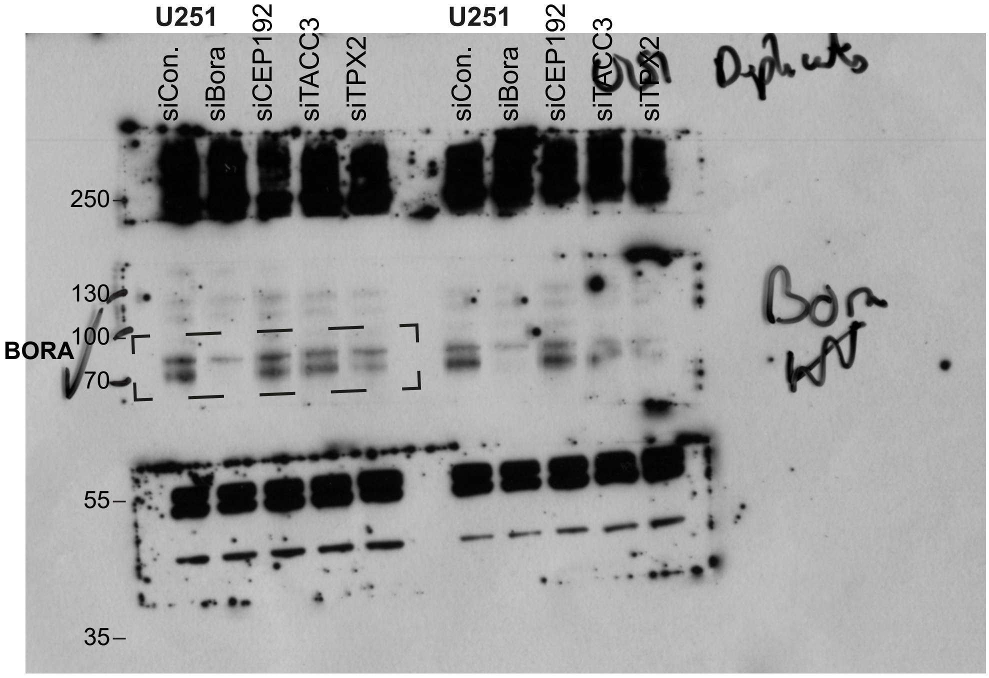

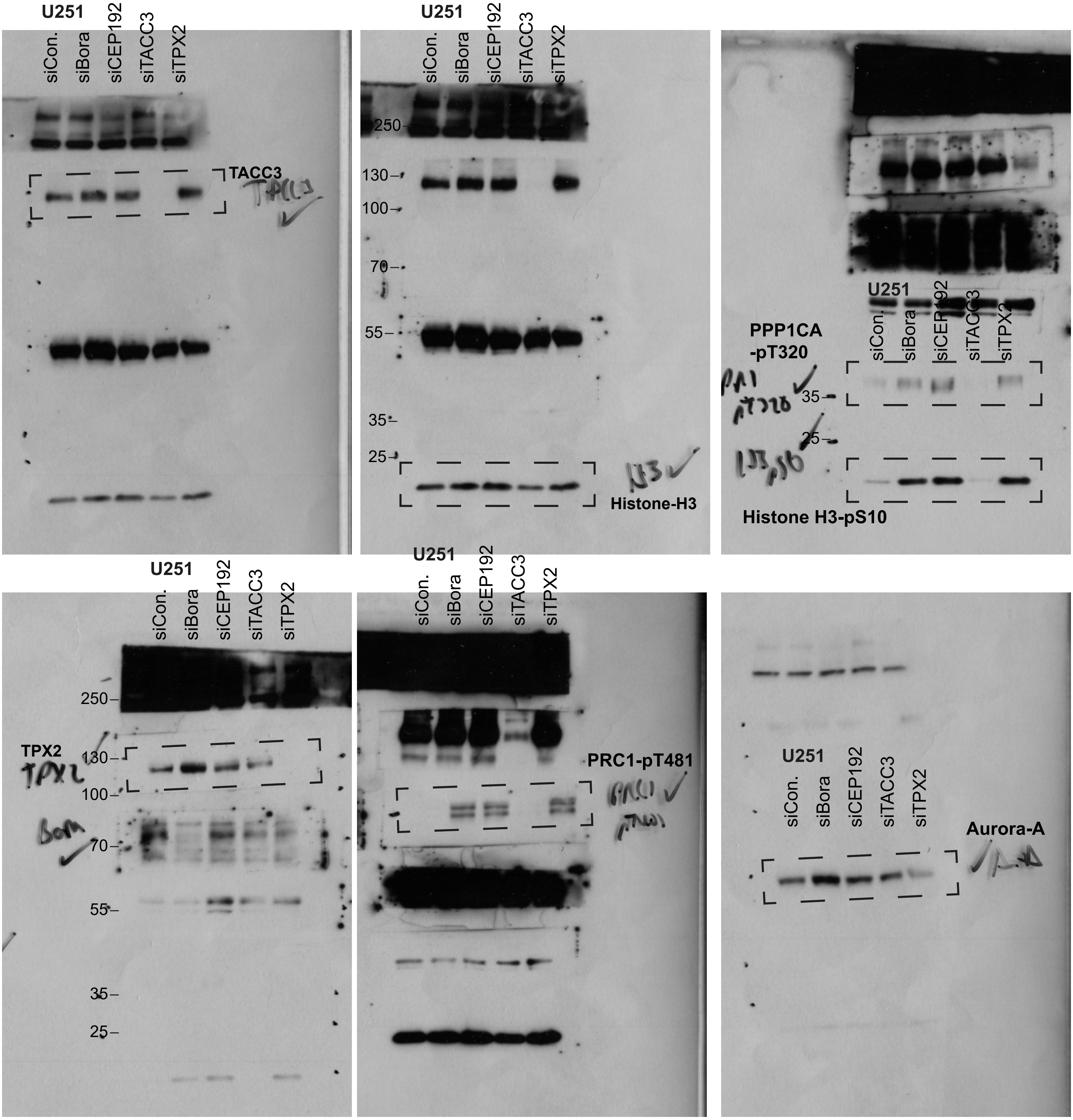
U251
siCon.
siBora
siCEP192
siTACC3
siTPX2
TACC3
250
130
100
70
55
35
25
Histone-H3
PPP1CA
-pT320
Histone H3-pS10
TPX2
PRC1-pT481
Aurora-A
