# Supplementary material for: CEP192 localises mitotic Aurora-A activity by priming its interaction with TPX2
Source: EMBO J. 2024 Sep 26;43(22):5381–420. doi: 10.1038/s44318-024-00240-z (PMC11574021; doi:10.1038/s44318-024-00240-z)
Supplement: Supplementary file 10 — EV and Appendix Figure Source Data [file 44318_2024_240_MOESM10_ESM.zip › Expanded View/EV1/EV1C/Source data_EV1C_western blots.pdf]

Source Data EV Figure 1

EV1C - Rotation of crop area was adjusted slightly as required to obtain horizontally aligned bands.

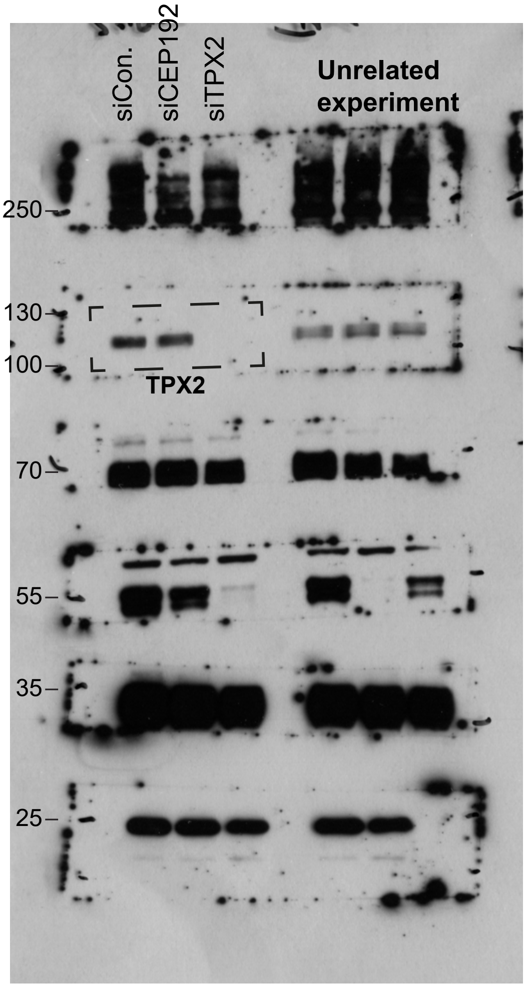

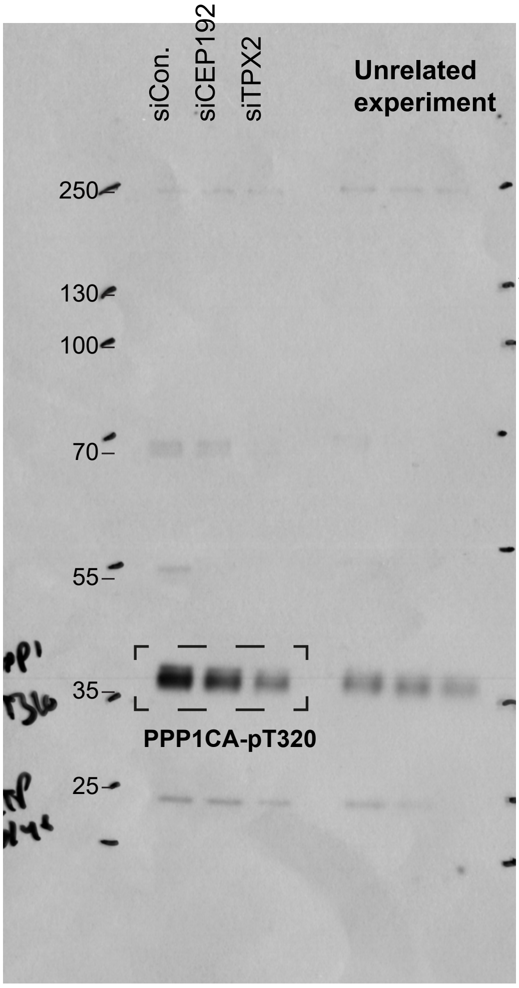

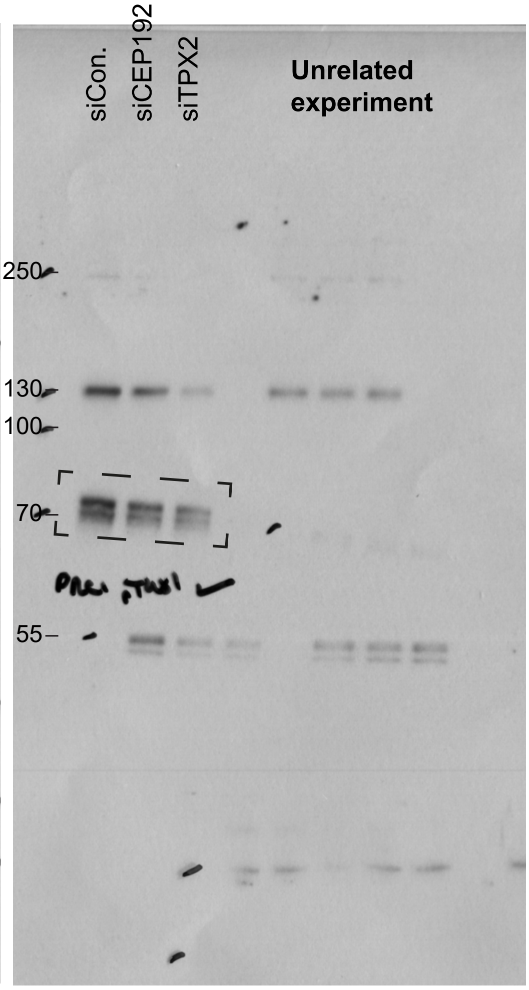

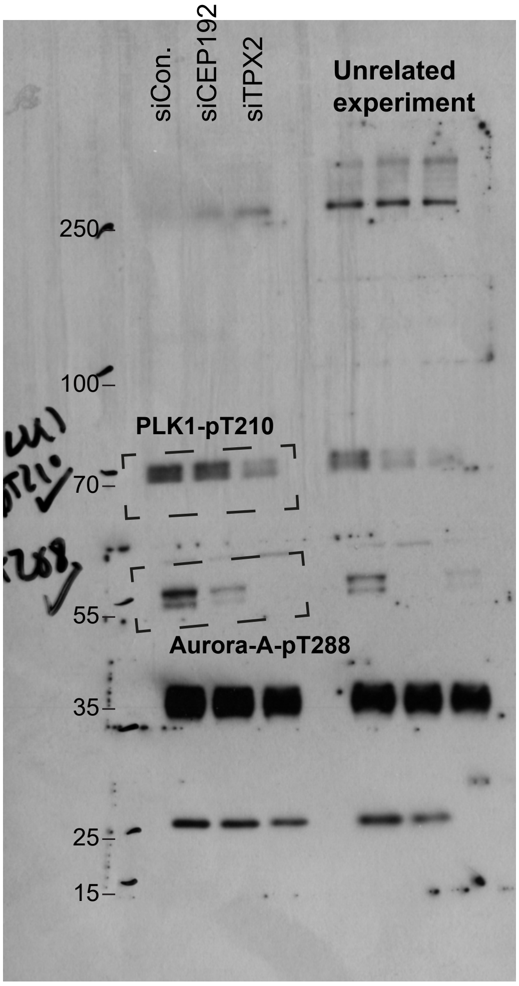

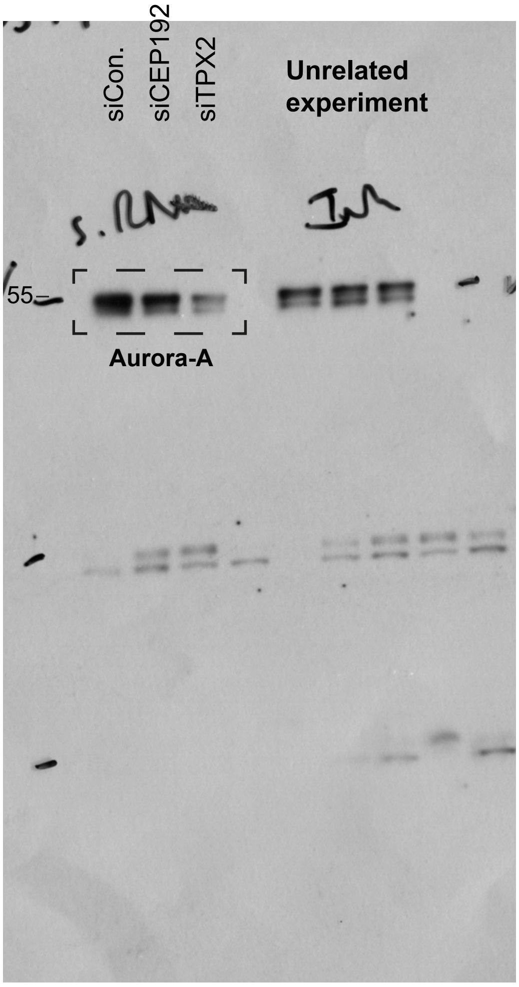

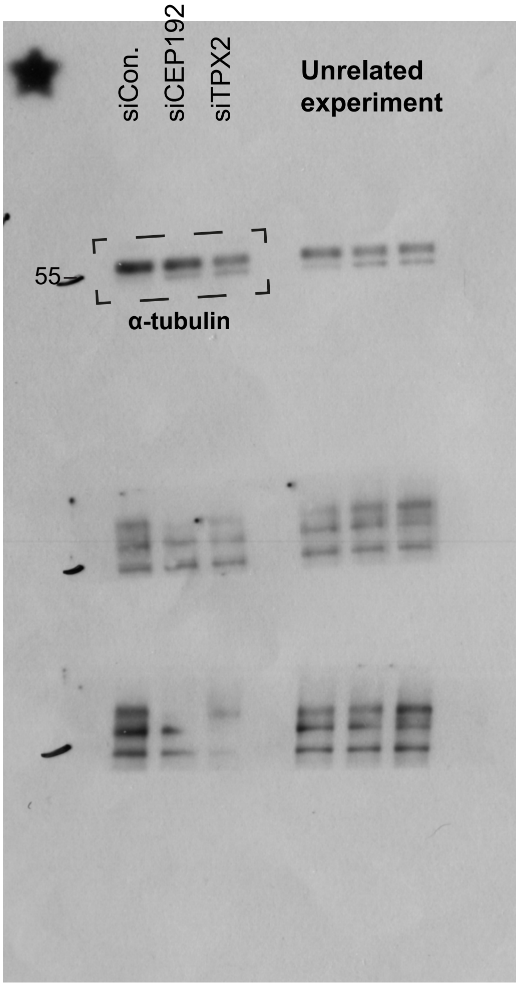

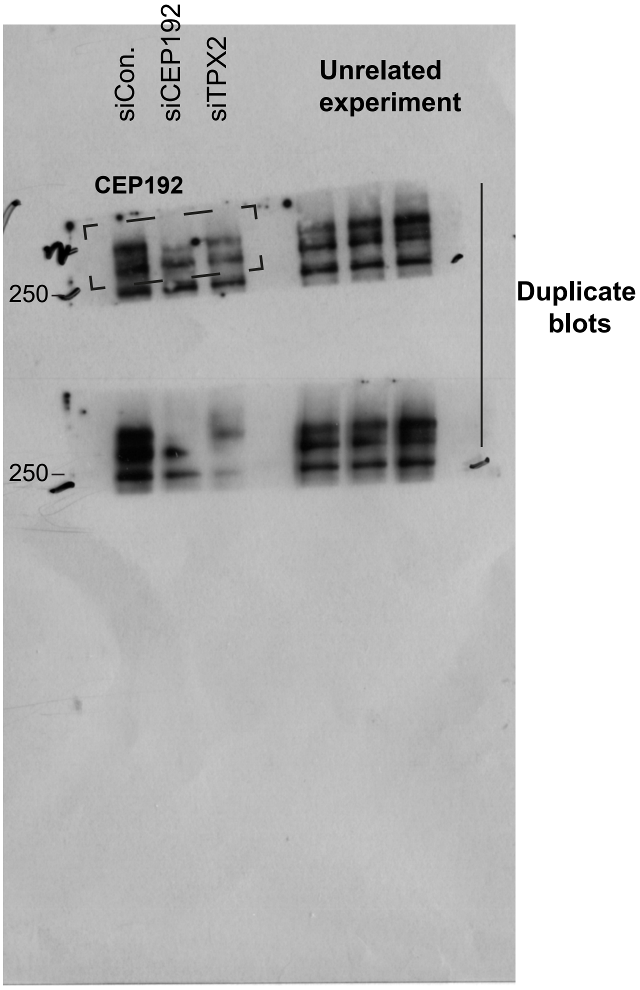
siCon.
siCEP192
siTPX2
Unrelated experiment
CEP192
250
250
Duplicate blots
