# Supplementary figures and images for: CEP192 localises mitotic Aurora-A activity by priming its interaction with TPX2 (part 2 of 2)
Source: EMBO J. 2024 Sep 26;43(22):5381–420. doi: 10.1038/s44318-024-00240-z (PMC11574021; doi:10.1038/s44318-024-00240-z)

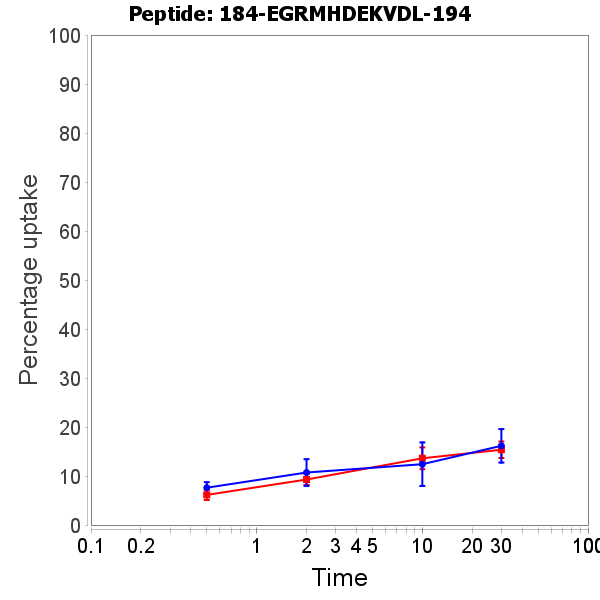

Supplement: Supplementary file 10 — EV and Appendix Figure Source Data [file 44318_2024_240_MOESM10_ESM.zip › Expanded View/EV2/EV2D/Source_Data_Fig_EV2D_HDX_MS_Uptake_Plots_AurA/chart_output184-194.png]

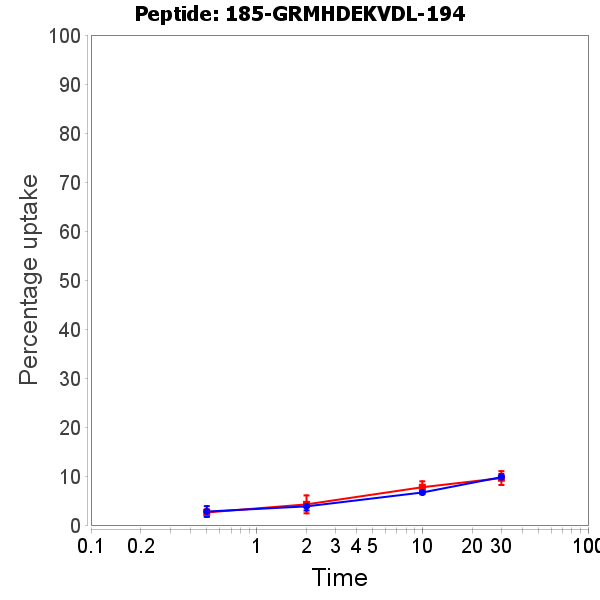

Supplement: Supplementary file 10 — EV and Appendix Figure Source Data [file 44318_2024_240_MOESM10_ESM.zip › Expanded View/EV2/EV2D/Source_Data_Fig_EV2D_HDX_MS_Uptake_Plots_AurA/chart_output185-194.png]

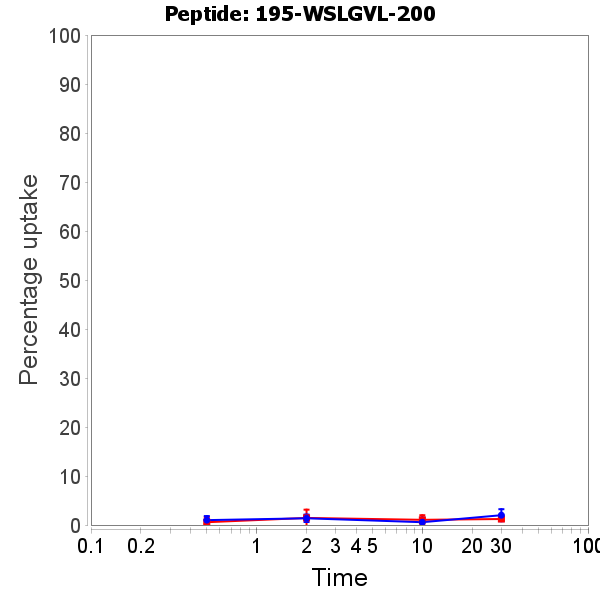

Supplement: Supplementary file 10 — EV and Appendix Figure Source Data [file 44318_2024_240_MOESM10_ESM.zip › Expanded View/EV2/EV2D/Source_Data_Fig_EV2D_HDX_MS_Uptake_Plots_AurA/chart_output195-200.png]

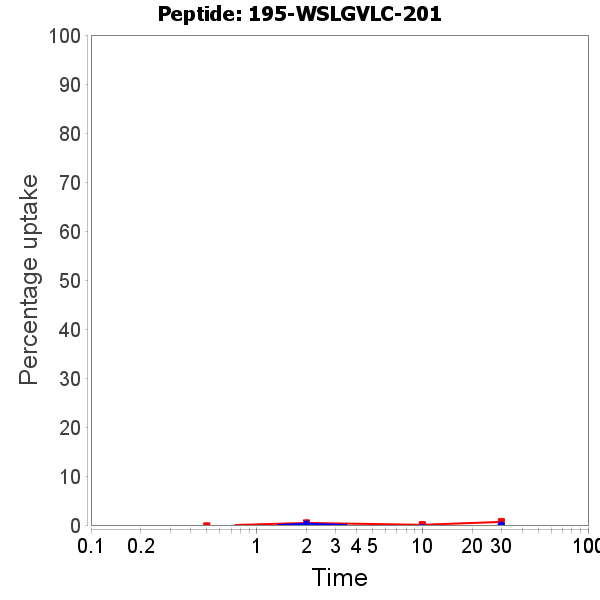

Supplement: Supplementary file 10 — EV and Appendix Figure Source Data [file 44318_2024_240_MOESM10_ESM.zip › Expanded View/EV2/EV2D/Source_Data_Fig_EV2D_HDX_MS_Uptake_Plots_AurA/chart_output195-201.png]

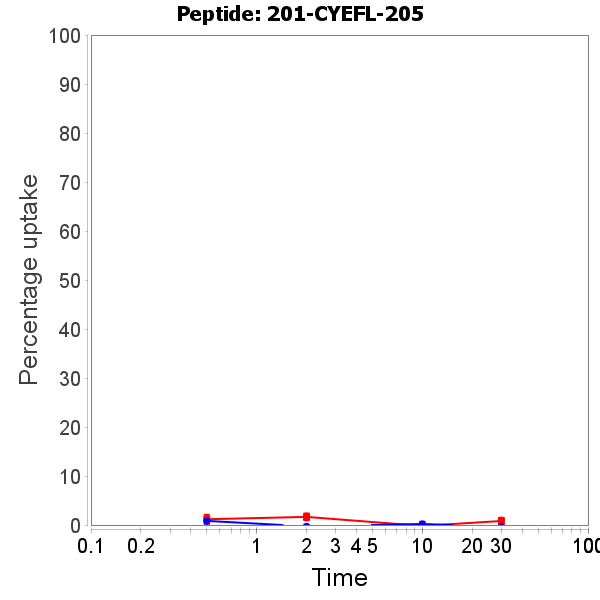

Supplement: Supplementary file 10 — EV and Appendix Figure Source Data [file 44318_2024_240_MOESM10_ESM.zip › Expanded View/EV2/EV2D/Source_Data_Fig_EV2D_HDX_MS_Uptake_Plots_AurA/chart_output201-205.png]

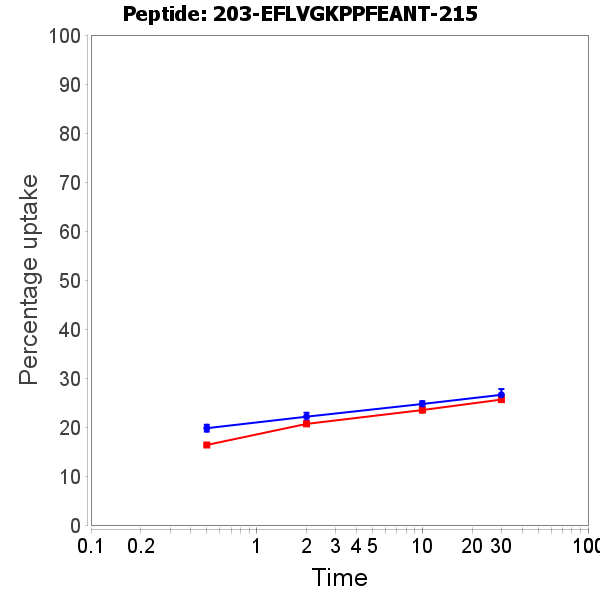

Supplement: Supplementary file 10 — EV and Appendix Figure Source Data [file 44318_2024_240_MOESM10_ESM.zip › Expanded View/EV2/EV2D/Source_Data_Fig_EV2D_HDX_MS_Uptake_Plots_AurA/chart_output203-215.png]

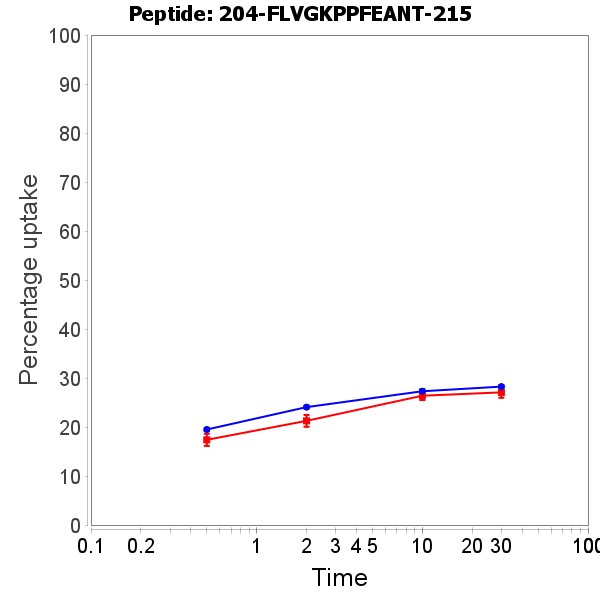

Supplement: Supplementary file 10 — EV and Appendix Figure Source Data [file 44318_2024_240_MOESM10_ESM.zip › Expanded View/EV2/EV2D/Source_Data_Fig_EV2D_HDX_MS_Uptake_Plots_AurA/chart_output204-215.png]

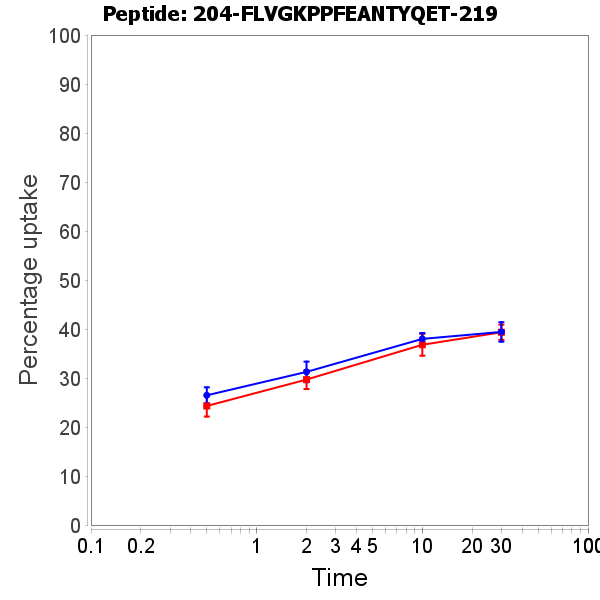

Supplement: Supplementary file 10 — EV and Appendix Figure Source Data [file 44318_2024_240_MOESM10_ESM.zip › Expanded View/EV2/EV2D/Source_Data_Fig_EV2D_HDX_MS_Uptake_Plots_AurA/chart_output204-219.png]

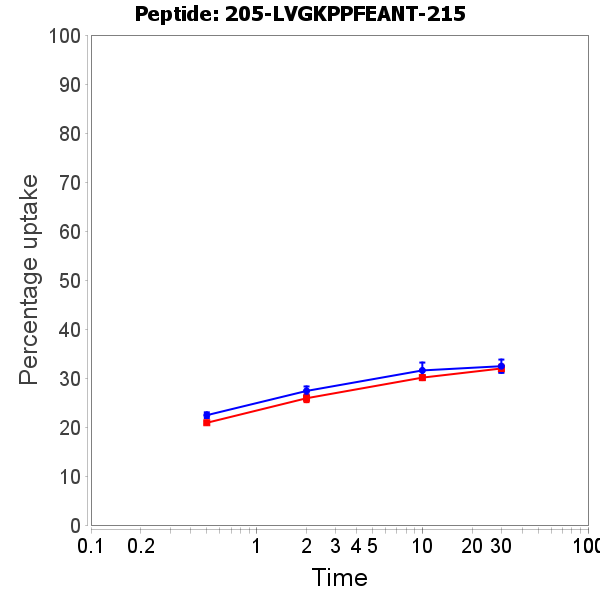

Supplement: Supplementary file 10 — EV and Appendix Figure Source Data [file 44318_2024_240_MOESM10_ESM.zip › Expanded View/EV2/EV2D/Source_Data_Fig_EV2D_HDX_MS_Uptake_Plots_AurA/chart_output205-215.png]

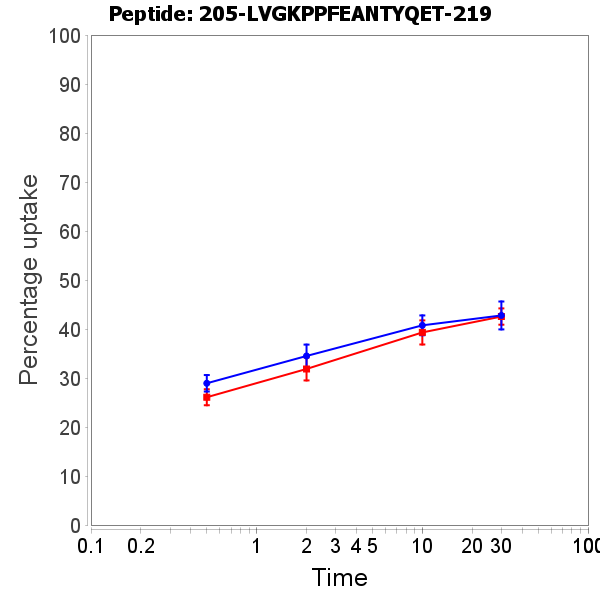

Supplement: Supplementary file 10 — EV and Appendix Figure Source Data [file 44318_2024_240_MOESM10_ESM.zip › Expanded View/EV2/EV2D/Source_Data_Fig_EV2D_HDX_MS_Uptake_Plots_AurA/chart_output205-219.png]

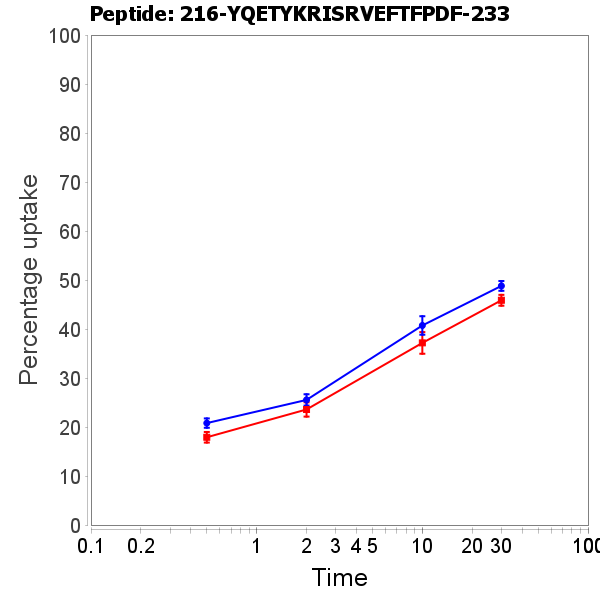

Supplement: Supplementary file 10 — EV and Appendix Figure Source Data [file 44318_2024_240_MOESM10_ESM.zip › Expanded View/EV2/EV2D/Source_Data_Fig_EV2D_HDX_MS_Uptake_Plots_AurA/chart_output216-233.png]

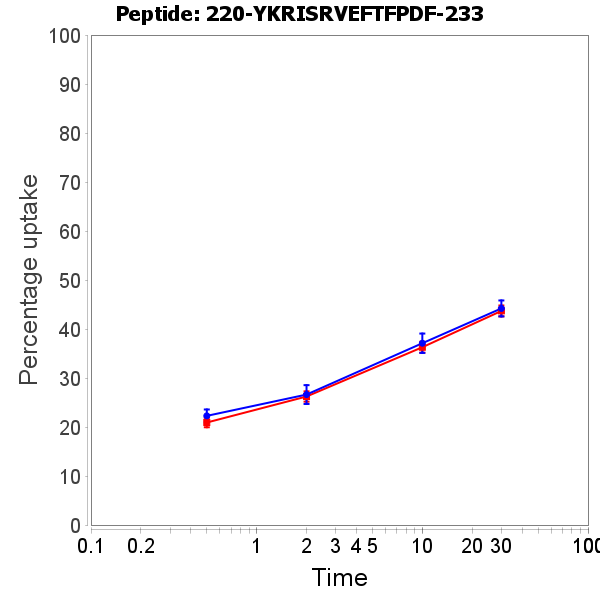

Supplement: Supplementary file 10 — EV and Appendix Figure Source Data [file 44318_2024_240_MOESM10_ESM.zip › Expanded View/EV2/EV2D/Source_Data_Fig_EV2D_HDX_MS_Uptake_Plots_AurA/chart_output220-233.png]

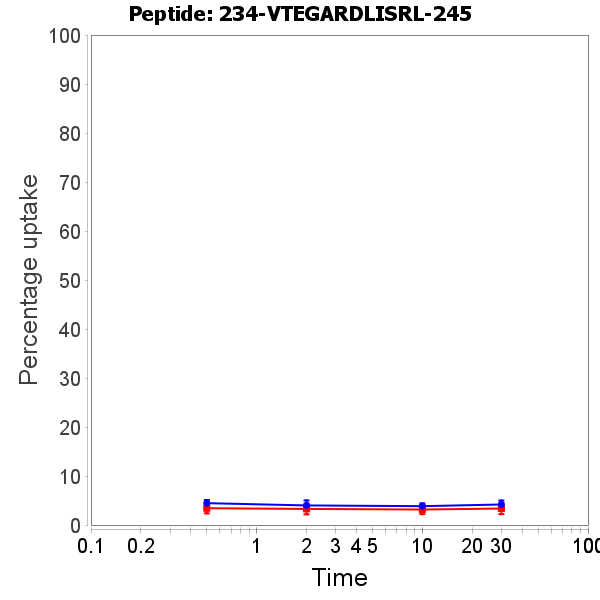

Supplement: Supplementary file 10 — EV and Appendix Figure Source Data [file 44318_2024_240_MOESM10_ESM.zip › Expanded View/EV2/EV2D/Source_Data_Fig_EV2D_HDX_MS_Uptake_Plots_AurA/chart_output234-245.png]

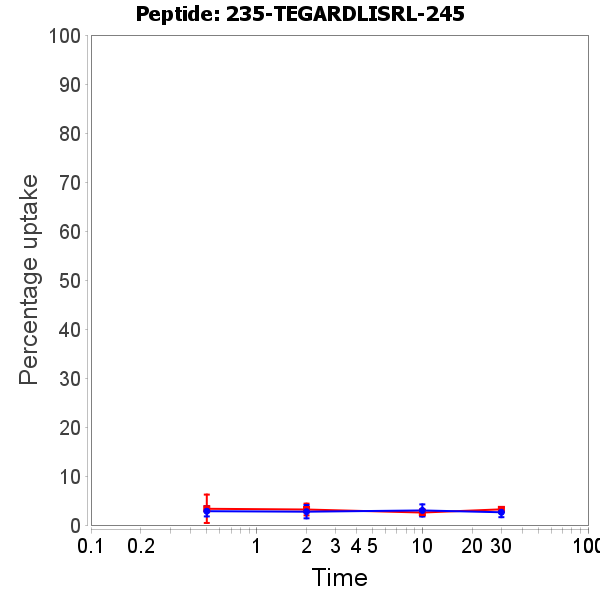

Supplement: Supplementary file 10 — EV and Appendix Figure Source Data [file 44318_2024_240_MOESM10_ESM.zip › Expanded View/EV2/EV2D/Source_Data_Fig_EV2D_HDX_MS_Uptake_Plots_AurA/chart_output235-245.png]

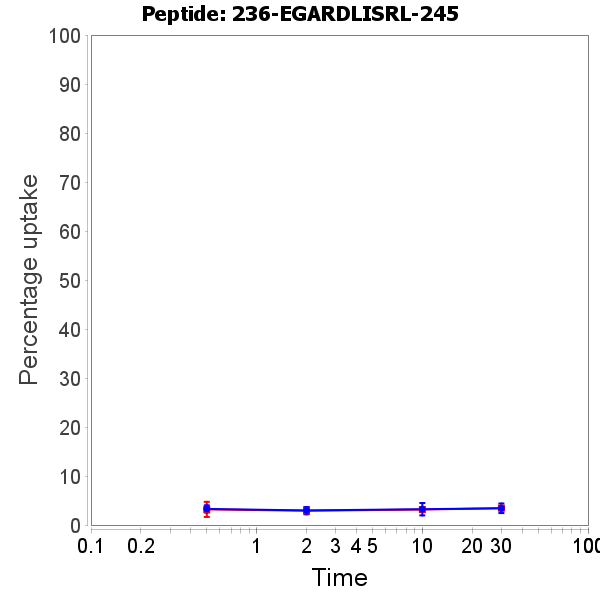

Supplement: Supplementary file 10 — EV and Appendix Figure Source Data [file 44318_2024_240_MOESM10_ESM.zip › Expanded View/EV2/EV2D/Source_Data_Fig_EV2D_HDX_MS_Uptake_Plots_AurA/chart_output236-245.png]

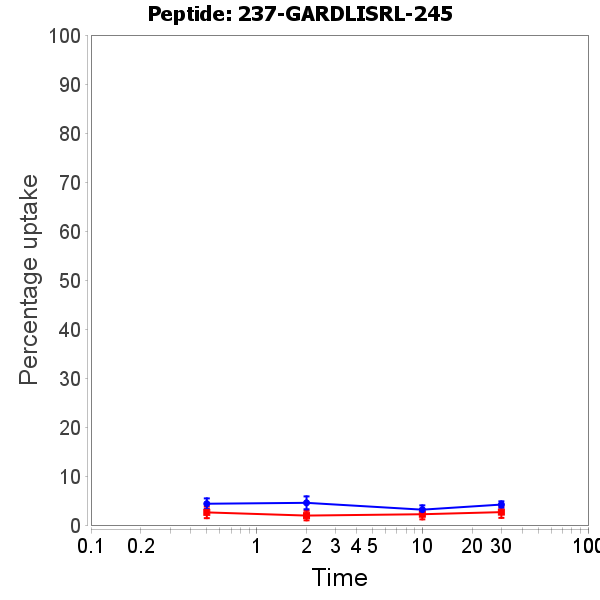

Supplement: Supplementary file 10 — EV and Appendix Figure Source Data [file 44318_2024_240_MOESM10_ESM.zip › Expanded View/EV2/EV2D/Source_Data_Fig_EV2D_HDX_MS_Uptake_Plots_AurA/chart_output237-245.png]

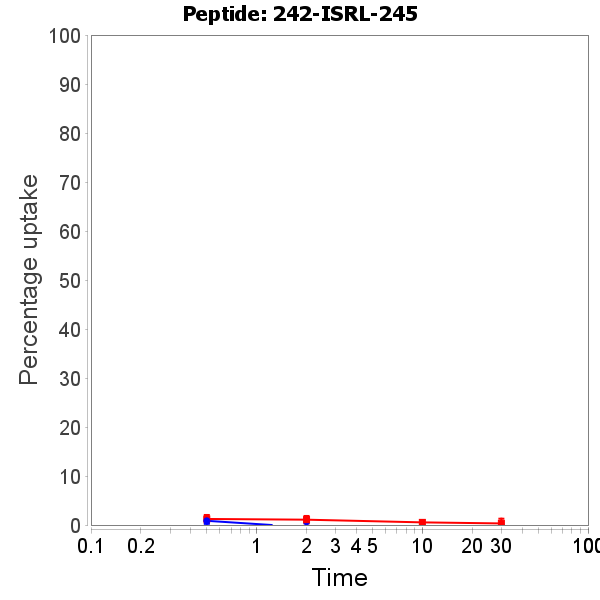

Supplement: Supplementary file 10 — EV and Appendix Figure Source Data [file 44318_2024_240_MOESM10_ESM.zip › Expanded View/EV2/EV2D/Source_Data_Fig_EV2D_HDX_MS_Uptake_Plots_AurA/chart_output242-245.png]

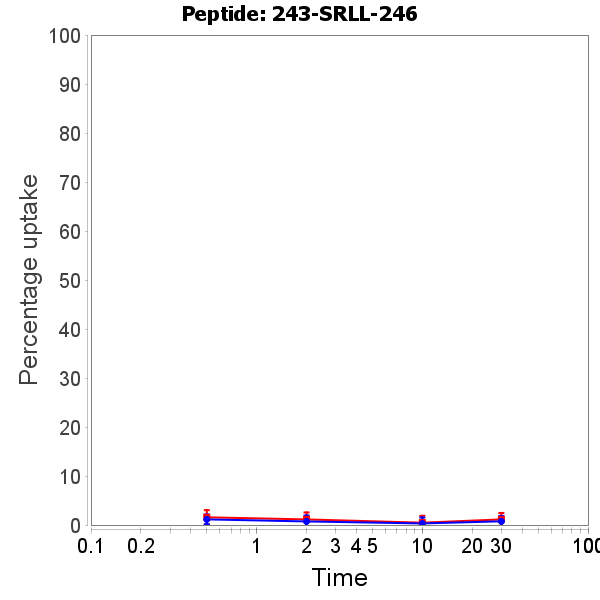

Supplement: Supplementary file 10 — EV and Appendix Figure Source Data [file 44318_2024_240_MOESM10_ESM.zip › Expanded View/EV2/EV2D/Source_Data_Fig_EV2D_HDX_MS_Uptake_Plots_AurA/chart_output243-246.png]

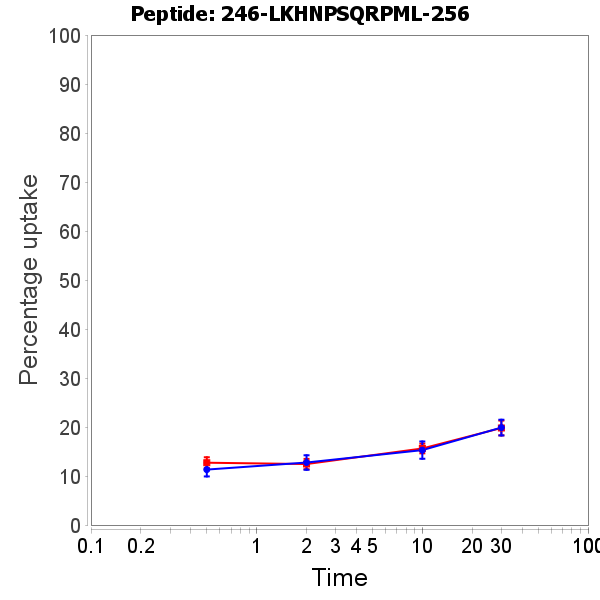

Supplement: Supplementary file 10 — EV and Appendix Figure Source Data [file 44318_2024_240_MOESM10_ESM.zip › Expanded View/EV2/EV2D/Source_Data_Fig_EV2D_HDX_MS_Uptake_Plots_AurA/chart_output246-256.png]

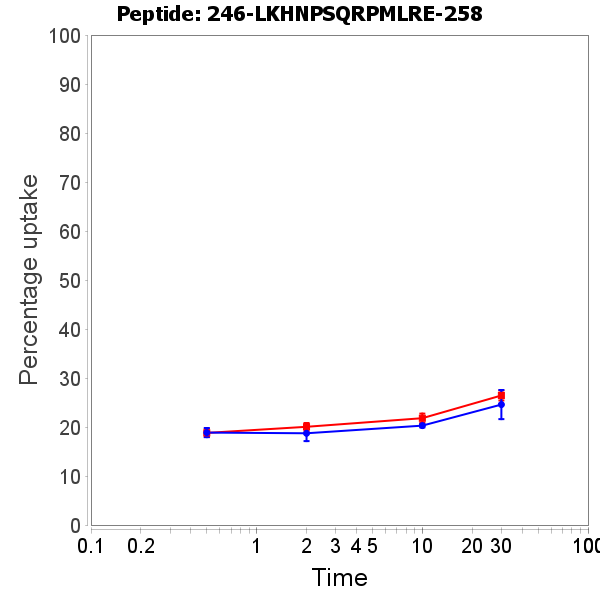

Supplement: Supplementary file 10 — EV and Appendix Figure Source Data [file 44318_2024_240_MOESM10_ESM.zip › Expanded View/EV2/EV2D/Source_Data_Fig_EV2D_HDX_MS_Uptake_Plots_AurA/chart_output246-258.png]

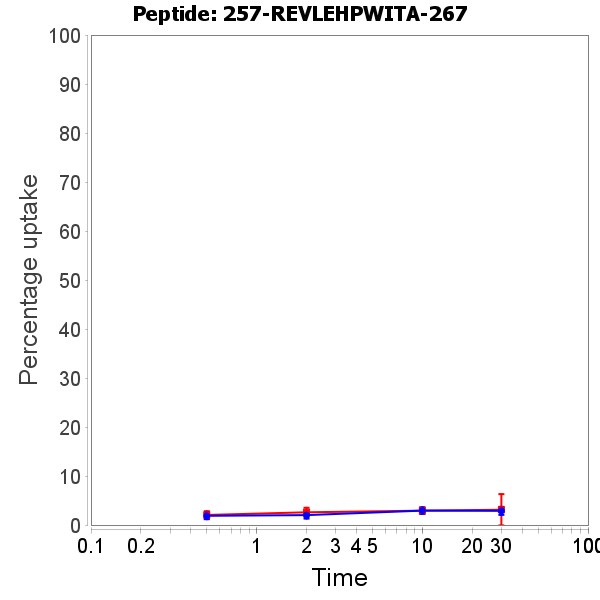

Supplement: Supplementary file 10 — EV and Appendix Figure Source Data [file 44318_2024_240_MOESM10_ESM.zip › Expanded View/EV2/EV2D/Source_Data_Fig_EV2D_HDX_MS_Uptake_Plots_AurA/chart_output257-267.png]

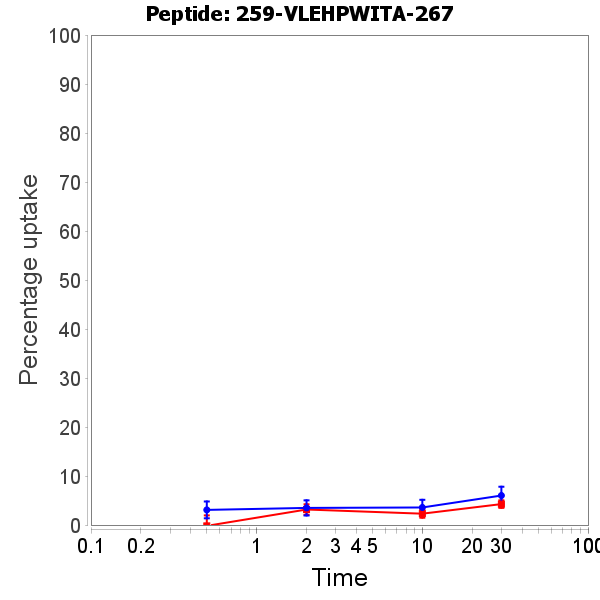

Supplement: Supplementary file 10 — EV and Appendix Figure Source Data [file 44318_2024_240_MOESM10_ESM.zip › Expanded View/EV2/EV2D/Source_Data_Fig_EV2D_HDX_MS_Uptake_Plots_AurA/chart_output259-267.png]

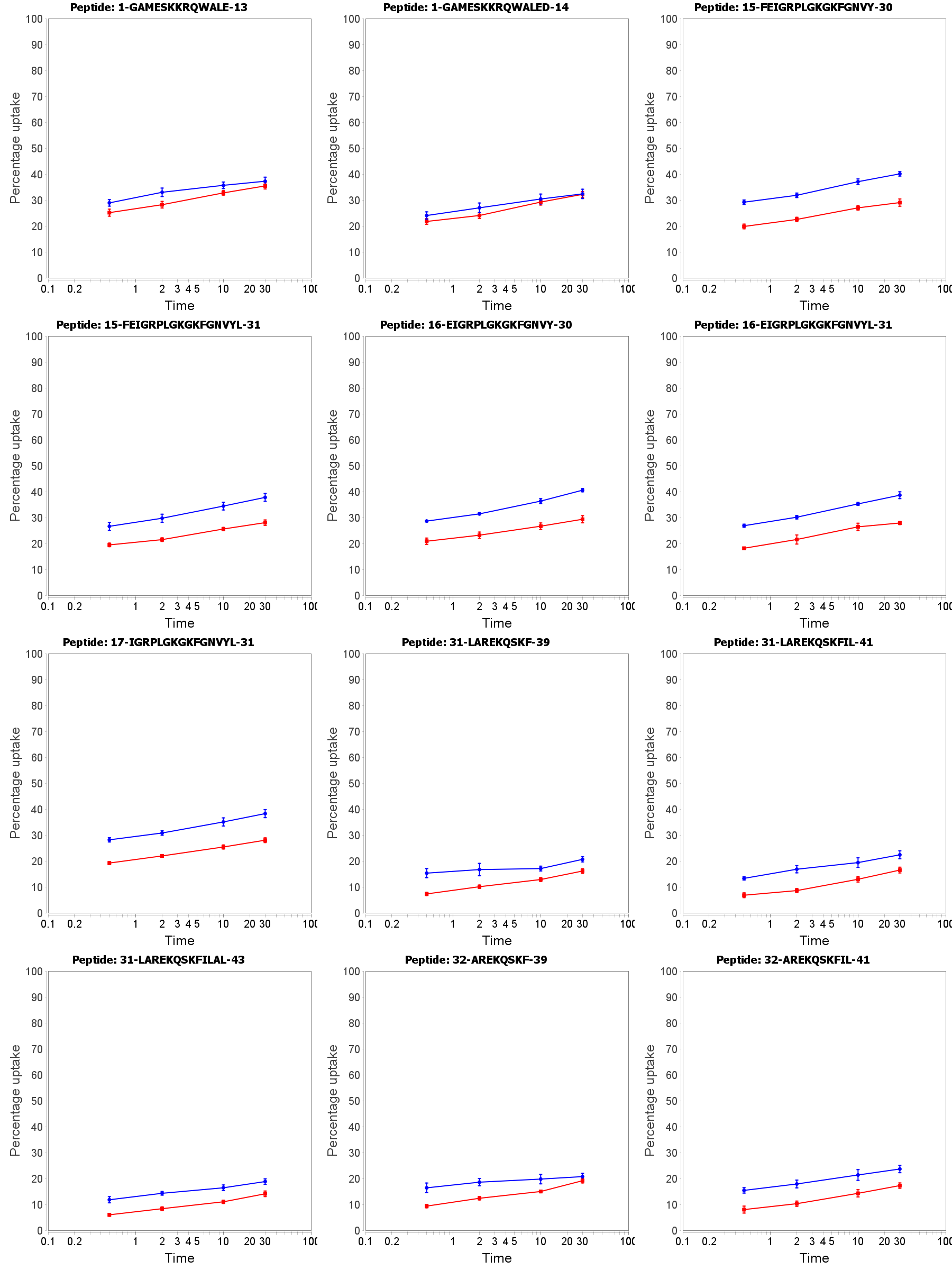

Supplement: Supplementary file 10 — EV and Appendix Figure Source Data [file 44318_2024_240_MOESM10_ESM.zip › Expanded View/EV2/EV2D/Source_Data_Fig_EV2D_HDX_MS_Uptake_Plots_AurA/uptake_1.png]

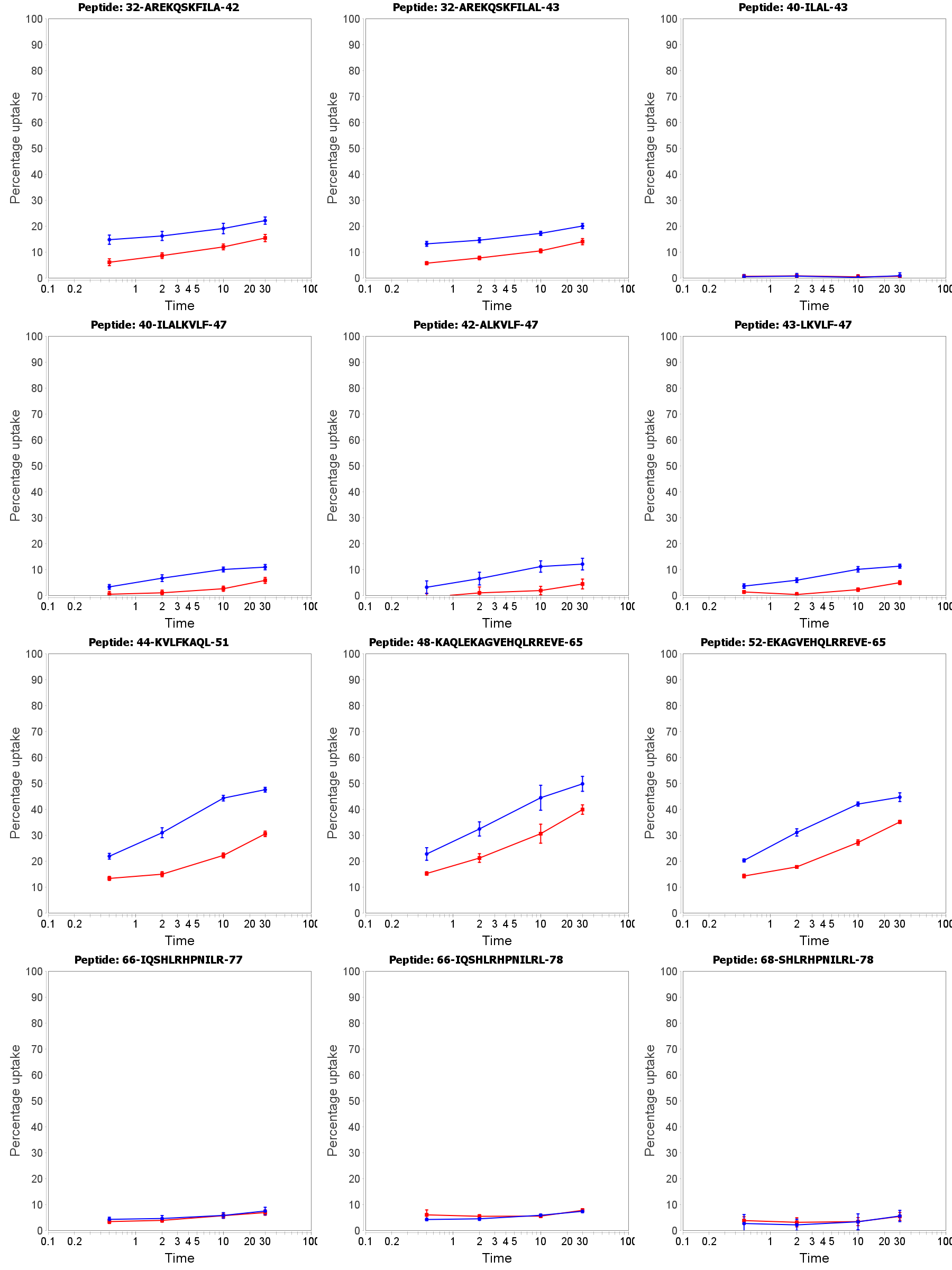

Supplement: Supplementary file 10 — EV and Appendix Figure Source Data [file 44318_2024_240_MOESM10_ESM.zip › Expanded View/EV2/EV2D/Source_Data_Fig_EV2D_HDX_MS_Uptake_Plots_AurA/uptake_2.png]

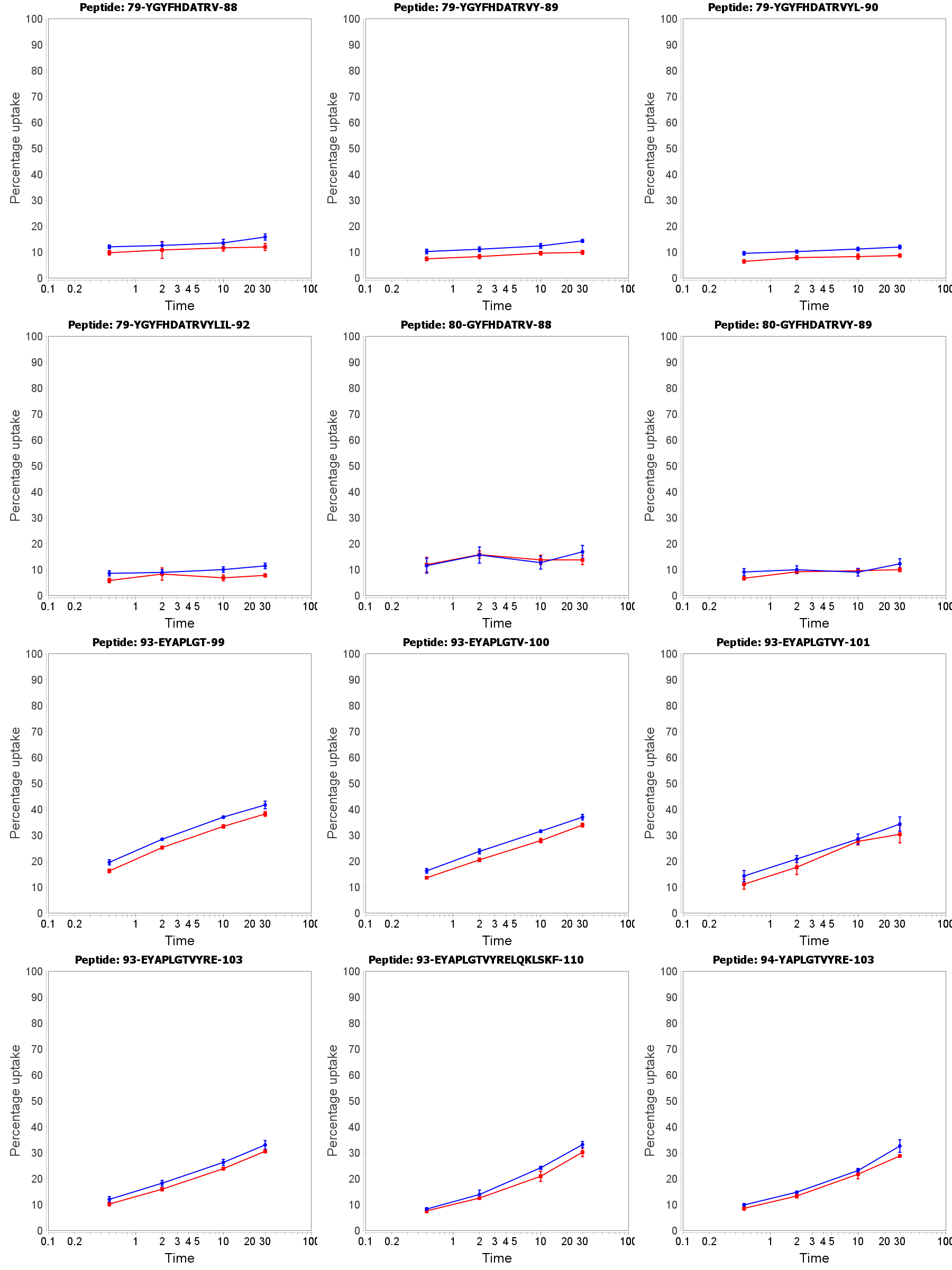

Supplement: Supplementary file 10 — EV and Appendix Figure Source Data [file 44318_2024_240_MOESM10_ESM.zip › Expanded View/EV2/EV2D/Source_Data_Fig_EV2D_HDX_MS_Uptake_Plots_AurA/uptake_3.png]

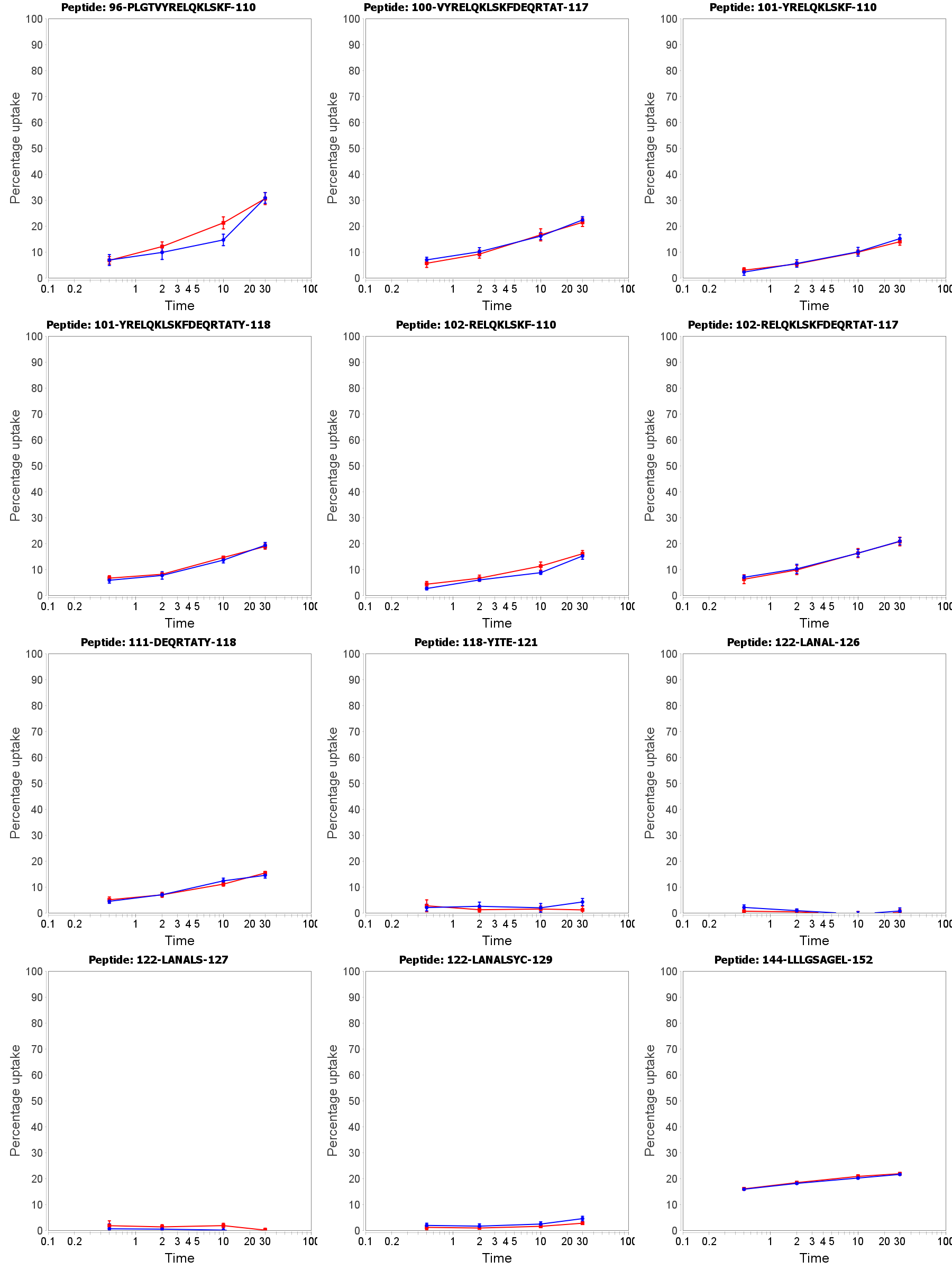

Supplement: Supplementary file 10 — EV and Appendix Figure Source Data [file 44318_2024_240_MOESM10_ESM.zip › Expanded View/EV2/EV2D/Source_Data_Fig_EV2D_HDX_MS_Uptake_Plots_AurA/uptake_4.png]

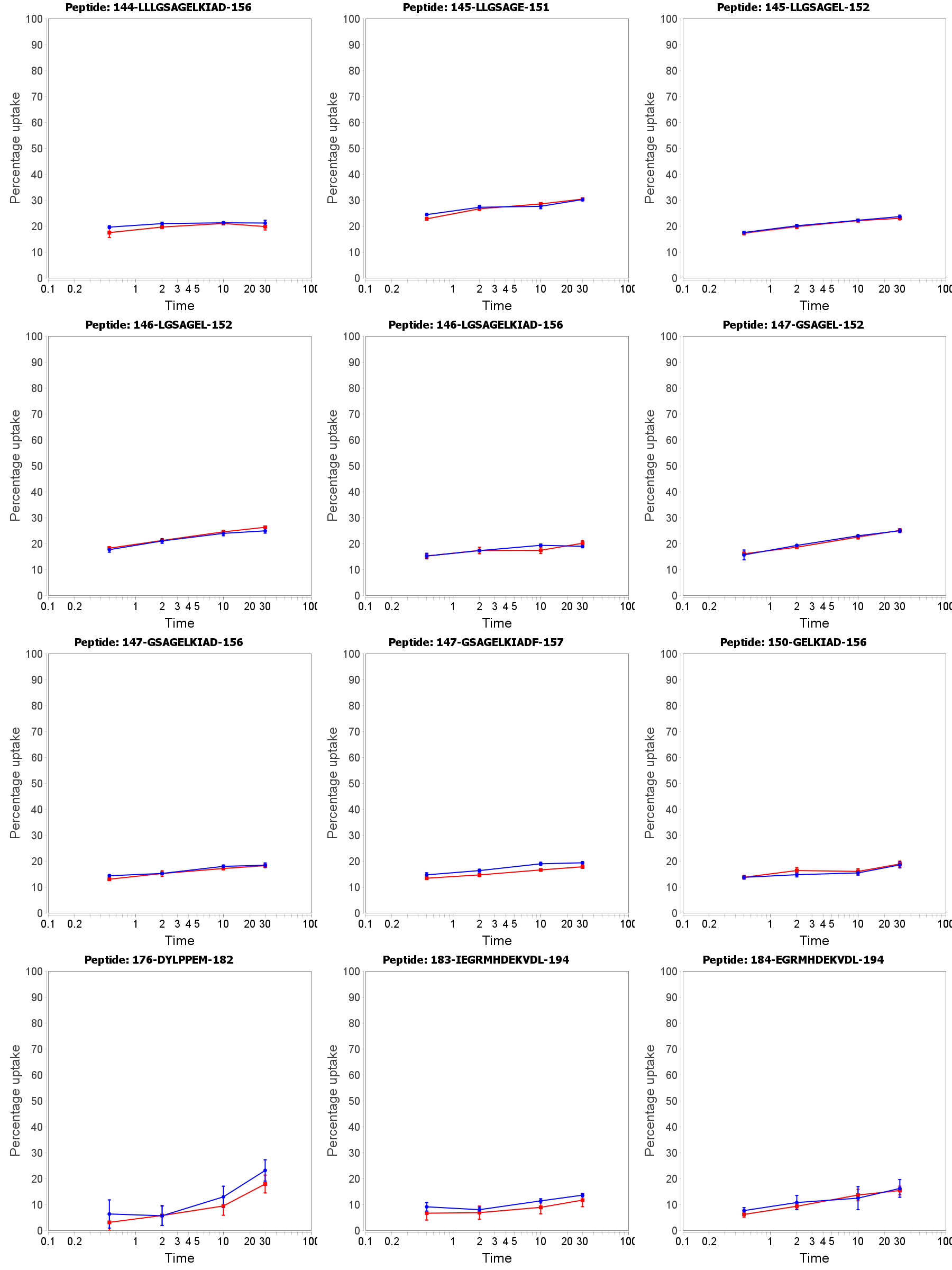

Supplement: Supplementary file 10 — EV and Appendix Figure Source Data [file 44318_2024_240_MOESM10_ESM.zip › Expanded View/EV2/EV2D/Source_Data_Fig_EV2D_HDX_MS_Uptake_Plots_AurA/uptake_5.png]

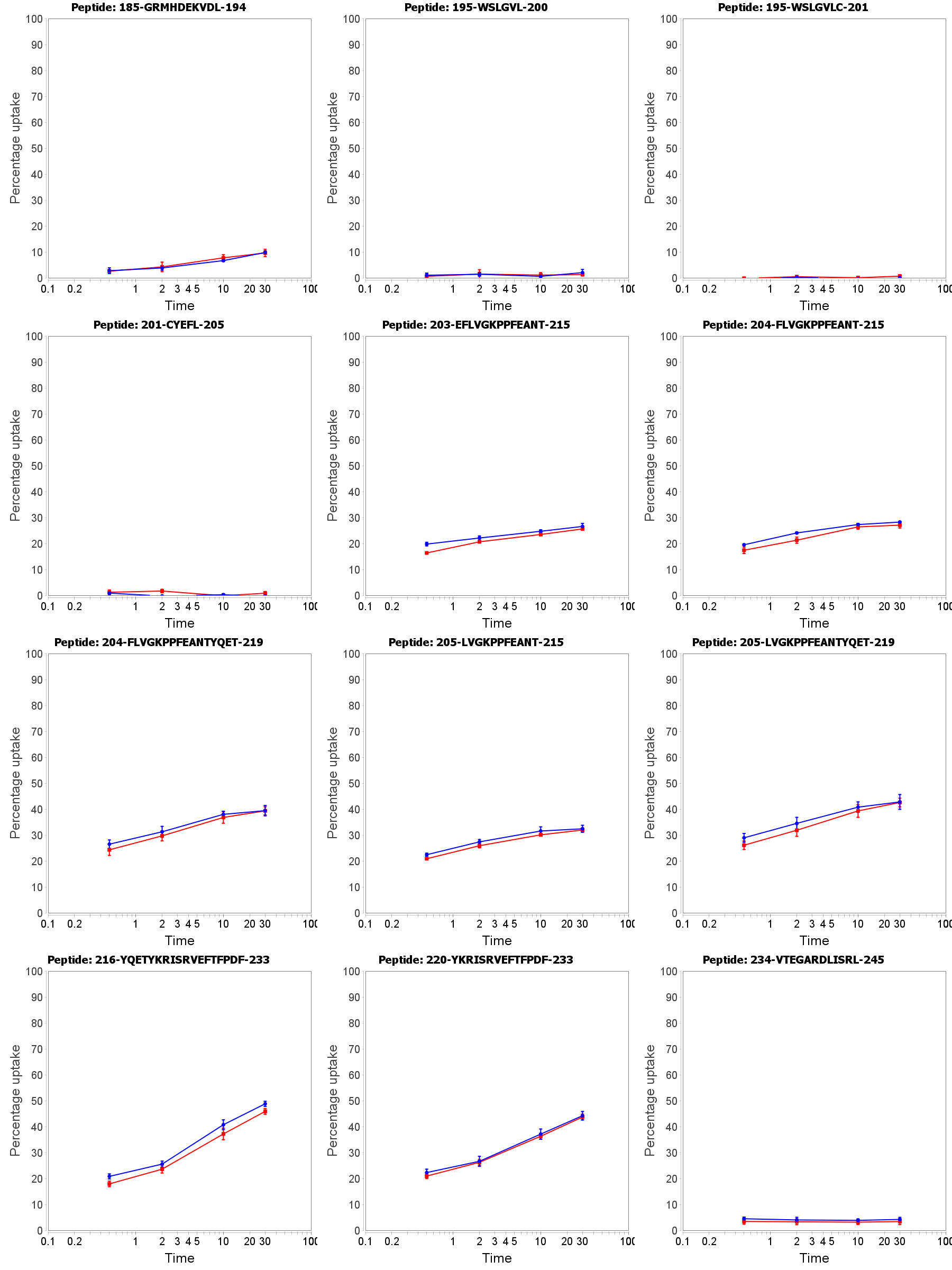

Supplement: Supplementary file 10 — EV and Appendix Figure Source Data [file 44318_2024_240_MOESM10_ESM.zip › Expanded View/EV2/EV2D/Source_Data_Fig_EV2D_HDX_MS_Uptake_Plots_AurA/uptake_6.png]

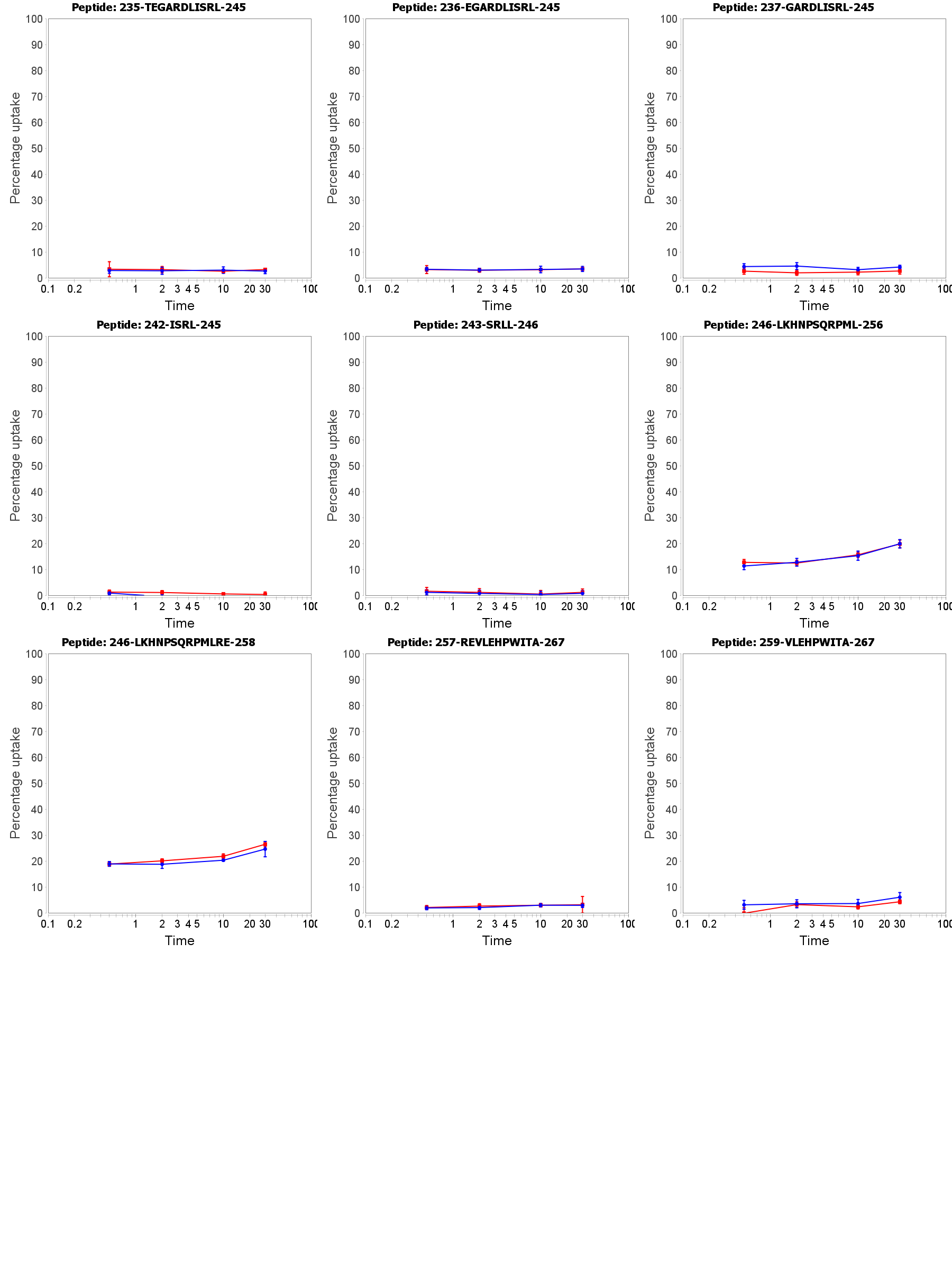

Supplement: Supplementary file 10 — EV and Appendix Figure Source Data [file 44318_2024_240_MOESM10_ESM.zip › Expanded View/EV2/EV2D/Source_Data_Fig_EV2D_HDX_MS_Uptake_Plots_AurA/uptake_7.png]
