# Supplementary figures and images for: CEP192 localises mitotic Aurora-A activity by priming its interaction with TPX2 (part 1 of 2)
Source: EMBO J. 2024 Sep 26;43(22):5381–420. doi: 10.1038/s44318-024-00240-z (PMC11574021; doi:10.1038/s44318-024-00240-z)

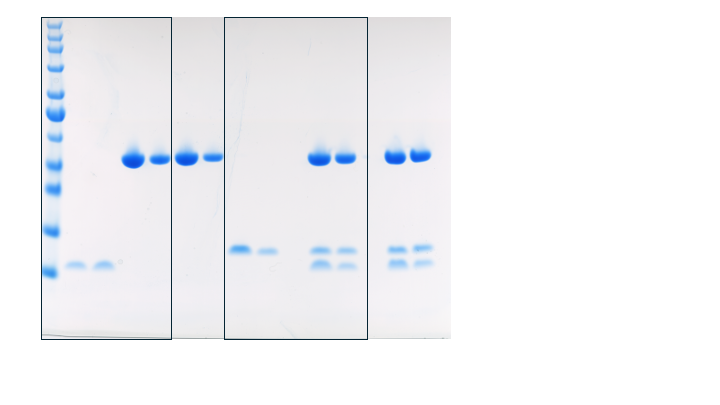

Supplement: Supplementary file 10 — EV and Appendix Figure Source Data [file 44318_2024_240_MOESM10_ESM.zip › Appendix/S3/S3B/Source_Data_Fig_S3B_UncroppedSDSPAGE.tiff]

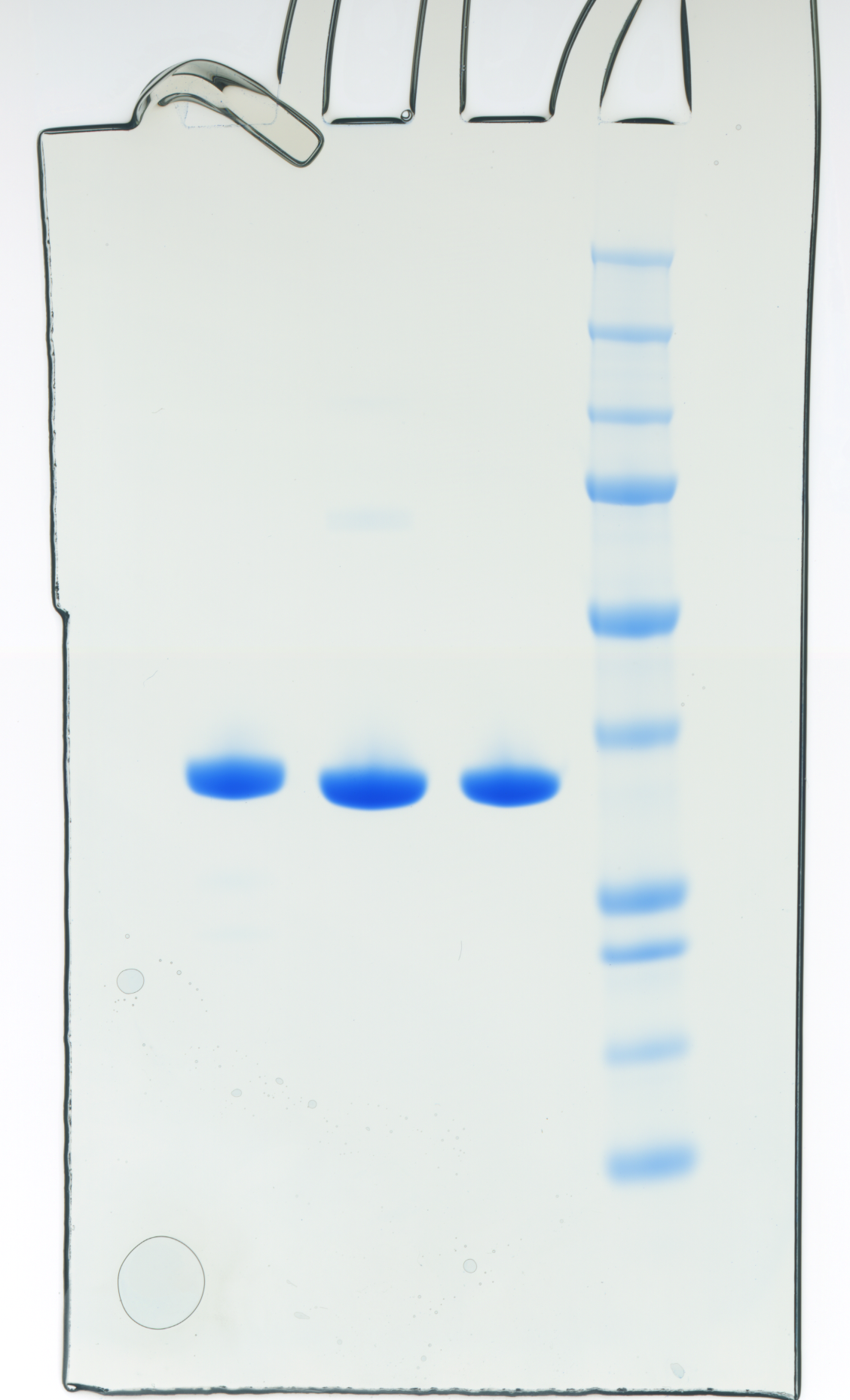

Supplement: Supplementary file 10 — EV and Appendix Figure Source Data [file 44318_2024_240_MOESM10_ESM.zip › Appendix/S4/S4G/Source_Data_Fig_S4G_SDS_PAGE_Aurora_A.tiff]

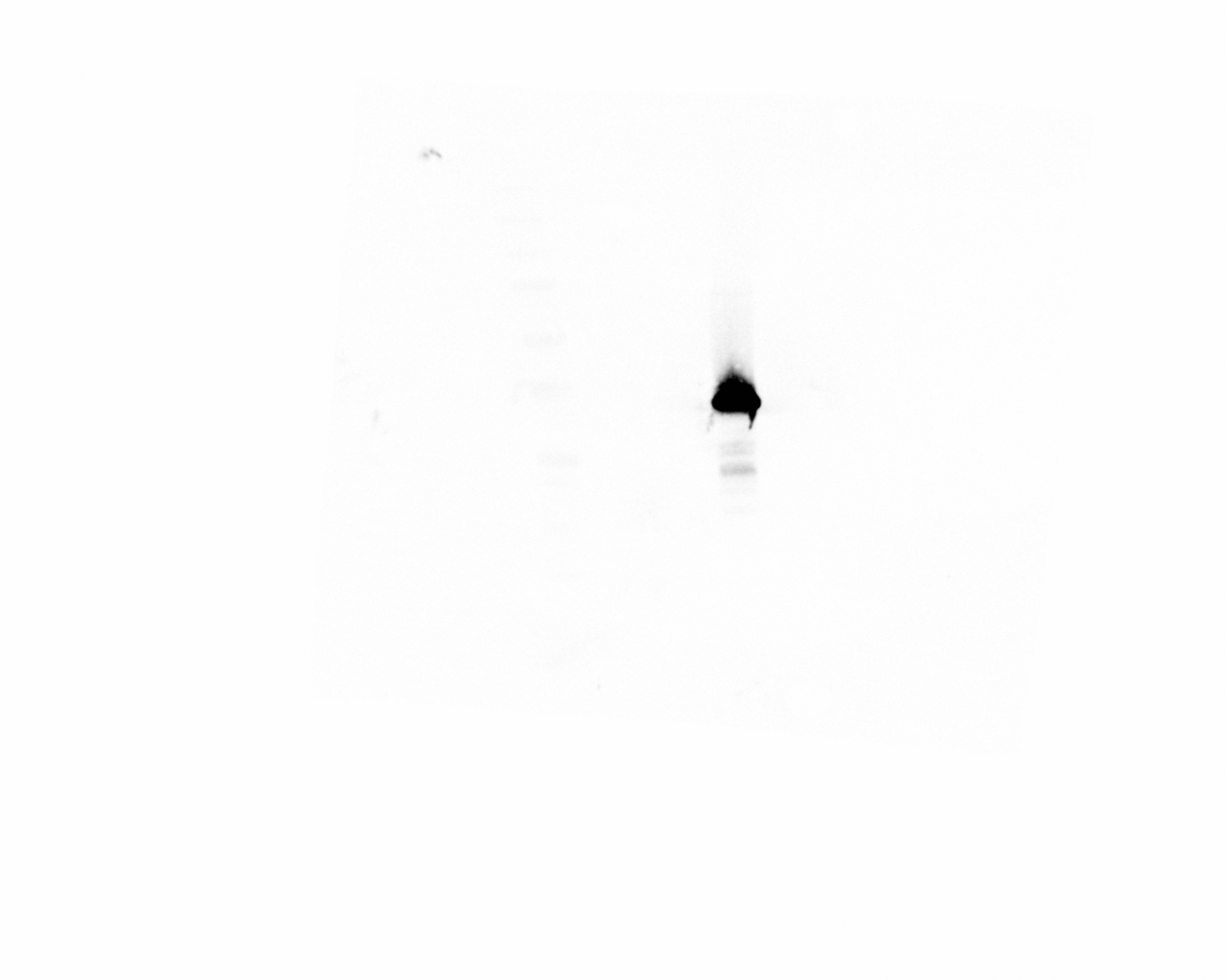

Supplement: Supplementary file 10 — EV and Appendix Figure Source Data [file 44318_2024_240_MOESM10_ESM.zip › Appendix/S4/S4G/Source_Data_Fig_S4G_Western_Anti_Aurora_pT288.tif]

Source Data Appendix Figure S6B  
S6B

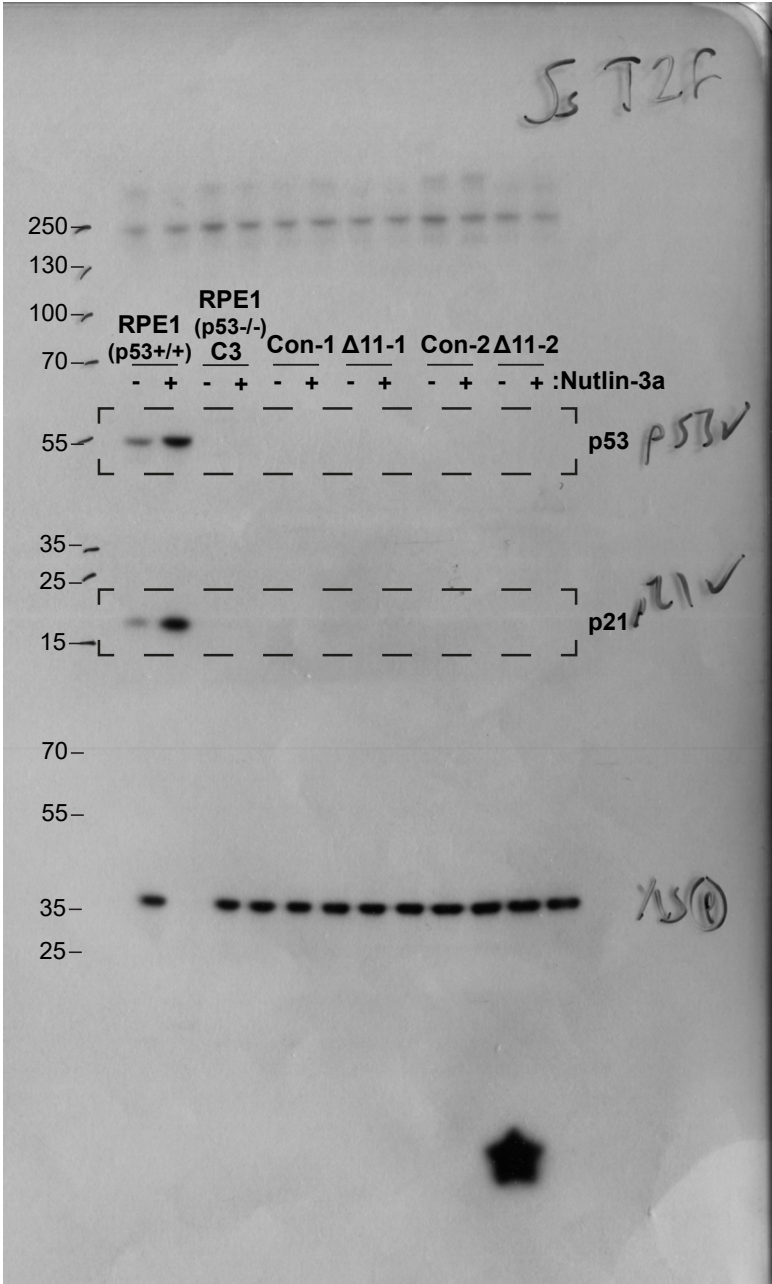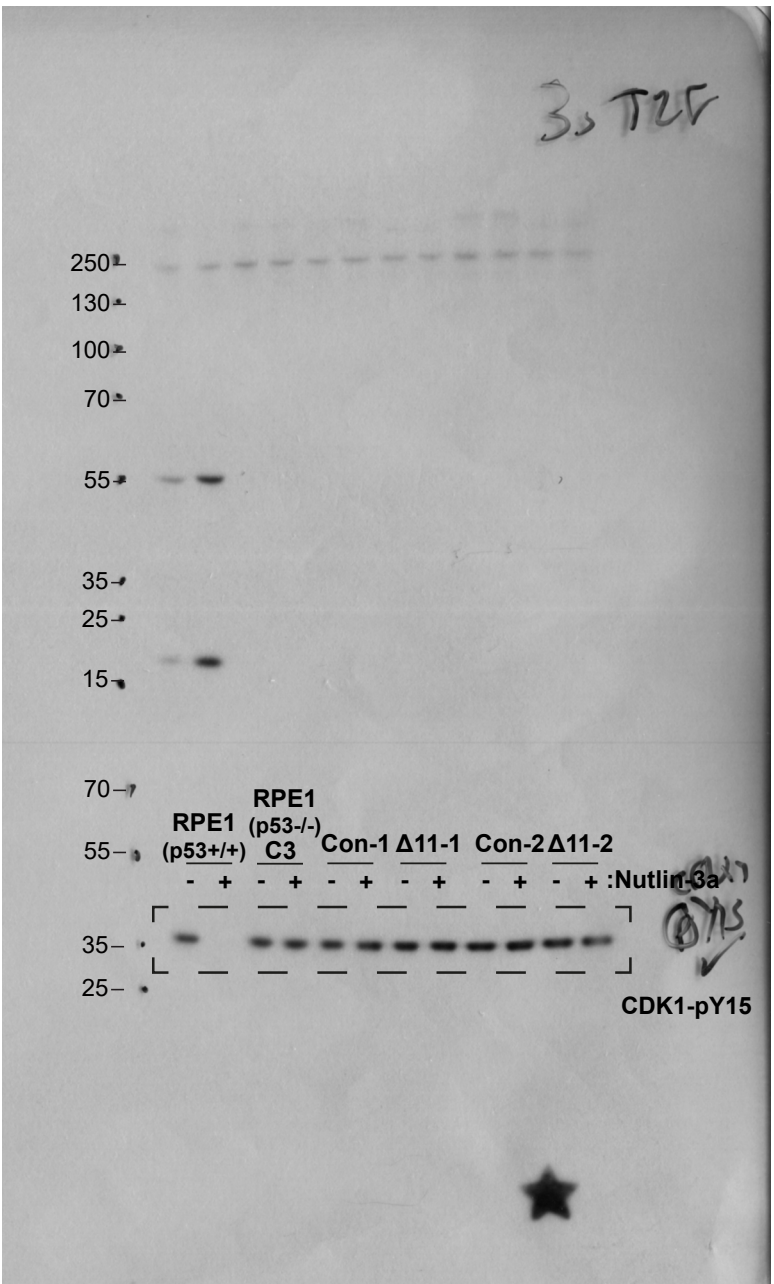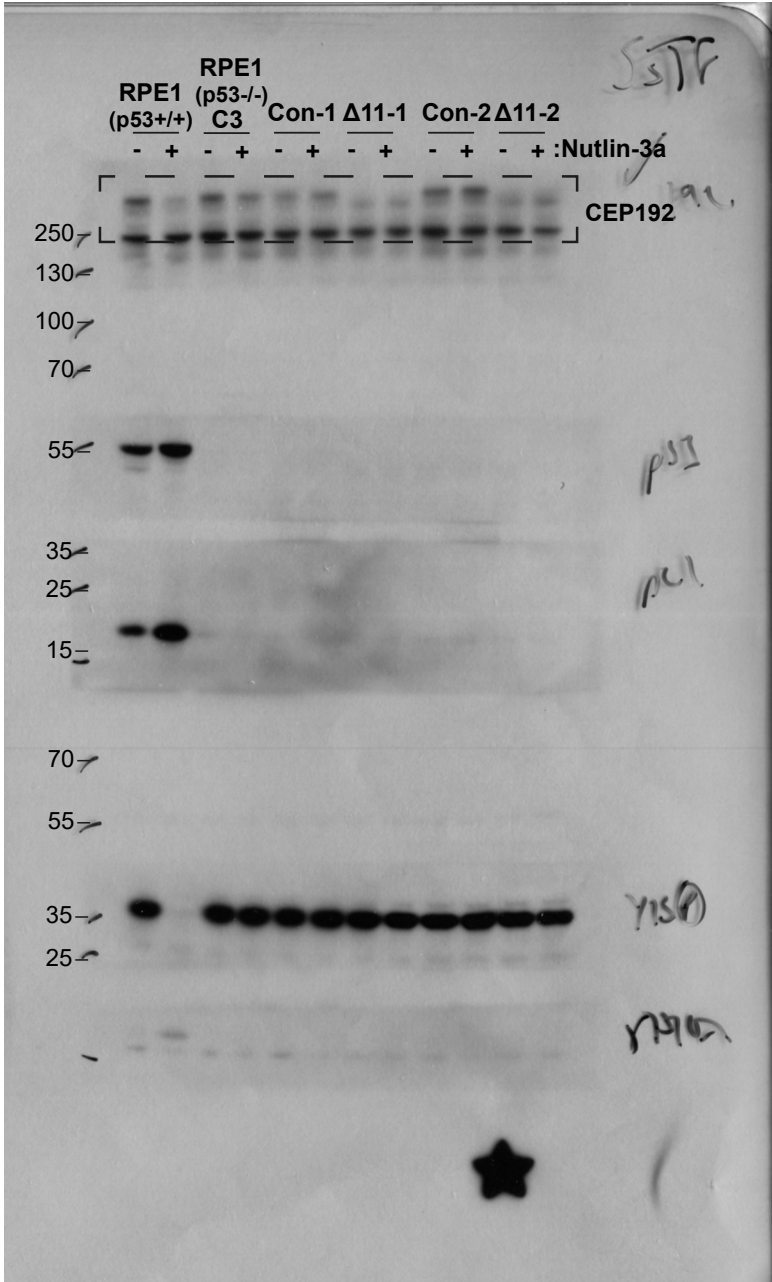

## EV4B

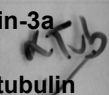

Supplement: Supplementary file 10 — EV and Appendix Figure Source Data [file 44318_2024_240_MOESM10_ESM.zip › Appendix/S6/S6B/Sourcedata_Appendix Figure S6B_Western blots.pdf]

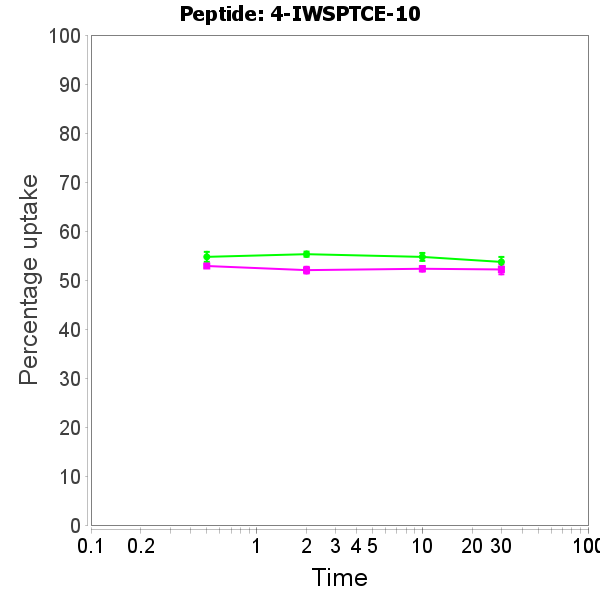

Supplement: Supplementary file 10 — EV and Appendix Figure Source Data [file 44318_2024_240_MOESM10_ESM.zip › Expanded View/EV2/EV2C/Source_Data_Fig_EV2_HDX_MS_CEP192_uptake_plots/CEP/chart_output004-010.png]

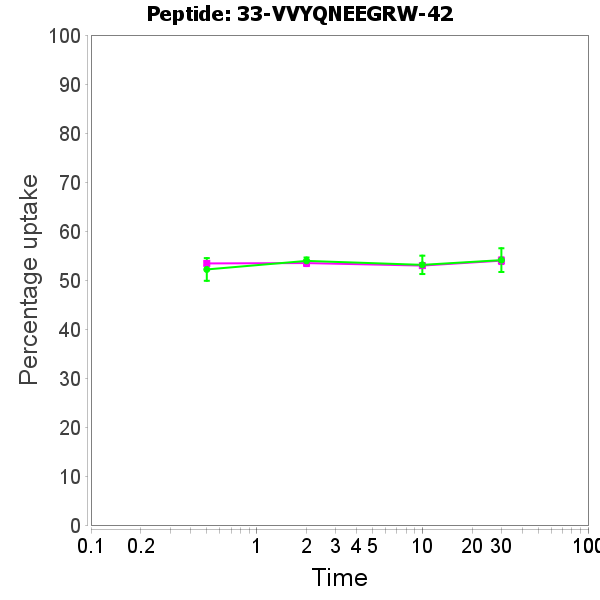

Supplement: Supplementary file 10 — EV and Appendix Figure Source Data [file 44318_2024_240_MOESM10_ESM.zip › Expanded View/EV2/EV2C/Source_Data_Fig_EV2_HDX_MS_CEP192_uptake_plots/CEP/chart_output033-042.png]

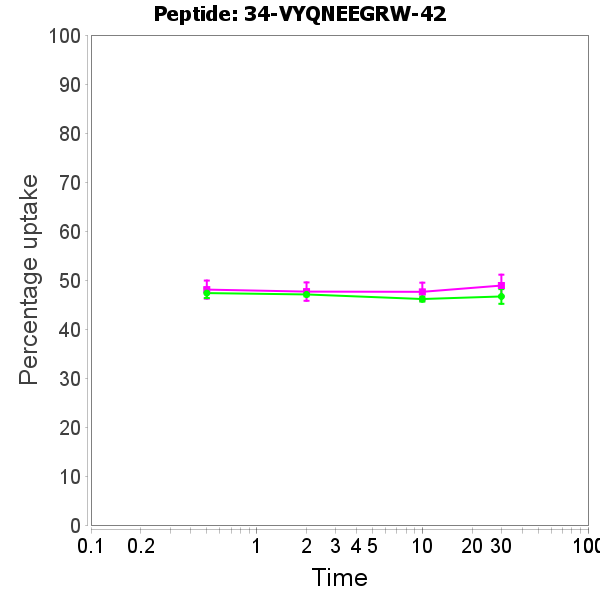

Supplement: Supplementary file 10 — EV and Appendix Figure Source Data [file 44318_2024_240_MOESM10_ESM.zip › Expanded View/EV2/EV2C/Source_Data_Fig_EV2_HDX_MS_CEP192_uptake_plots/CEP/chart_output034-042.png]

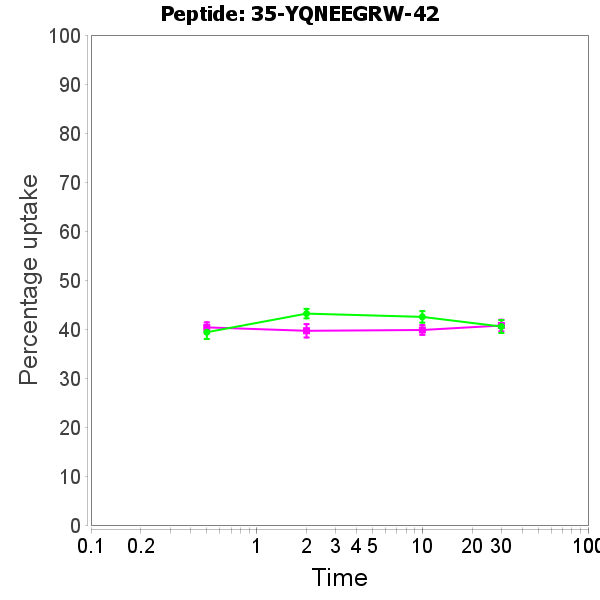

Supplement: Supplementary file 10 — EV and Appendix Figure Source Data [file 44318_2024_240_MOESM10_ESM.zip › Expanded View/EV2/EV2C/Source_Data_Fig_EV2_HDX_MS_CEP192_uptake_plots/CEP/chart_output035-042.png]

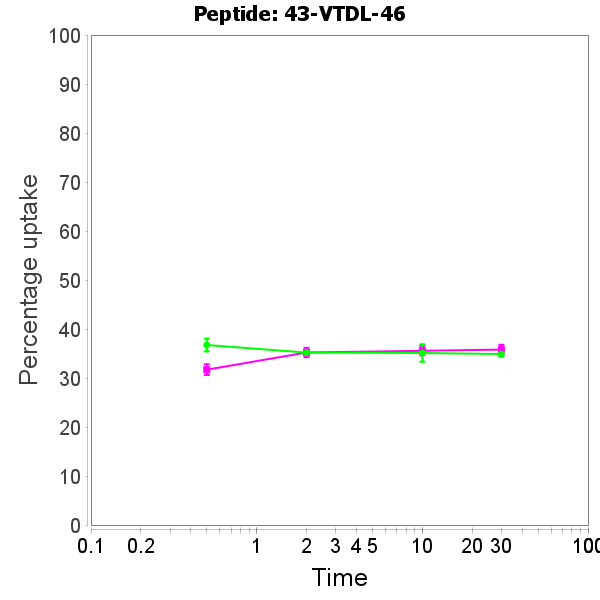

Supplement: Supplementary file 10 — EV and Appendix Figure Source Data [file 44318_2024_240_MOESM10_ESM.zip › Expanded View/EV2/EV2C/Source_Data_Fig_EV2_HDX_MS_CEP192_uptake_plots/CEP/chart_output043-046.png]

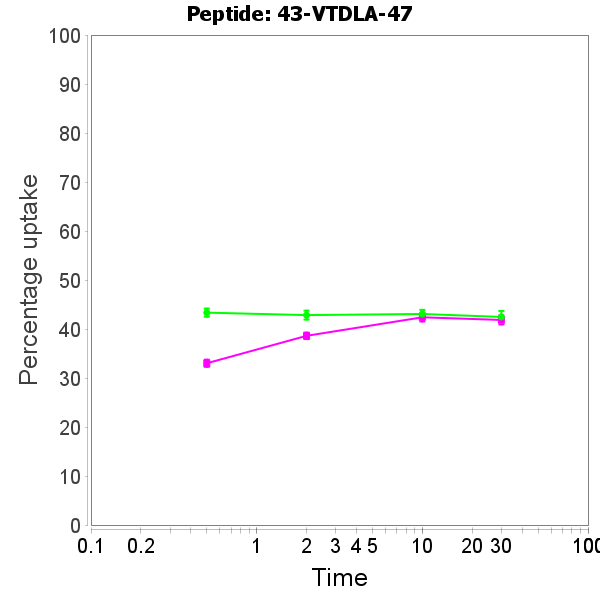

Supplement: Supplementary file 10 — EV and Appendix Figure Source Data [file 44318_2024_240_MOESM10_ESM.zip › Expanded View/EV2/EV2C/Source_Data_Fig_EV2_HDX_MS_CEP192_uptake_plots/CEP/chart_output043-047.png]

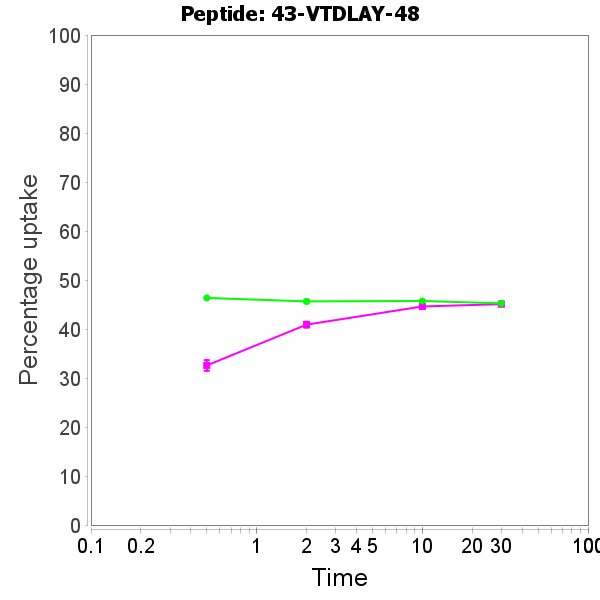

Supplement: Supplementary file 10 — EV and Appendix Figure Source Data [file 44318_2024_240_MOESM10_ESM.zip › Expanded View/EV2/EV2C/Source_Data_Fig_EV2_HDX_MS_CEP192_uptake_plots/CEP/chart_output043-048.png]

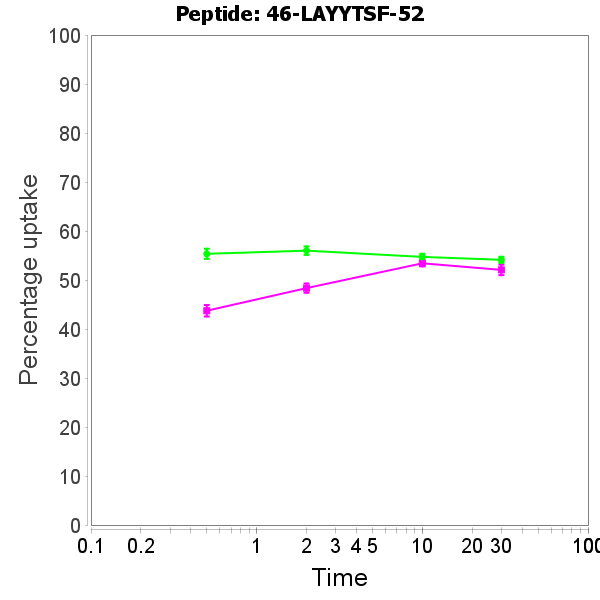

Supplement: Supplementary file 10 — EV and Appendix Figure Source Data [file 44318_2024_240_MOESM10_ESM.zip › Expanded View/EV2/EV2C/Source_Data_Fig_EV2_HDX_MS_CEP192_uptake_plots/CEP/chart_output046-052.png]

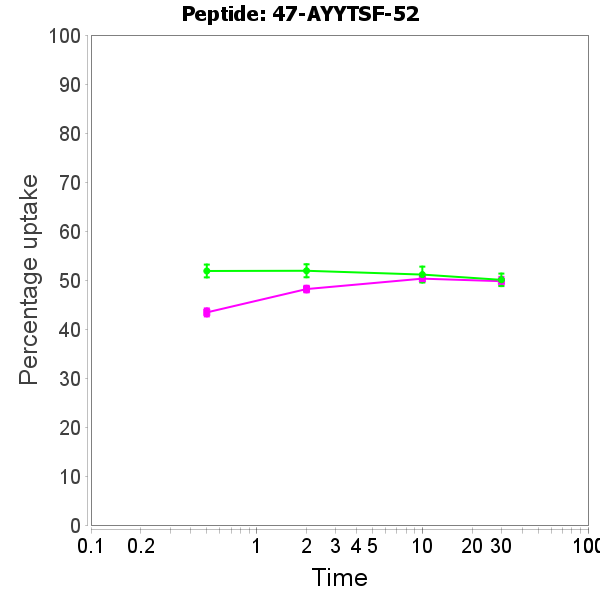

Supplement: Supplementary file 10 — EV and Appendix Figure Source Data [file 44318_2024_240_MOESM10_ESM.zip › Expanded View/EV2/EV2C/Source_Data_Fig_EV2_HDX_MS_CEP192_uptake_plots/CEP/chart_output047-052.png]

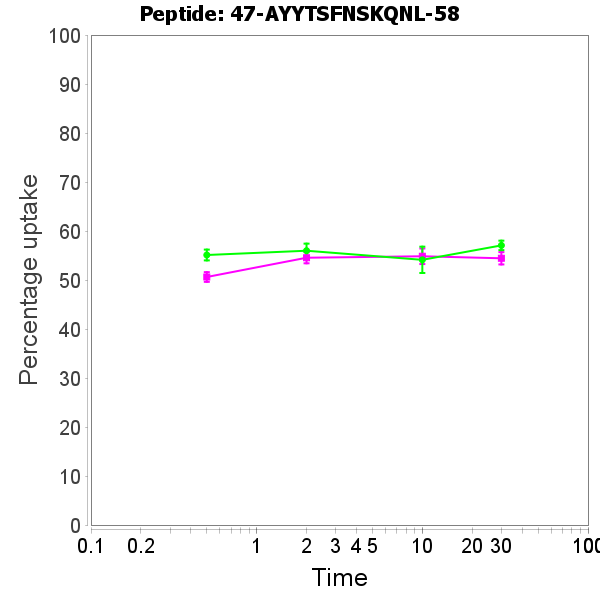

Supplement: Supplementary file 10 — EV and Appendix Figure Source Data [file 44318_2024_240_MOESM10_ESM.zip › Expanded View/EV2/EV2C/Source_Data_Fig_EV2_HDX_MS_CEP192_uptake_plots/CEP/chart_output047-058.png]

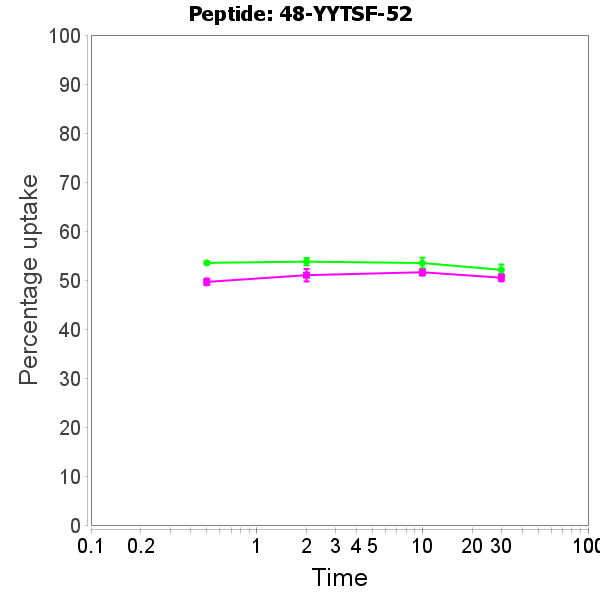

Supplement: Supplementary file 10 — EV and Appendix Figure Source Data [file 44318_2024_240_MOESM10_ESM.zip › Expanded View/EV2/EV2C/Source_Data_Fig_EV2_HDX_MS_CEP192_uptake_plots/CEP/chart_output048-052.png]

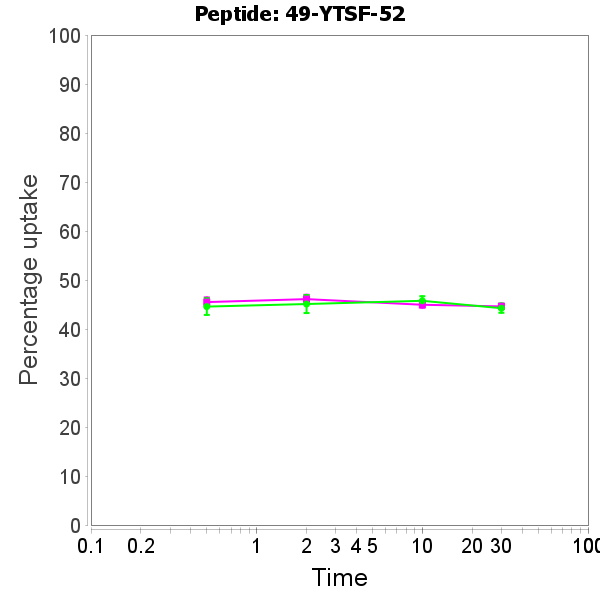

Supplement: Supplementary file 10 — EV and Appendix Figure Source Data [file 44318_2024_240_MOESM10_ESM.zip › Expanded View/EV2/EV2C/Source_Data_Fig_EV2_HDX_MS_CEP192_uptake_plots/CEP/chart_output049-052.png]

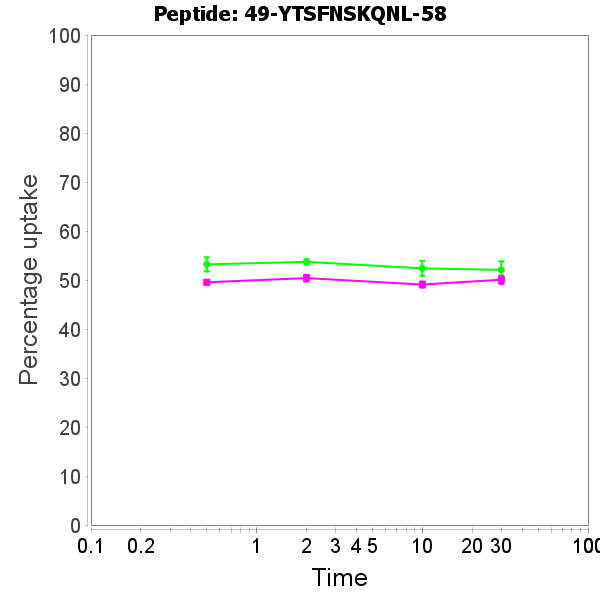

Supplement: Supplementary file 10 — EV and Appendix Figure Source Data [file 44318_2024_240_MOESM10_ESM.zip › Expanded View/EV2/EV2C/Source_Data_Fig_EV2_HDX_MS_CEP192_uptake_plots/CEP/chart_output049-058.png]

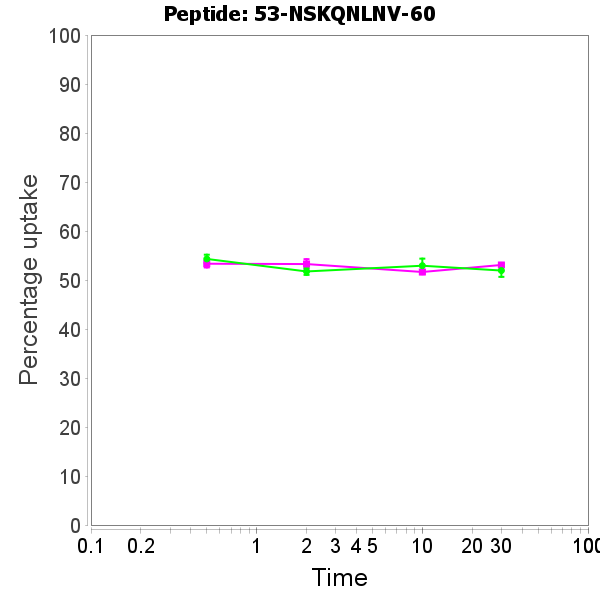

Supplement: Supplementary file 10 — EV and Appendix Figure Source Data [file 44318_2024_240_MOESM10_ESM.zip › Expanded View/EV2/EV2C/Source_Data_Fig_EV2_HDX_MS_CEP192_uptake_plots/CEP/chart_output053-060.png]

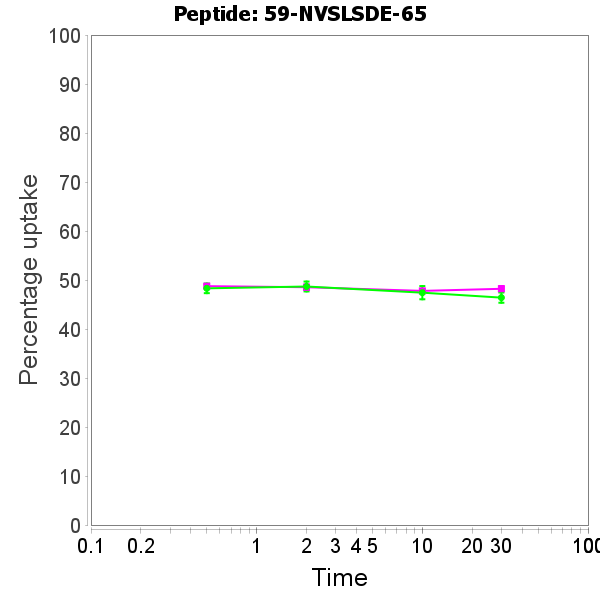

Supplement: Supplementary file 10 — EV and Appendix Figure Source Data [file 44318_2024_240_MOESM10_ESM.zip › Expanded View/EV2/EV2C/Source_Data_Fig_EV2_HDX_MS_CEP192_uptake_plots/CEP/chart_output059-065.png]

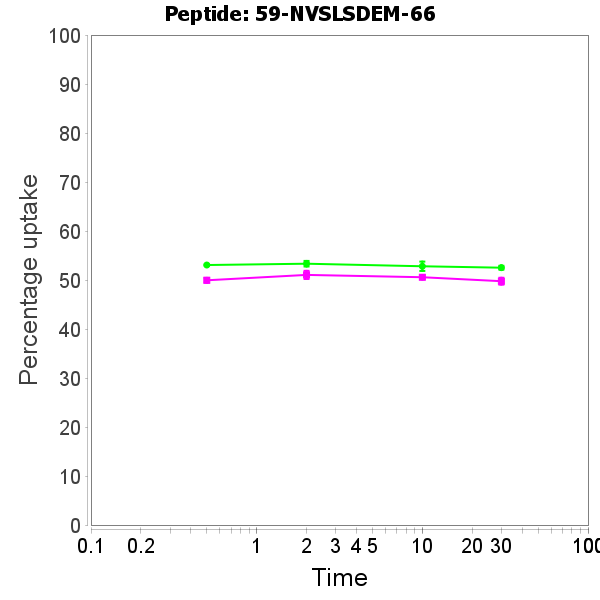

Supplement: Supplementary file 10 — EV and Appendix Figure Source Data [file 44318_2024_240_MOESM10_ESM.zip › Expanded View/EV2/EV2C/Source_Data_Fig_EV2_HDX_MS_CEP192_uptake_plots/CEP/chart_output059-066.png]

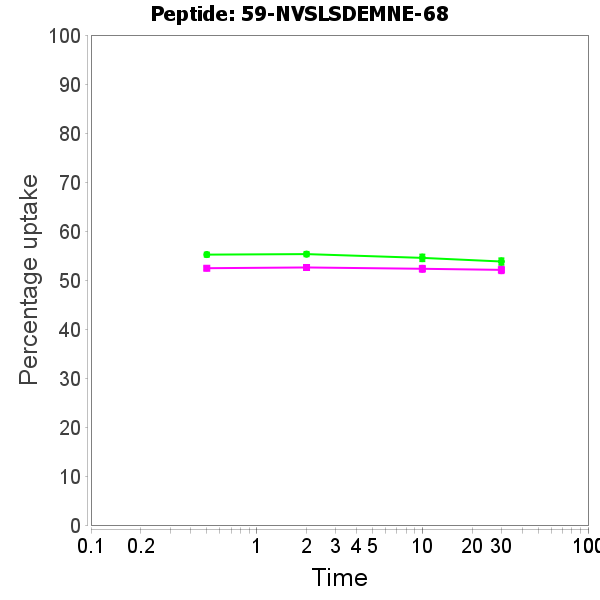

Supplement: Supplementary file 10 — EV and Appendix Figure Source Data [file 44318_2024_240_MOESM10_ESM.zip › Expanded View/EV2/EV2C/Source_Data_Fig_EV2_HDX_MS_CEP192_uptake_plots/CEP/chart_output059-068.png]

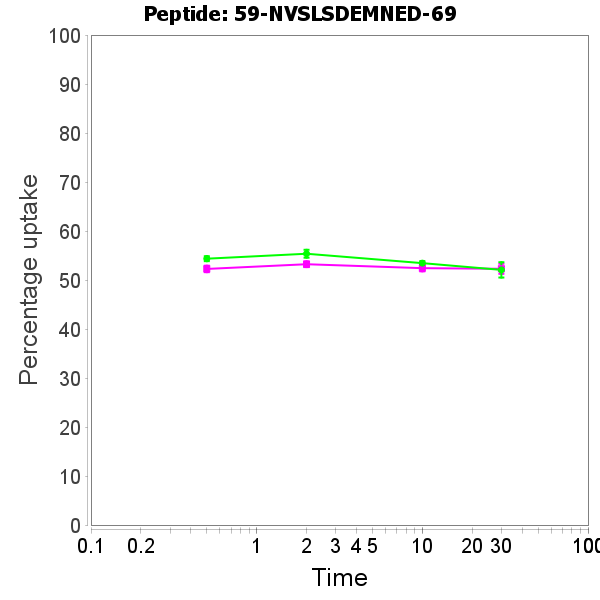

Supplement: Supplementary file 10 — EV and Appendix Figure Source Data [file 44318_2024_240_MOESM10_ESM.zip › Expanded View/EV2/EV2C/Source_Data_Fig_EV2_HDX_MS_CEP192_uptake_plots/CEP/chart_output059-069.png]

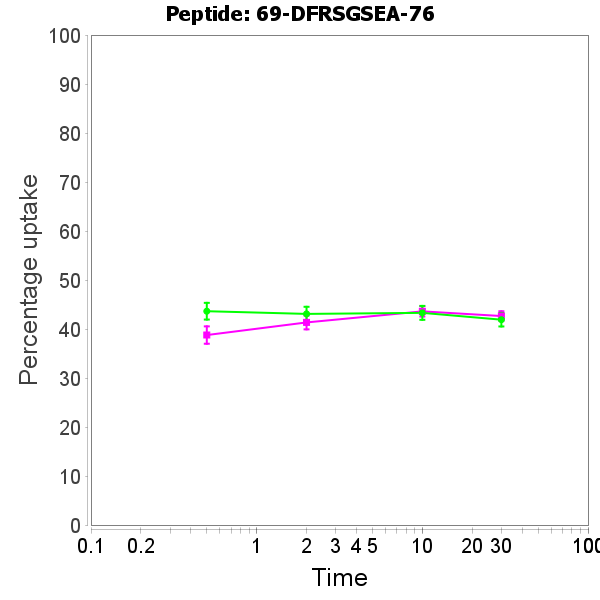

Supplement: Supplementary file 10 — EV and Appendix Figure Source Data [file 44318_2024_240_MOESM10_ESM.zip › Expanded View/EV2/EV2C/Source_Data_Fig_EV2_HDX_MS_CEP192_uptake_plots/CEP/chart_output069-076.png]

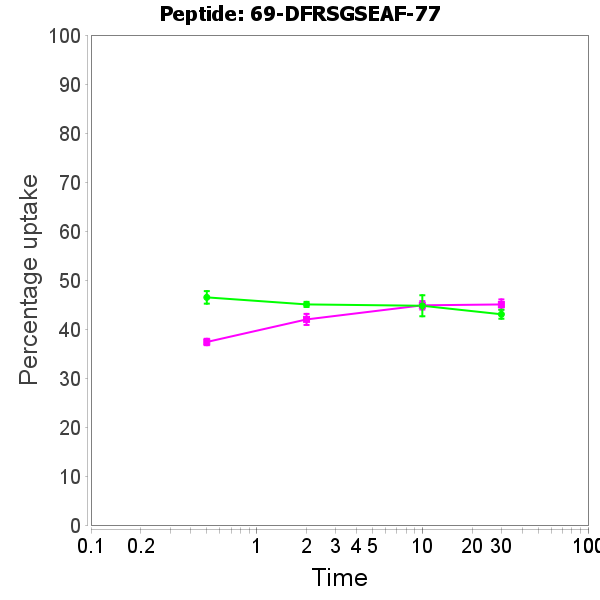

Supplement: Supplementary file 10 — EV and Appendix Figure Source Data [file 44318_2024_240_MOESM10_ESM.zip › Expanded View/EV2/EV2C/Source_Data_Fig_EV2_HDX_MS_CEP192_uptake_plots/CEP/chart_output069-077.png]

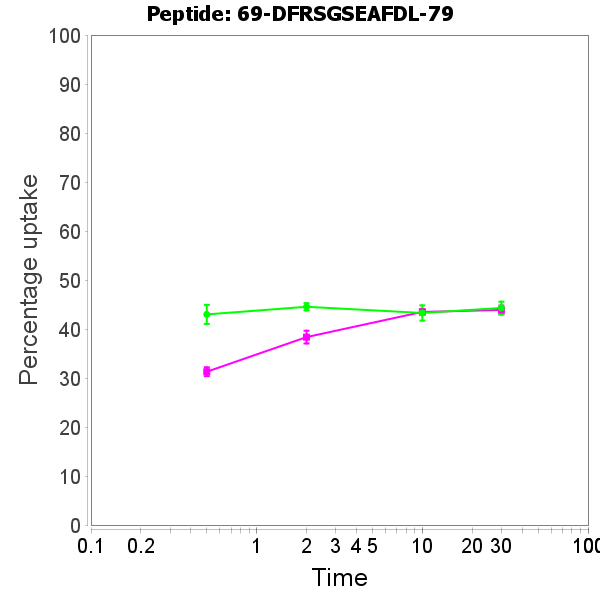

Supplement: Supplementary file 10 — EV and Appendix Figure Source Data [file 44318_2024_240_MOESM10_ESM.zip › Expanded View/EV2/EV2C/Source_Data_Fig_EV2_HDX_MS_CEP192_uptake_plots/CEP/chart_output069-079.png]

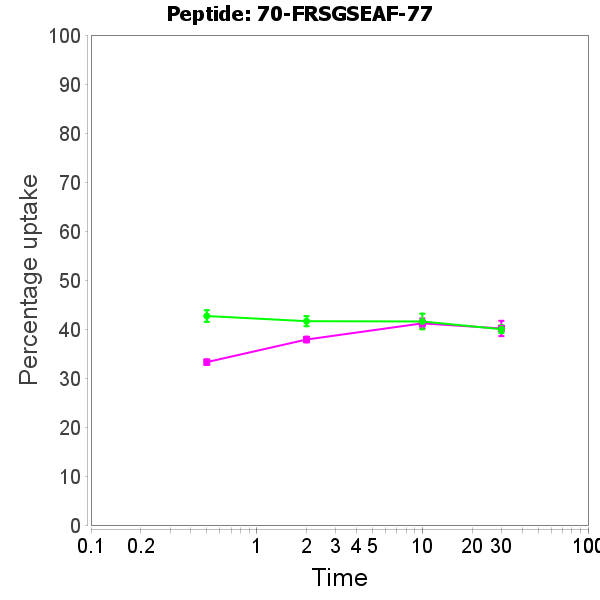

Supplement: Supplementary file 10 — EV and Appendix Figure Source Data [file 44318_2024_240_MOESM10_ESM.zip › Expanded View/EV2/EV2C/Source_Data_Fig_EV2_HDX_MS_CEP192_uptake_plots/CEP/chart_output070-077.png]

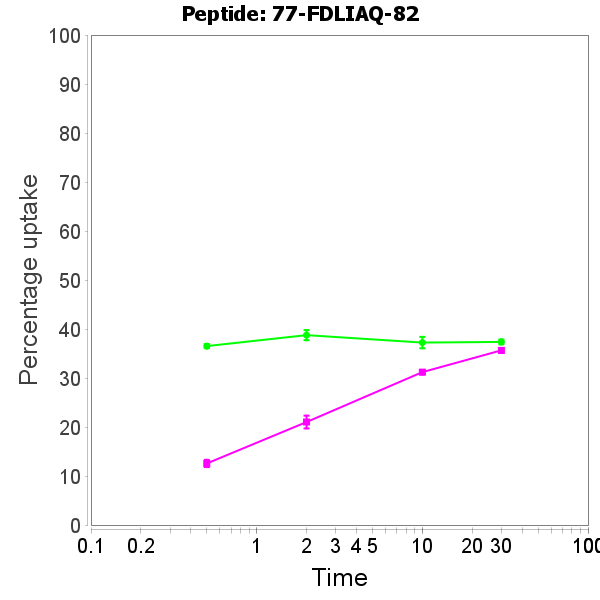

Supplement: Supplementary file 10 — EV and Appendix Figure Source Data [file 44318_2024_240_MOESM10_ESM.zip › Expanded View/EV2/EV2C/Source_Data_Fig_EV2_HDX_MS_CEP192_uptake_plots/CEP/chart_output077-082.png]

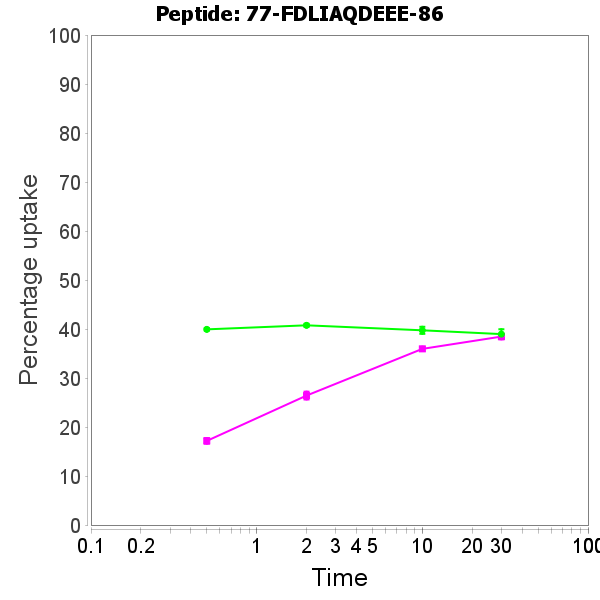

Supplement: Supplementary file 10 — EV and Appendix Figure Source Data [file 44318_2024_240_MOESM10_ESM.zip › Expanded View/EV2/EV2C/Source_Data_Fig_EV2_HDX_MS_CEP192_uptake_plots/CEP/chart_output077-086.png]

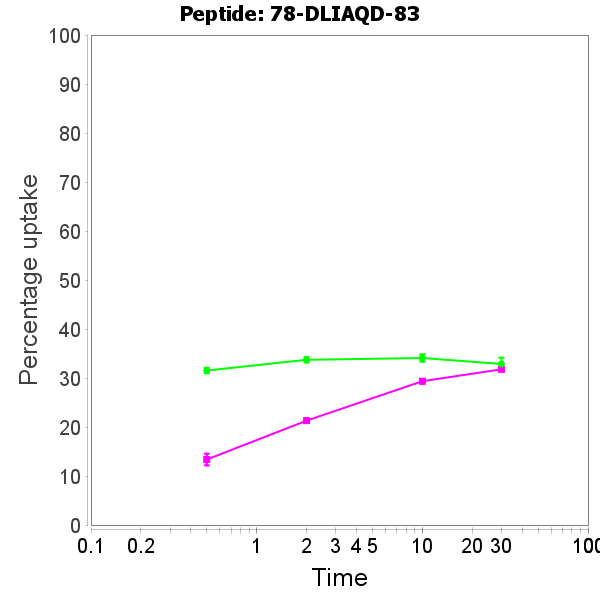

Supplement: Supplementary file 10 — EV and Appendix Figure Source Data [file 44318_2024_240_MOESM10_ESM.zip › Expanded View/EV2/EV2C/Source_Data_Fig_EV2_HDX_MS_CEP192_uptake_plots/CEP/chart_output078-083.png]

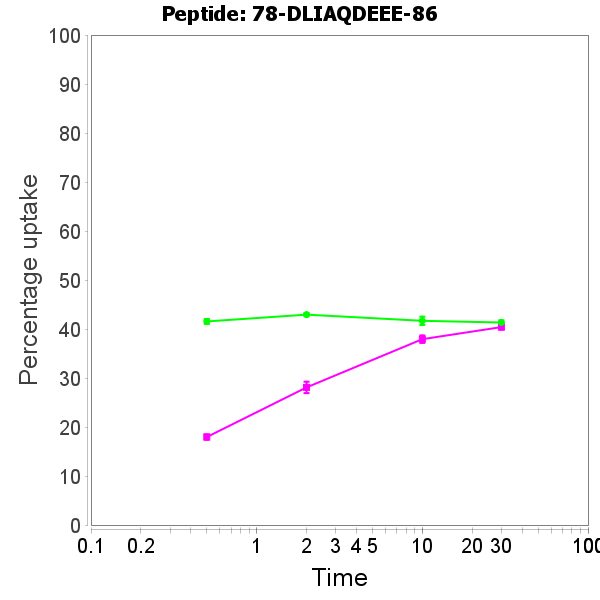

Supplement: Supplementary file 10 — EV and Appendix Figure Source Data [file 44318_2024_240_MOESM10_ESM.zip › Expanded View/EV2/EV2C/Source_Data_Fig_EV2_HDX_MS_CEP192_uptake_plots/CEP/chart_output078-086.png]

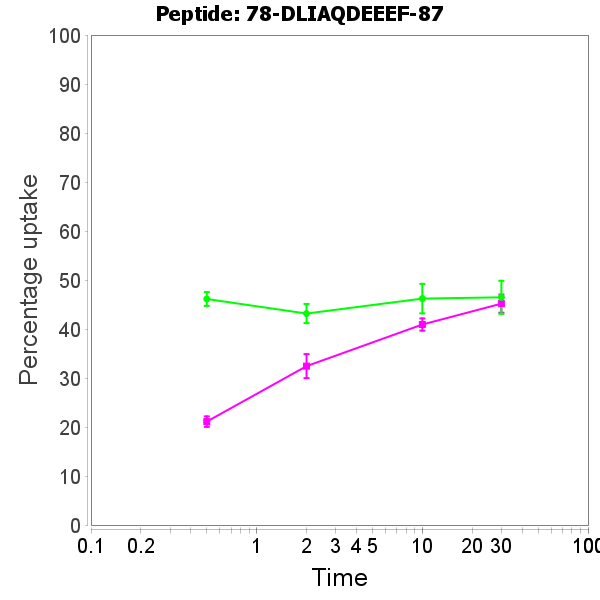

Supplement: Supplementary file 10 — EV and Appendix Figure Source Data [file 44318_2024_240_MOESM10_ESM.zip › Expanded View/EV2/EV2C/Source_Data_Fig_EV2_HDX_MS_CEP192_uptake_plots/CEP/chart_output078-087.png]

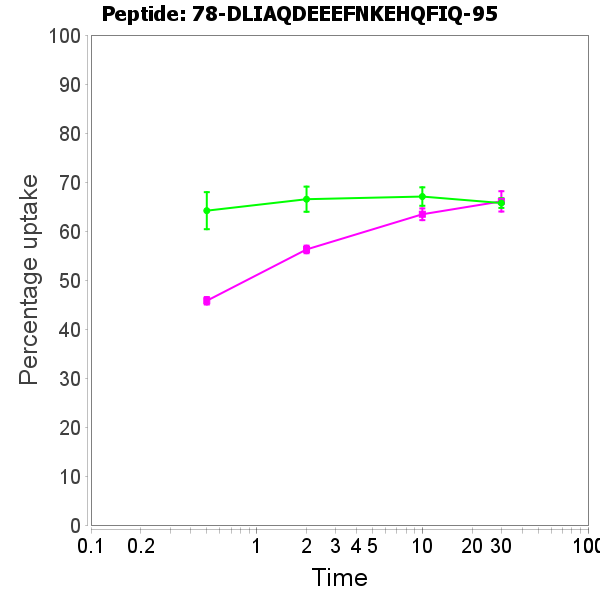

Supplement: Supplementary file 10 — EV and Appendix Figure Source Data [file 44318_2024_240_MOESM10_ESM.zip › Expanded View/EV2/EV2C/Source_Data_Fig_EV2_HDX_MS_CEP192_uptake_plots/CEP/chart_output078-095.png]

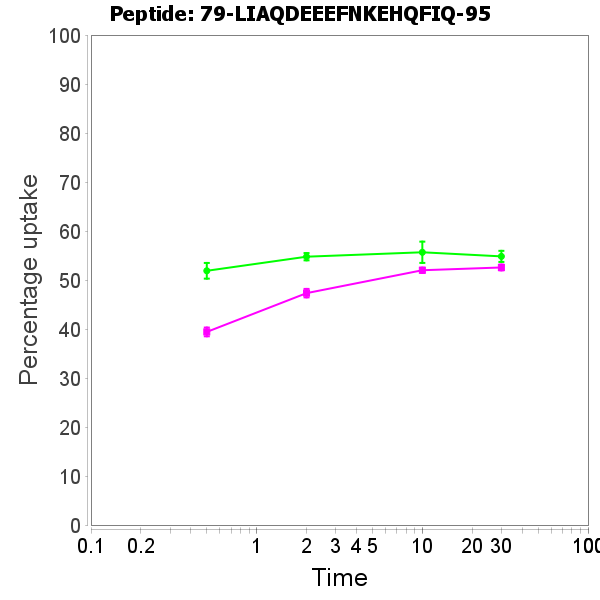

Supplement: Supplementary file 10 — EV and Appendix Figure Source Data [file 44318_2024_240_MOESM10_ESM.zip › Expanded View/EV2/EV2C/Source_Data_Fig_EV2_HDX_MS_CEP192_uptake_plots/CEP/chart_output079-095.png]

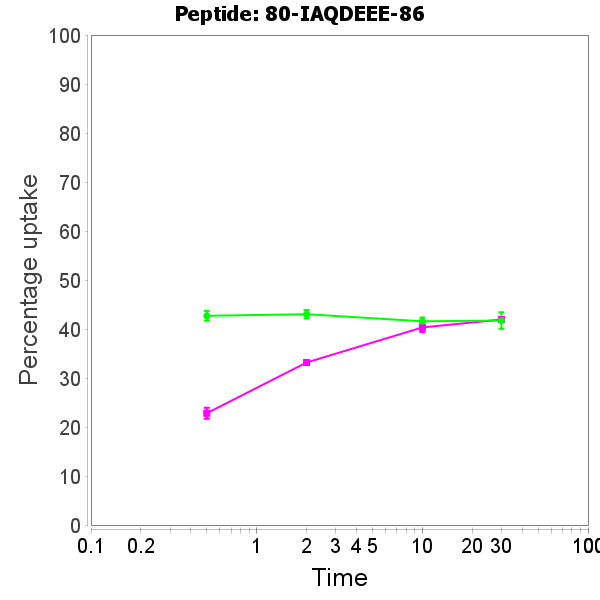

Supplement: Supplementary file 10 — EV and Appendix Figure Source Data [file 44318_2024_240_MOESM10_ESM.zip › Expanded View/EV2/EV2C/Source_Data_Fig_EV2_HDX_MS_CEP192_uptake_plots/CEP/chart_output080-086.png]

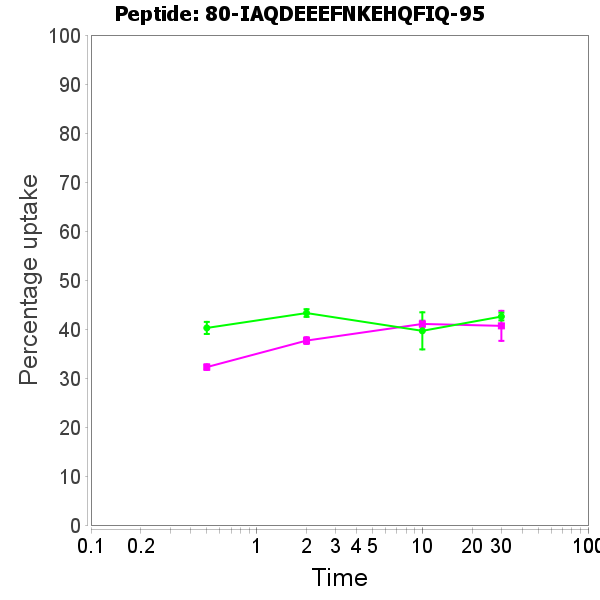

Supplement: Supplementary file 10 — EV and Appendix Figure Source Data [file 44318_2024_240_MOESM10_ESM.zip › Expanded View/EV2/EV2C/Source_Data_Fig_EV2_HDX_MS_CEP192_uptake_plots/CEP/chart_output080-095.png]

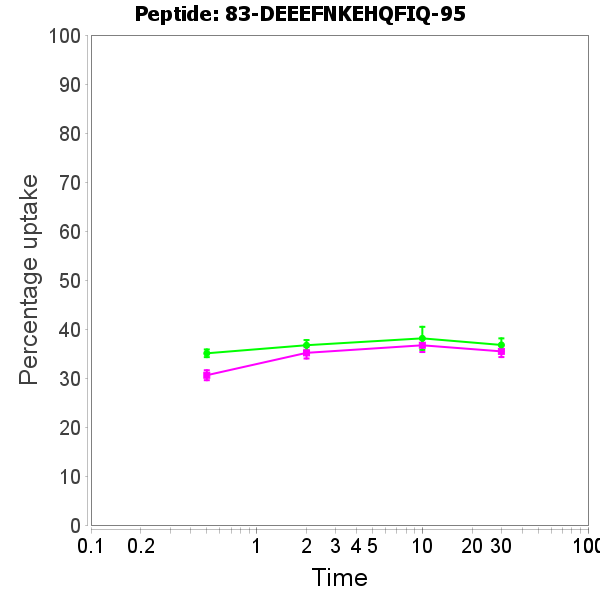

Supplement: Supplementary file 10 — EV and Appendix Figure Source Data [file 44318_2024_240_MOESM10_ESM.zip › Expanded View/EV2/EV2C/Source_Data_Fig_EV2_HDX_MS_CEP192_uptake_plots/CEP/chart_output083-095.png]

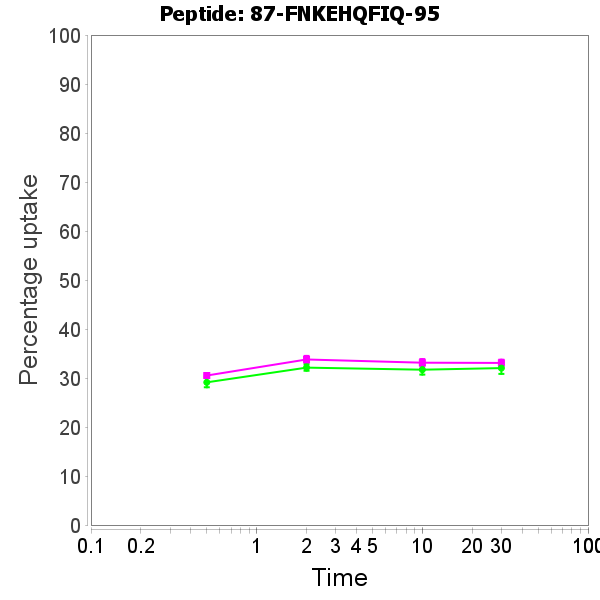

Supplement: Supplementary file 10 — EV and Appendix Figure Source Data [file 44318_2024_240_MOESM10_ESM.zip › Expanded View/EV2/EV2C/Source_Data_Fig_EV2_HDX_MS_CEP192_uptake_plots/CEP/chart_output087-095.png]

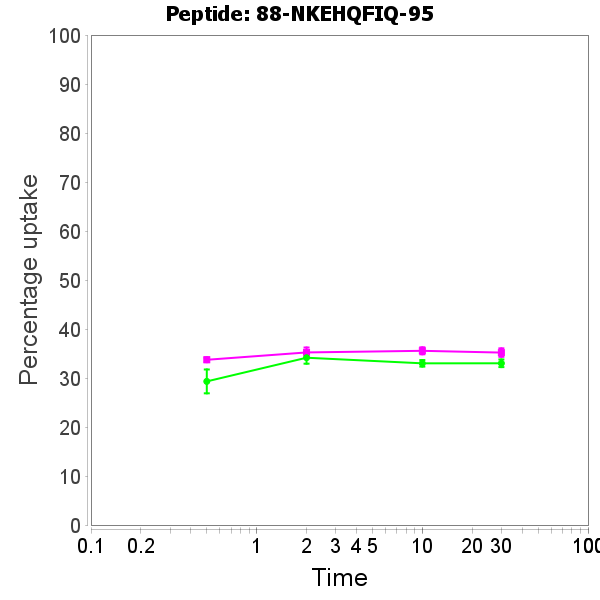

Supplement: Supplementary file 10 — EV and Appendix Figure Source Data [file 44318_2024_240_MOESM10_ESM.zip › Expanded View/EV2/EV2C/Source_Data_Fig_EV2_HDX_MS_CEP192_uptake_plots/CEP/chart_output088-095.png]

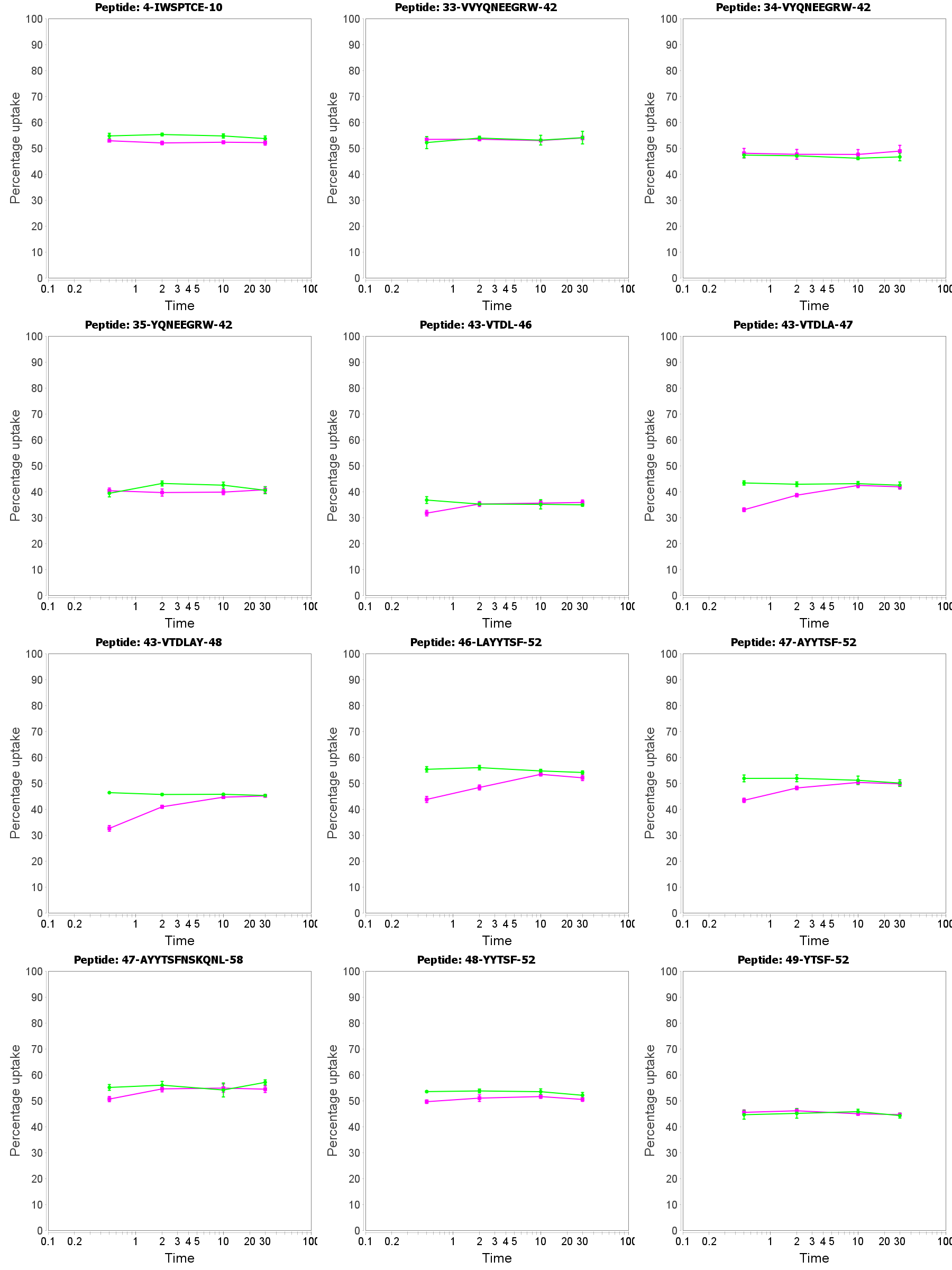

Supplement: Supplementary file 10 — EV and Appendix Figure Source Data [file 44318_2024_240_MOESM10_ESM.zip › Expanded View/EV2/EV2C/Source_Data_Fig_EV2_HDX_MS_CEP192_uptake_plots/CEP/uptake_1.png]

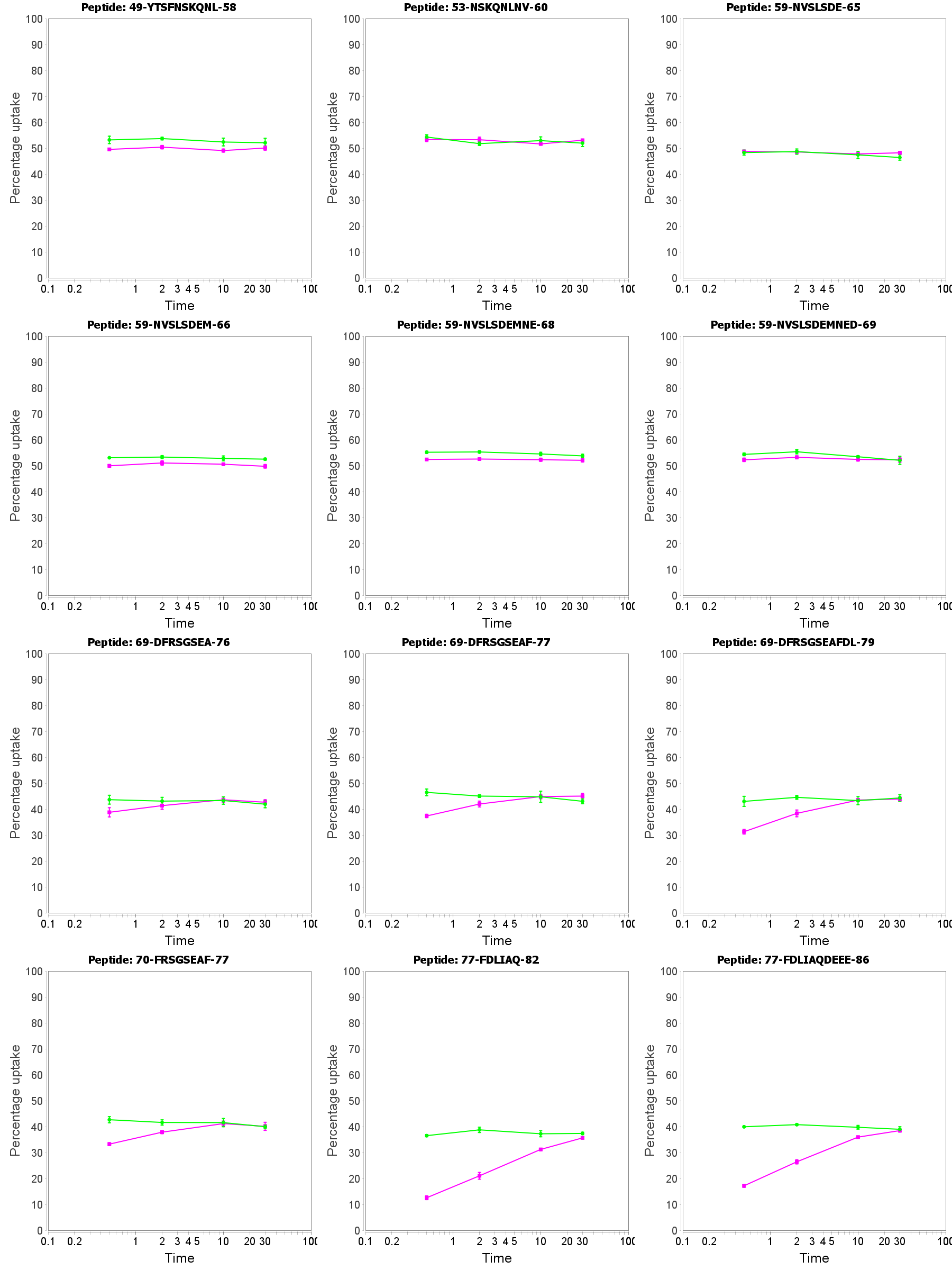

Supplement: Supplementary file 10 — EV and Appendix Figure Source Data [file 44318_2024_240_MOESM10_ESM.zip › Expanded View/EV2/EV2C/Source_Data_Fig_EV2_HDX_MS_CEP192_uptake_plots/CEP/uptake_2.png]

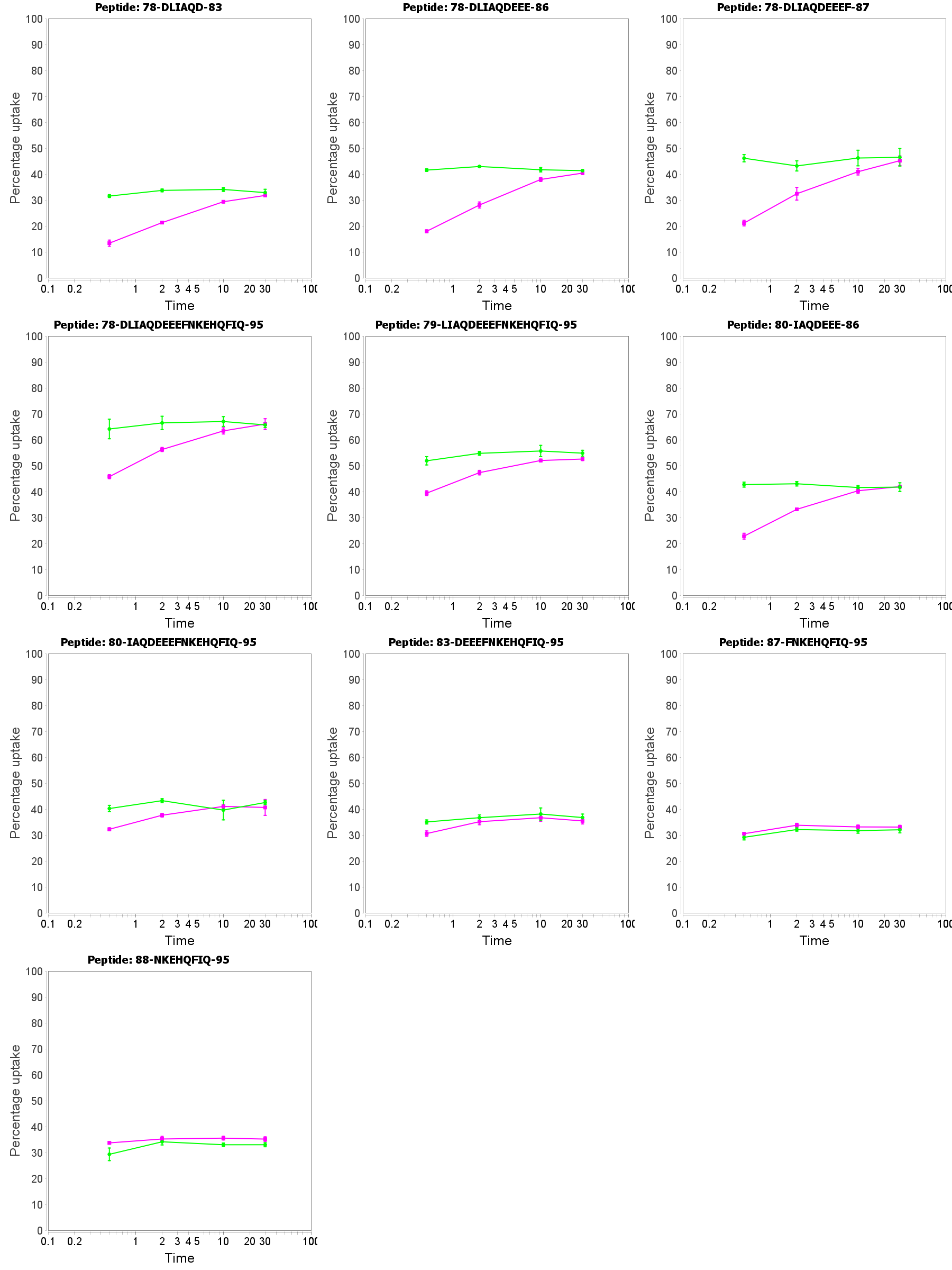

Supplement: Supplementary file 10 — EV and Appendix Figure Source Data [file 44318_2024_240_MOESM10_ESM.zip › Expanded View/EV2/EV2C/Source_Data_Fig_EV2_HDX_MS_CEP192_uptake_plots/CEP/uptake_3.png]

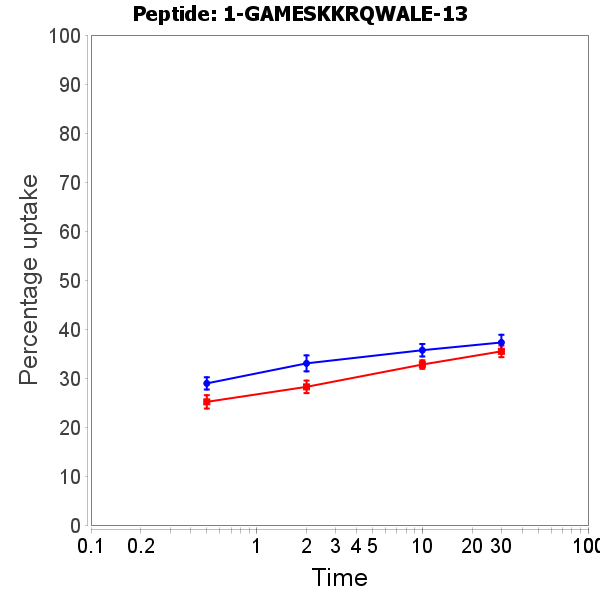

Supplement: Supplementary file 10 — EV and Appendix Figure Source Data [file 44318_2024_240_MOESM10_ESM.zip › Expanded View/EV2/EV2D/Source_Data_Fig_EV2D_HDX_MS_Uptake_Plots_AurA/chart_output001-013.png]

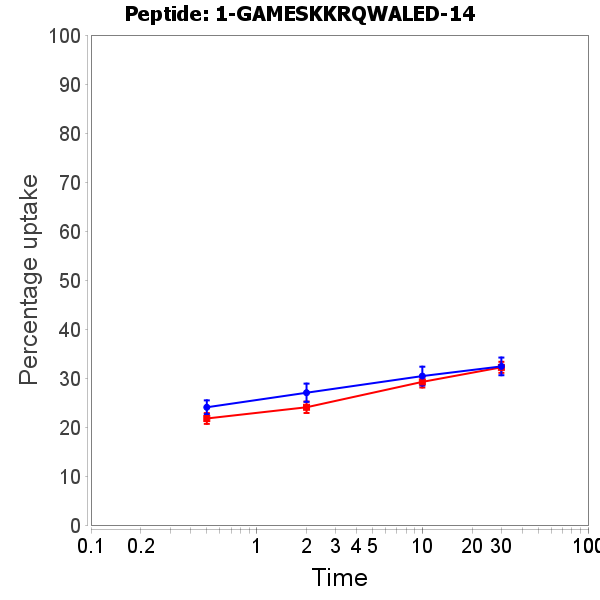

Supplement: Supplementary file 10 — EV and Appendix Figure Source Data [file 44318_2024_240_MOESM10_ESM.zip › Expanded View/EV2/EV2D/Source_Data_Fig_EV2D_HDX_MS_Uptake_Plots_AurA/chart_output001-014.png]

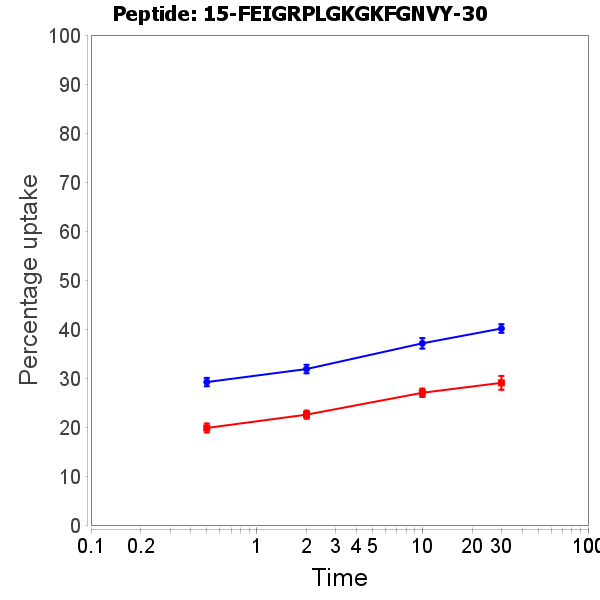

Supplement: Supplementary file 10 — EV and Appendix Figure Source Data [file 44318_2024_240_MOESM10_ESM.zip › Expanded View/EV2/EV2D/Source_Data_Fig_EV2D_HDX_MS_Uptake_Plots_AurA/chart_output015-030.png]

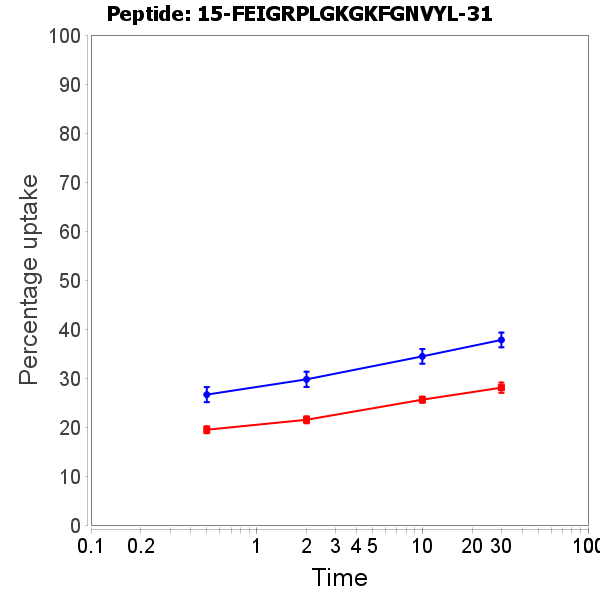

Supplement: Supplementary file 10 — EV and Appendix Figure Source Data [file 44318_2024_240_MOESM10_ESM.zip › Expanded View/EV2/EV2D/Source_Data_Fig_EV2D_HDX_MS_Uptake_Plots_AurA/chart_output015-031.png]

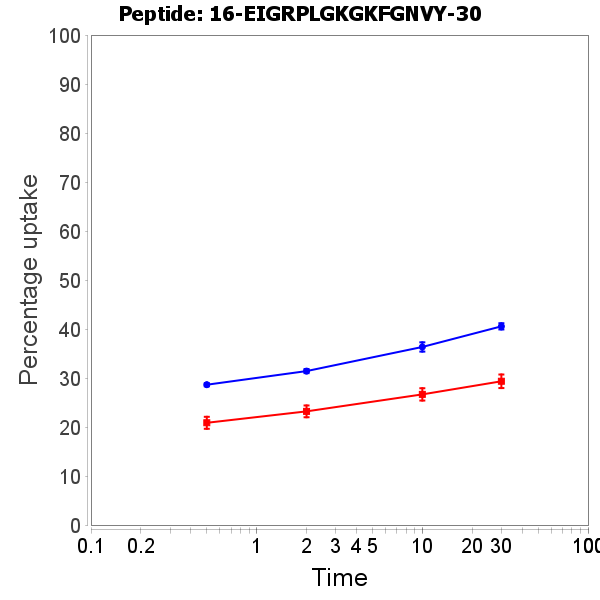

Supplement: Supplementary file 10 — EV and Appendix Figure Source Data [file 44318_2024_240_MOESM10_ESM.zip › Expanded View/EV2/EV2D/Source_Data_Fig_EV2D_HDX_MS_Uptake_Plots_AurA/chart_output016-030.png]

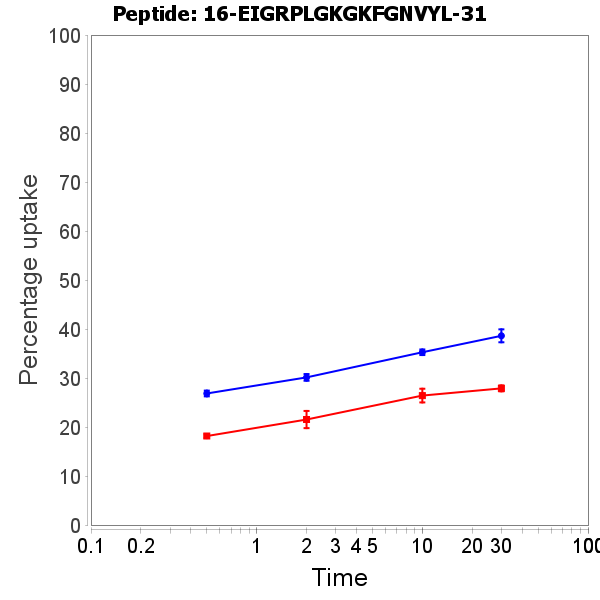

Supplement: Supplementary file 10 — EV and Appendix Figure Source Data [file 44318_2024_240_MOESM10_ESM.zip › Expanded View/EV2/EV2D/Source_Data_Fig_EV2D_HDX_MS_Uptake_Plots_AurA/chart_output016-031.png]

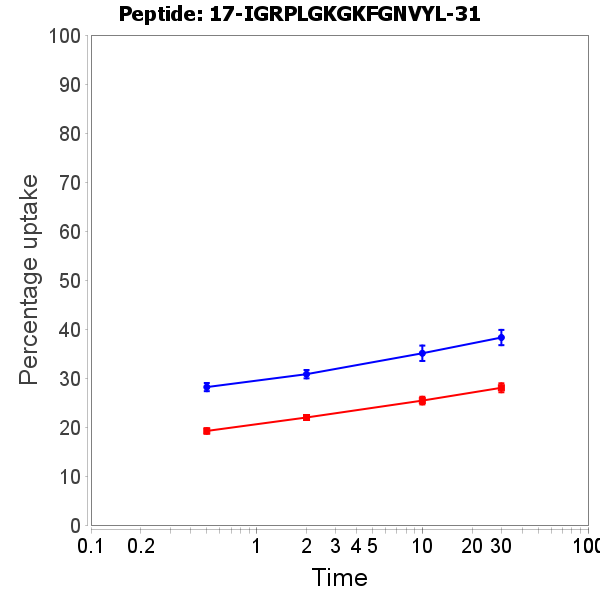

Supplement: Supplementary file 10 — EV and Appendix Figure Source Data [file 44318_2024_240_MOESM10_ESM.zip › Expanded View/EV2/EV2D/Source_Data_Fig_EV2D_HDX_MS_Uptake_Plots_AurA/chart_output017-031.png]

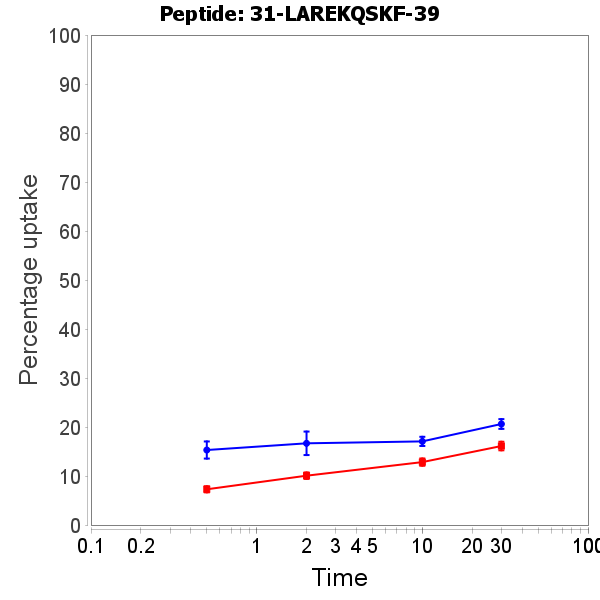

Supplement: Supplementary file 10 — EV and Appendix Figure Source Data [file 44318_2024_240_MOESM10_ESM.zip › Expanded View/EV2/EV2D/Source_Data_Fig_EV2D_HDX_MS_Uptake_Plots_AurA/chart_output031-039.png]

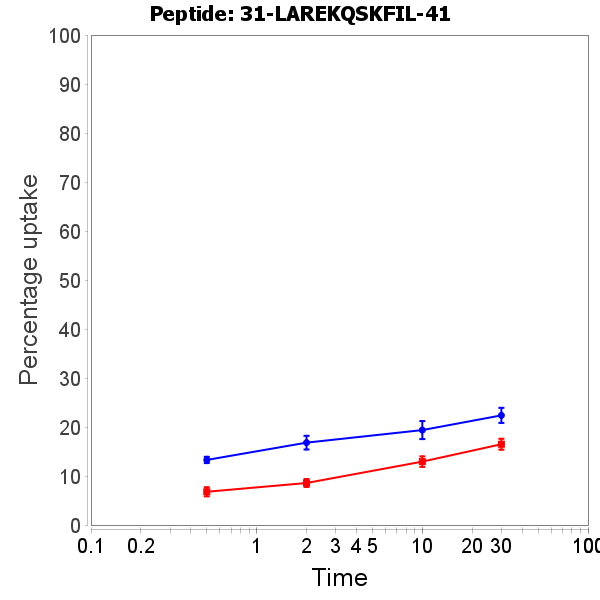

Supplement: Supplementary file 10 — EV and Appendix Figure Source Data [file 44318_2024_240_MOESM10_ESM.zip › Expanded View/EV2/EV2D/Source_Data_Fig_EV2D_HDX_MS_Uptake_Plots_AurA/chart_output031-041.png]

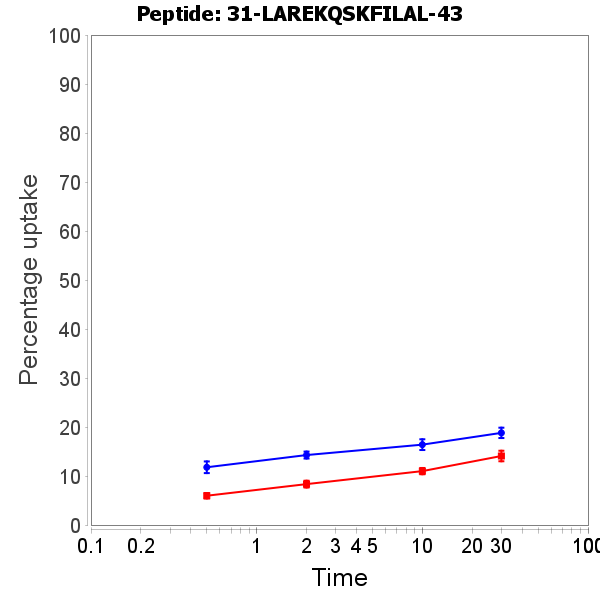

Supplement: Supplementary file 10 — EV and Appendix Figure Source Data [file 44318_2024_240_MOESM10_ESM.zip › Expanded View/EV2/EV2D/Source_Data_Fig_EV2D_HDX_MS_Uptake_Plots_AurA/chart_output031-043.png]

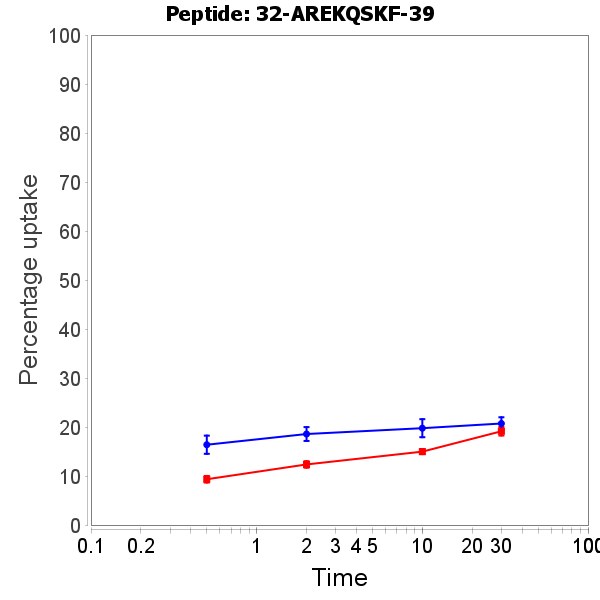

Supplement: Supplementary file 10 — EV and Appendix Figure Source Data [file 44318_2024_240_MOESM10_ESM.zip › Expanded View/EV2/EV2D/Source_Data_Fig_EV2D_HDX_MS_Uptake_Plots_AurA/chart_output032-039.png]

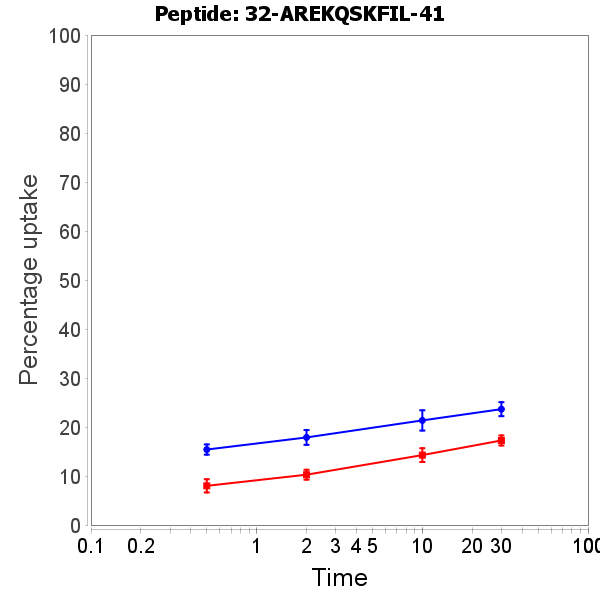

Supplement: Supplementary file 10 — EV and Appendix Figure Source Data [file 44318_2024_240_MOESM10_ESM.zip › Expanded View/EV2/EV2D/Source_Data_Fig_EV2D_HDX_MS_Uptake_Plots_AurA/chart_output032-041.png]

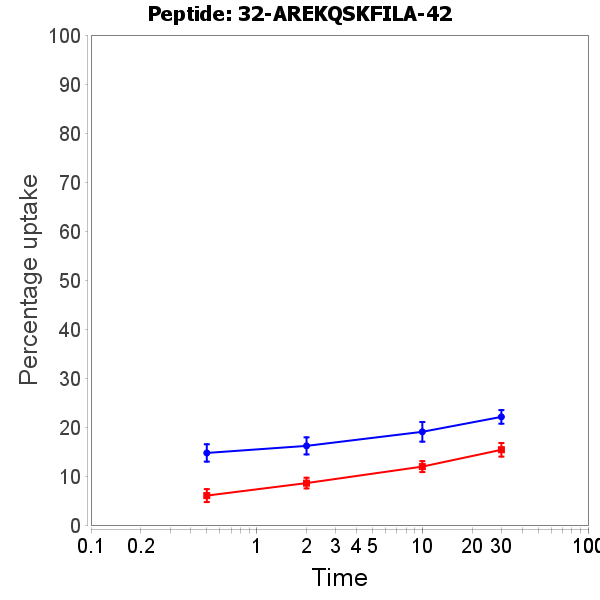

Supplement: Supplementary file 10 — EV and Appendix Figure Source Data [file 44318_2024_240_MOESM10_ESM.zip › Expanded View/EV2/EV2D/Source_Data_Fig_EV2D_HDX_MS_Uptake_Plots_AurA/chart_output032-042.png]

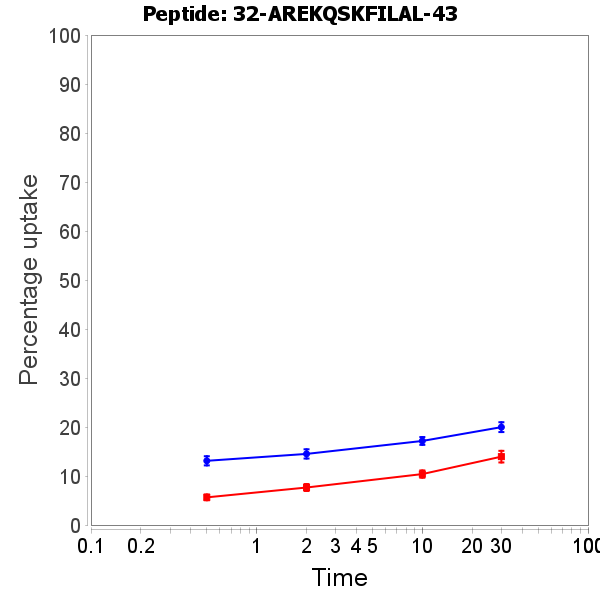

Supplement: Supplementary file 10 — EV and Appendix Figure Source Data [file 44318_2024_240_MOESM10_ESM.zip › Expanded View/EV2/EV2D/Source_Data_Fig_EV2D_HDX_MS_Uptake_Plots_AurA/chart_output032-043.png]

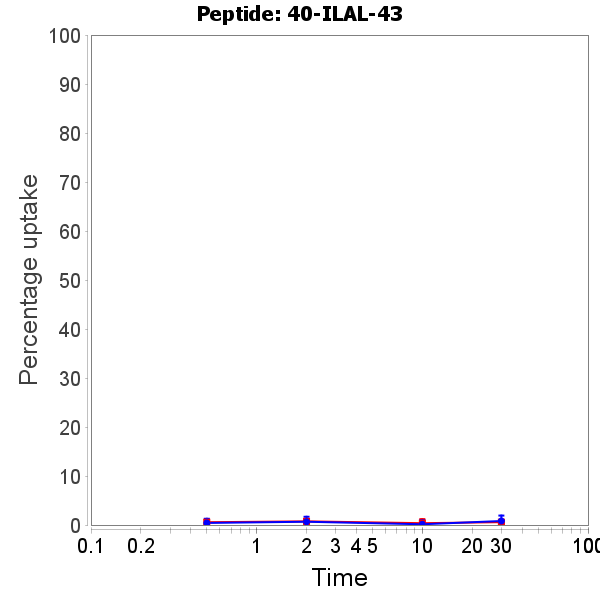

Supplement: Supplementary file 10 — EV and Appendix Figure Source Data [file 44318_2024_240_MOESM10_ESM.zip › Expanded View/EV2/EV2D/Source_Data_Fig_EV2D_HDX_MS_Uptake_Plots_AurA/chart_output040-043.png]

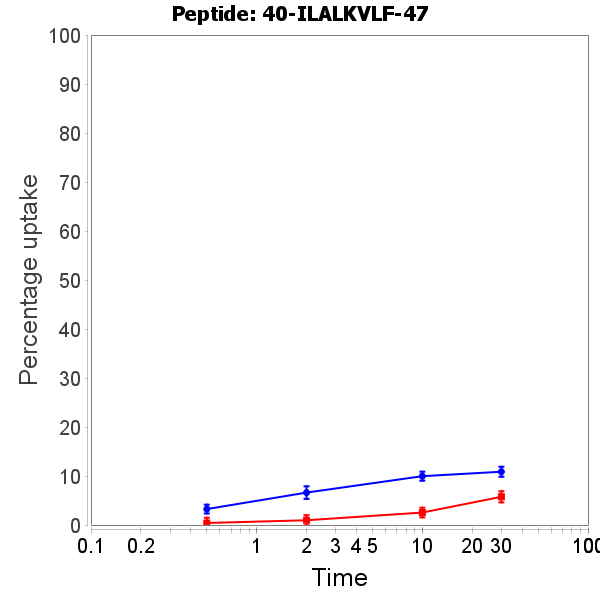

Supplement: Supplementary file 10 — EV and Appendix Figure Source Data [file 44318_2024_240_MOESM10_ESM.zip › Expanded View/EV2/EV2D/Source_Data_Fig_EV2D_HDX_MS_Uptake_Plots_AurA/chart_output040-047.png]

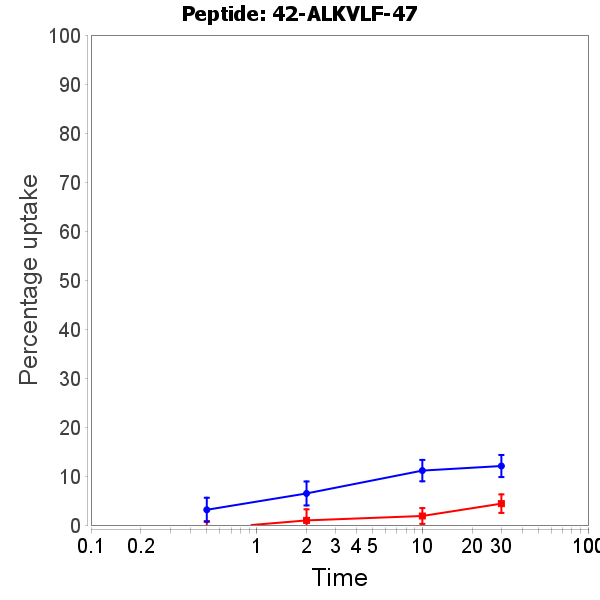

Supplement: Supplementary file 10 — EV and Appendix Figure Source Data [file 44318_2024_240_MOESM10_ESM.zip › Expanded View/EV2/EV2D/Source_Data_Fig_EV2D_HDX_MS_Uptake_Plots_AurA/chart_output042-047.png]

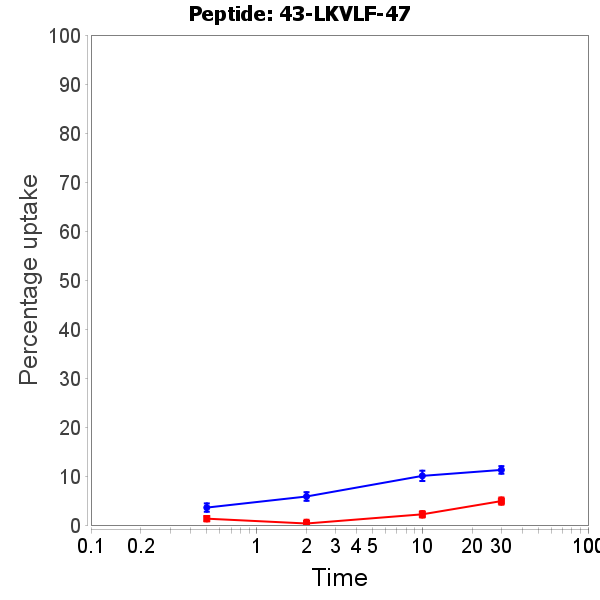

Supplement: Supplementary file 10 — EV and Appendix Figure Source Data [file 44318_2024_240_MOESM10_ESM.zip › Expanded View/EV2/EV2D/Source_Data_Fig_EV2D_HDX_MS_Uptake_Plots_AurA/chart_output043-047.png]

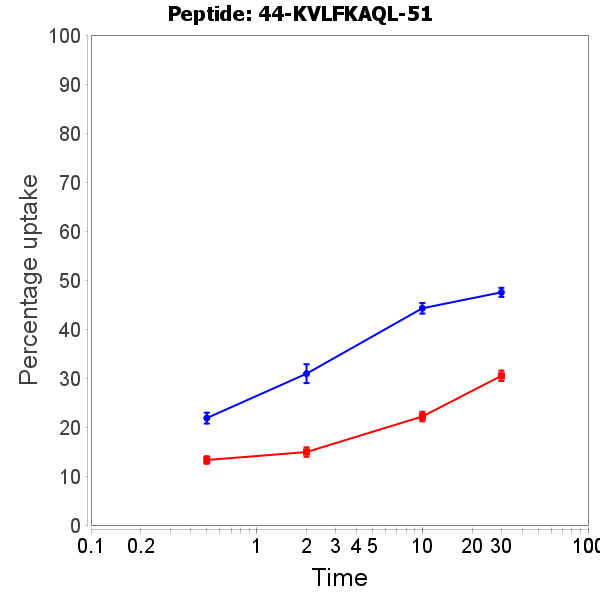

Supplement: Supplementary file 10 — EV and Appendix Figure Source Data [file 44318_2024_240_MOESM10_ESM.zip › Expanded View/EV2/EV2D/Source_Data_Fig_EV2D_HDX_MS_Uptake_Plots_AurA/chart_output044-051.png]

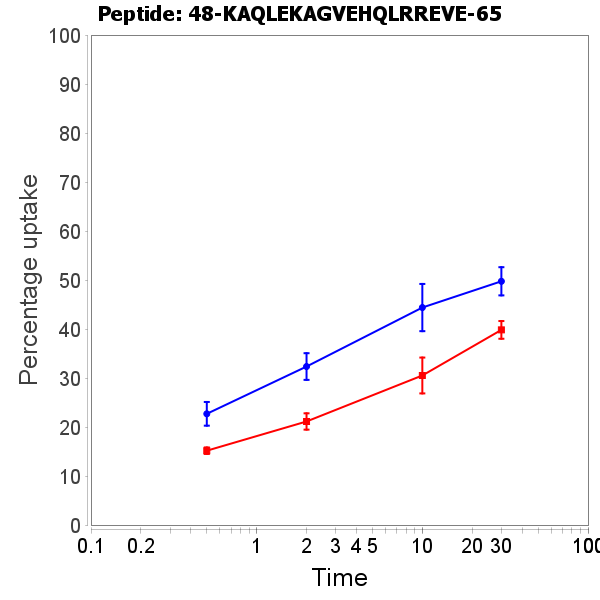

Supplement: Supplementary file 10 — EV and Appendix Figure Source Data [file 44318_2024_240_MOESM10_ESM.zip › Expanded View/EV2/EV2D/Source_Data_Fig_EV2D_HDX_MS_Uptake_Plots_AurA/chart_output048-065.png]

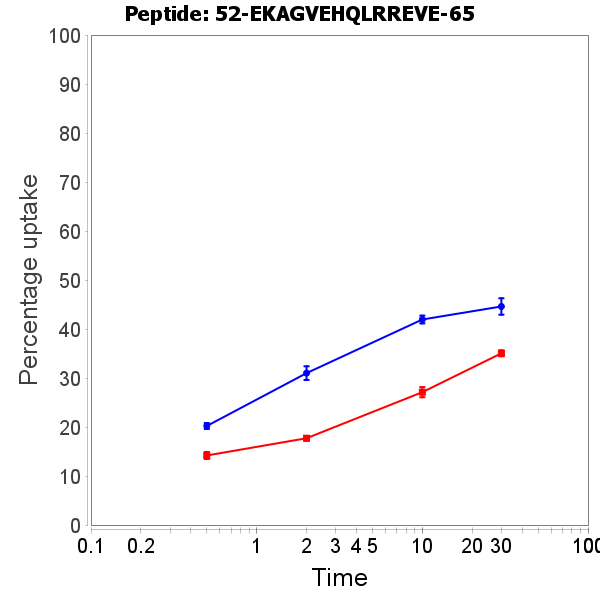

Supplement: Supplementary file 10 — EV and Appendix Figure Source Data [file 44318_2024_240_MOESM10_ESM.zip › Expanded View/EV2/EV2D/Source_Data_Fig_EV2D_HDX_MS_Uptake_Plots_AurA/chart_output052-065.png]

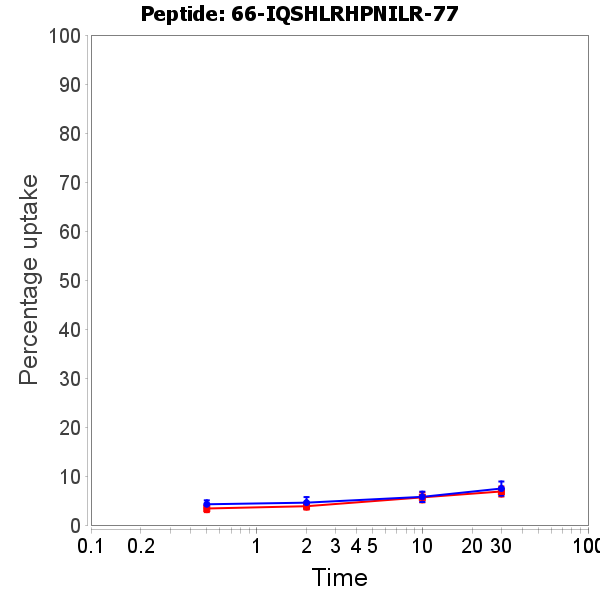

Supplement: Supplementary file 10 — EV and Appendix Figure Source Data [file 44318_2024_240_MOESM10_ESM.zip › Expanded View/EV2/EV2D/Source_Data_Fig_EV2D_HDX_MS_Uptake_Plots_AurA/chart_output066-077.png]

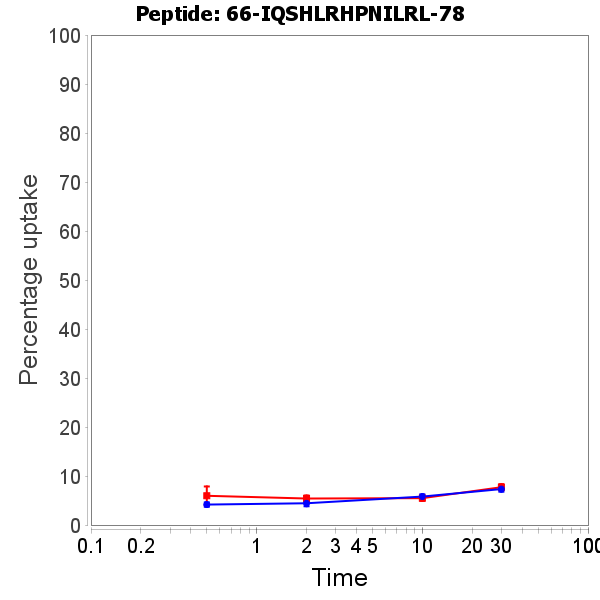

Supplement: Supplementary file 10 — EV and Appendix Figure Source Data [file 44318_2024_240_MOESM10_ESM.zip › Expanded View/EV2/EV2D/Source_Data_Fig_EV2D_HDX_MS_Uptake_Plots_AurA/chart_output066-078.png]

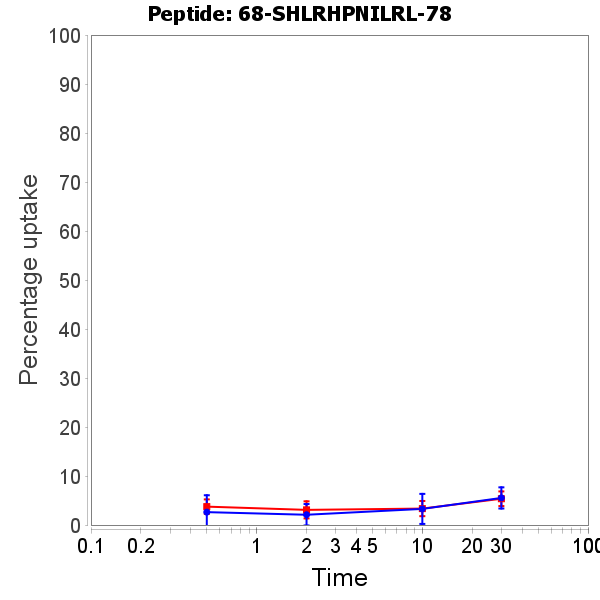

Supplement: Supplementary file 10 — EV and Appendix Figure Source Data [file 44318_2024_240_MOESM10_ESM.zip › Expanded View/EV2/EV2D/Source_Data_Fig_EV2D_HDX_MS_Uptake_Plots_AurA/chart_output068-078.png]

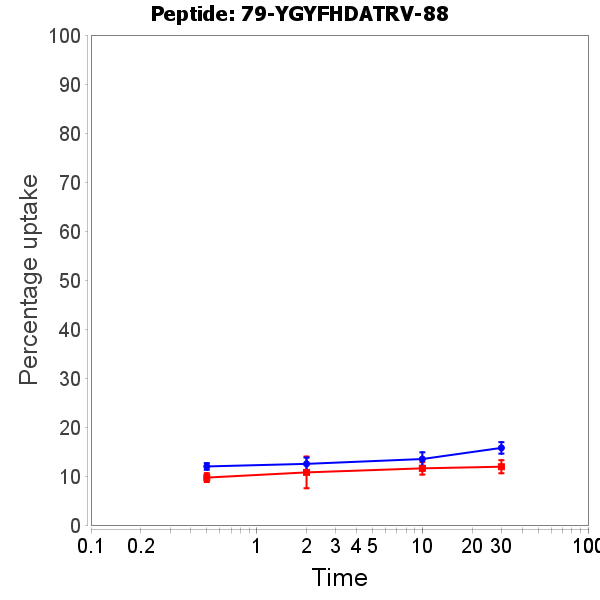

Supplement: Supplementary file 10 — EV and Appendix Figure Source Data [file 44318_2024_240_MOESM10_ESM.zip › Expanded View/EV2/EV2D/Source_Data_Fig_EV2D_HDX_MS_Uptake_Plots_AurA/chart_output079-088.png]

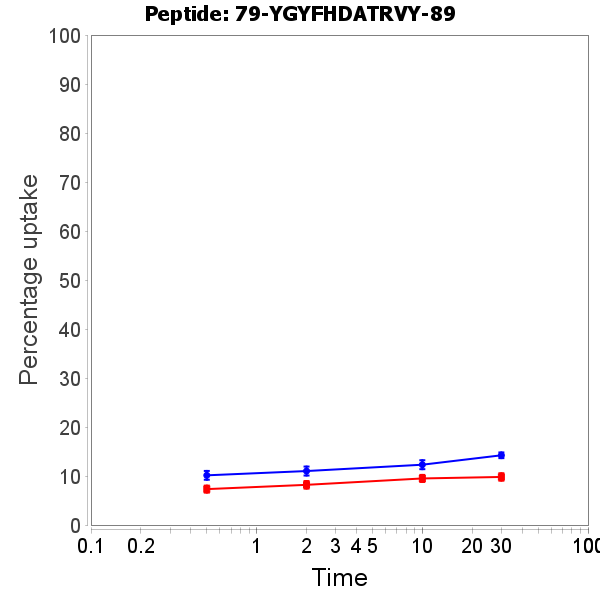

Supplement: Supplementary file 10 — EV and Appendix Figure Source Data [file 44318_2024_240_MOESM10_ESM.zip › Expanded View/EV2/EV2D/Source_Data_Fig_EV2D_HDX_MS_Uptake_Plots_AurA/chart_output079-089.png]

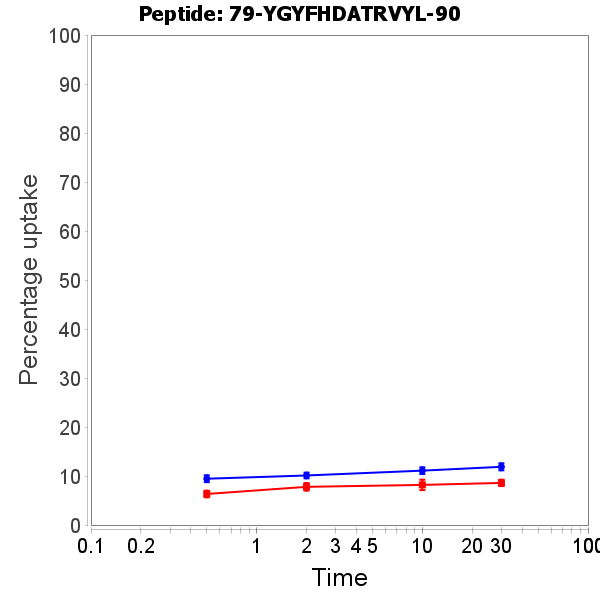

Supplement: Supplementary file 10 — EV and Appendix Figure Source Data [file 44318_2024_240_MOESM10_ESM.zip › Expanded View/EV2/EV2D/Source_Data_Fig_EV2D_HDX_MS_Uptake_Plots_AurA/chart_output079-090.png]

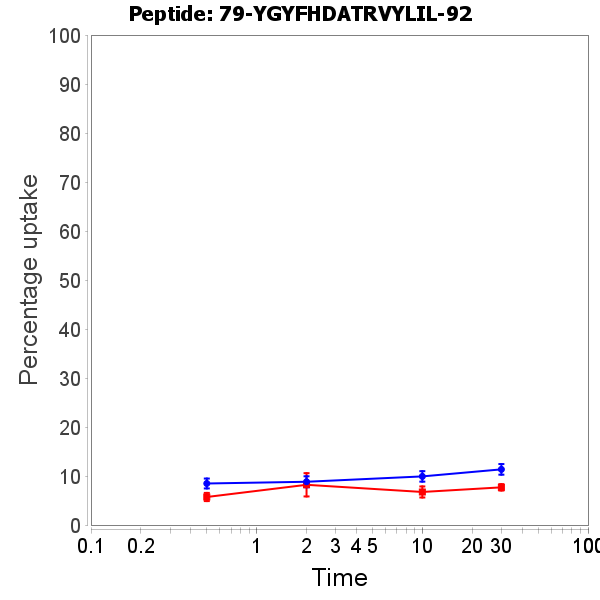

Supplement: Supplementary file 10 — EV and Appendix Figure Source Data [file 44318_2024_240_MOESM10_ESM.zip › Expanded View/EV2/EV2D/Source_Data_Fig_EV2D_HDX_MS_Uptake_Plots_AurA/chart_output079-092.png]

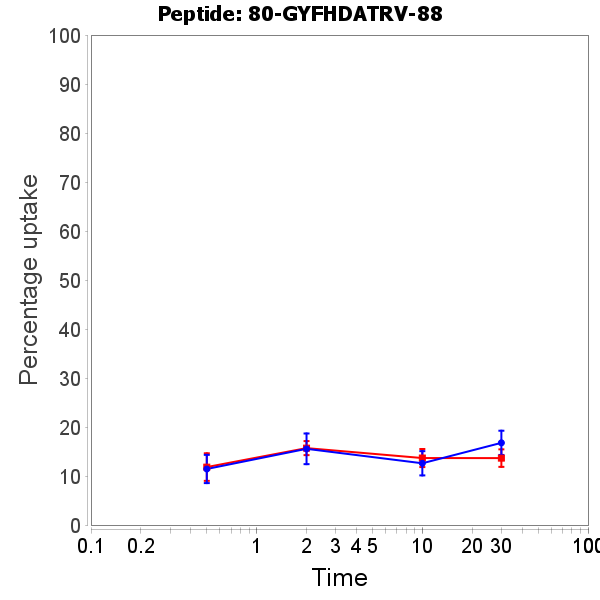

Supplement: Supplementary file 10 — EV and Appendix Figure Source Data [file 44318_2024_240_MOESM10_ESM.zip › Expanded View/EV2/EV2D/Source_Data_Fig_EV2D_HDX_MS_Uptake_Plots_AurA/chart_output080-088.png]

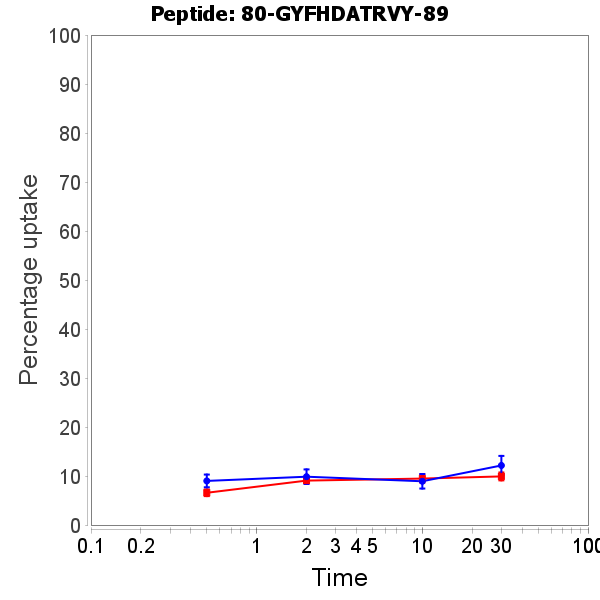

Supplement: Supplementary file 10 — EV and Appendix Figure Source Data [file 44318_2024_240_MOESM10_ESM.zip › Expanded View/EV2/EV2D/Source_Data_Fig_EV2D_HDX_MS_Uptake_Plots_AurA/chart_output080-089.png]

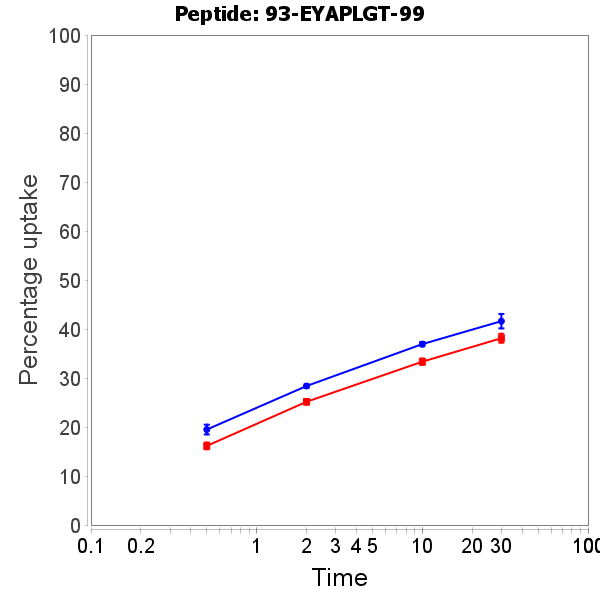

Supplement: Supplementary file 10 — EV and Appendix Figure Source Data [file 44318_2024_240_MOESM10_ESM.zip › Expanded View/EV2/EV2D/Source_Data_Fig_EV2D_HDX_MS_Uptake_Plots_AurA/chart_output093-099.png]

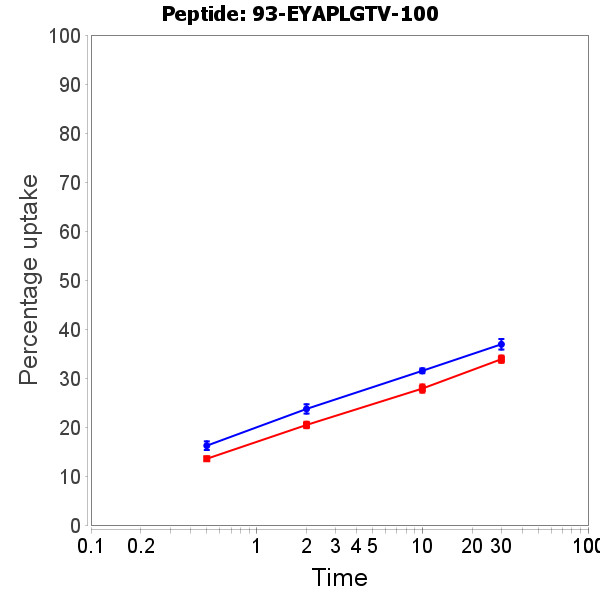

Supplement: Supplementary file 10 — EV and Appendix Figure Source Data [file 44318_2024_240_MOESM10_ESM.zip › Expanded View/EV2/EV2D/Source_Data_Fig_EV2D_HDX_MS_Uptake_Plots_AurA/chart_output093-100.png]

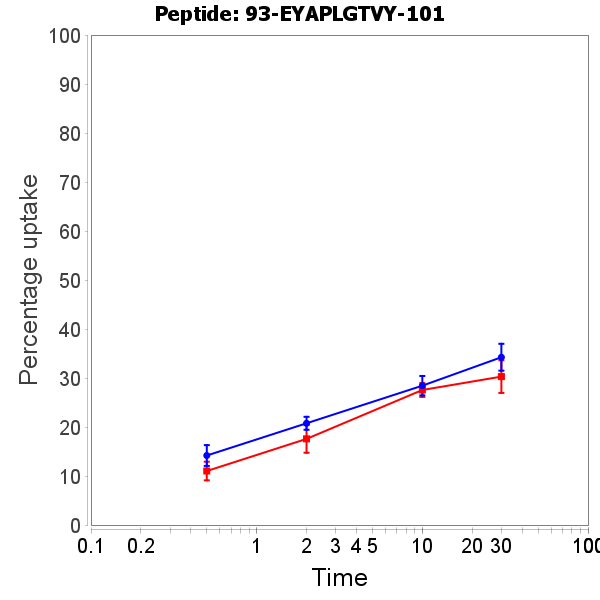

Supplement: Supplementary file 10 — EV and Appendix Figure Source Data [file 44318_2024_240_MOESM10_ESM.zip › Expanded View/EV2/EV2D/Source_Data_Fig_EV2D_HDX_MS_Uptake_Plots_AurA/chart_output093-101.png]

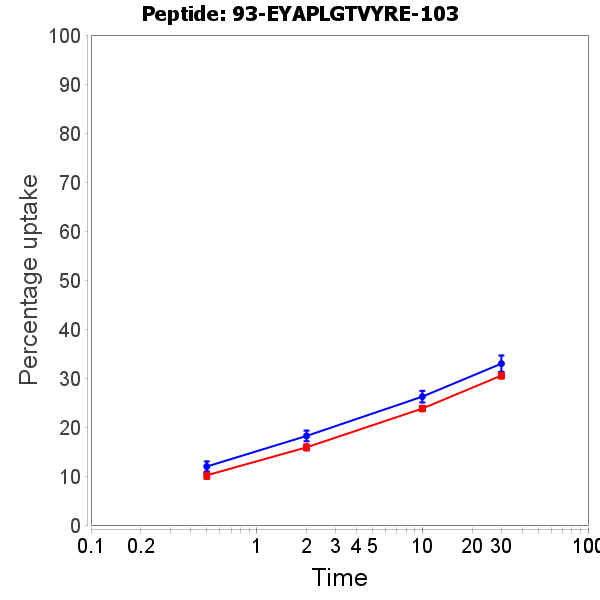

Supplement: Supplementary file 10 — EV and Appendix Figure Source Data [file 44318_2024_240_MOESM10_ESM.zip › Expanded View/EV2/EV2D/Source_Data_Fig_EV2D_HDX_MS_Uptake_Plots_AurA/chart_output093-103.png]

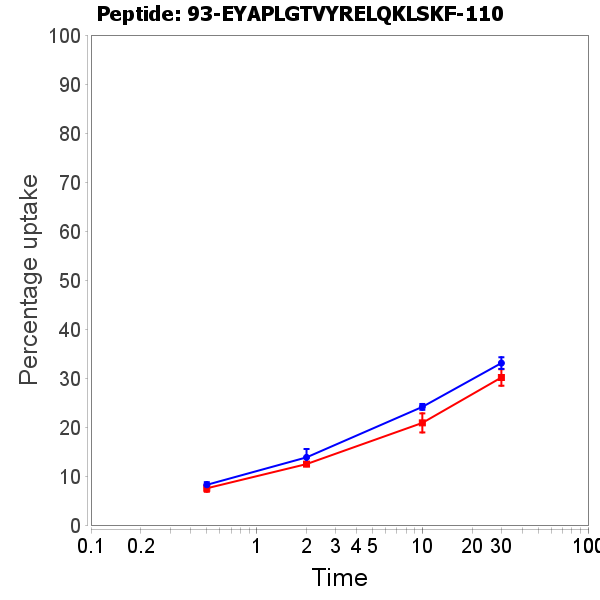

Supplement: Supplementary file 10 — EV and Appendix Figure Source Data [file 44318_2024_240_MOESM10_ESM.zip › Expanded View/EV2/EV2D/Source_Data_Fig_EV2D_HDX_MS_Uptake_Plots_AurA/chart_output093-110.png]

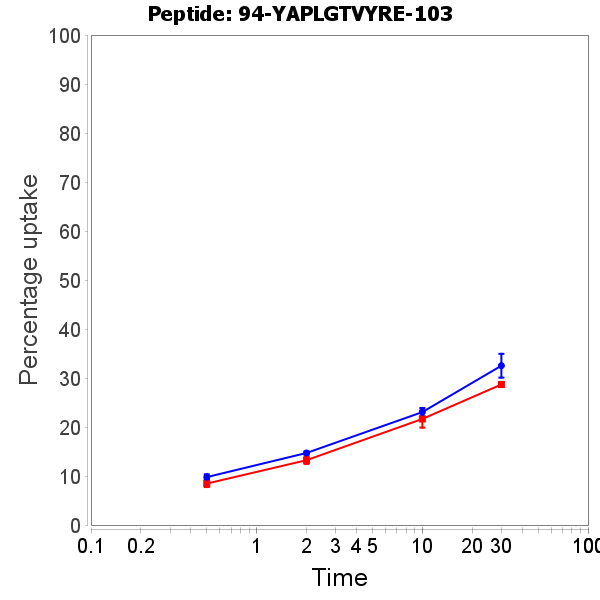

Supplement: Supplementary file 10 — EV and Appendix Figure Source Data [file 44318_2024_240_MOESM10_ESM.zip › Expanded View/EV2/EV2D/Source_Data_Fig_EV2D_HDX_MS_Uptake_Plots_AurA/chart_output094-103.png]

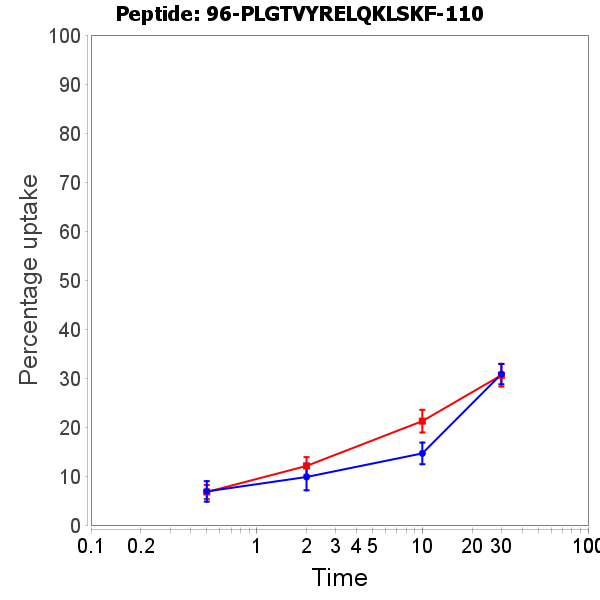

Supplement: Supplementary file 10 — EV and Appendix Figure Source Data [file 44318_2024_240_MOESM10_ESM.zip › Expanded View/EV2/EV2D/Source_Data_Fig_EV2D_HDX_MS_Uptake_Plots_AurA/chart_output096-110.png]

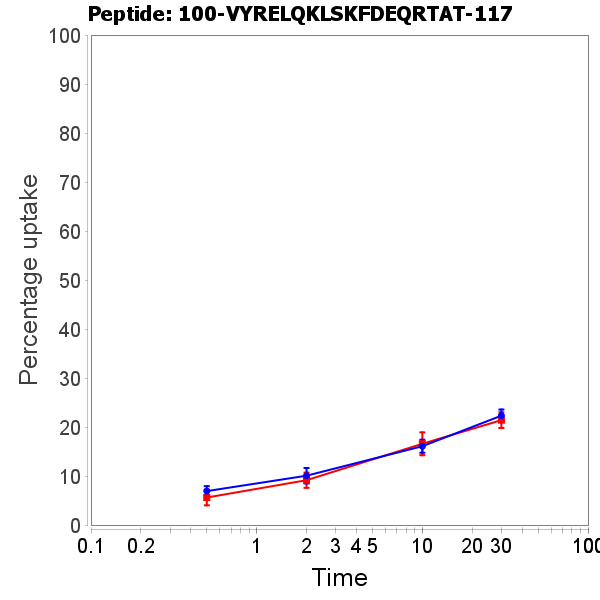

Supplement: Supplementary file 10 — EV and Appendix Figure Source Data [file 44318_2024_240_MOESM10_ESM.zip › Expanded View/EV2/EV2D/Source_Data_Fig_EV2D_HDX_MS_Uptake_Plots_AurA/chart_output100-117.png]

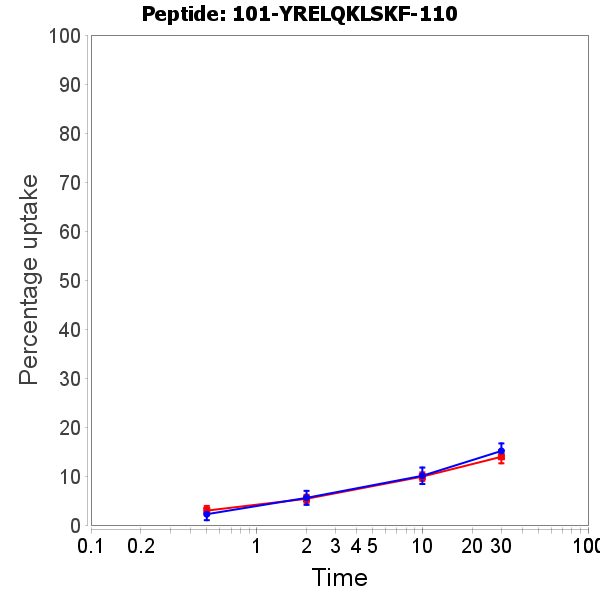

Supplement: Supplementary file 10 — EV and Appendix Figure Source Data [file 44318_2024_240_MOESM10_ESM.zip › Expanded View/EV2/EV2D/Source_Data_Fig_EV2D_HDX_MS_Uptake_Plots_AurA/chart_output101-110.png]

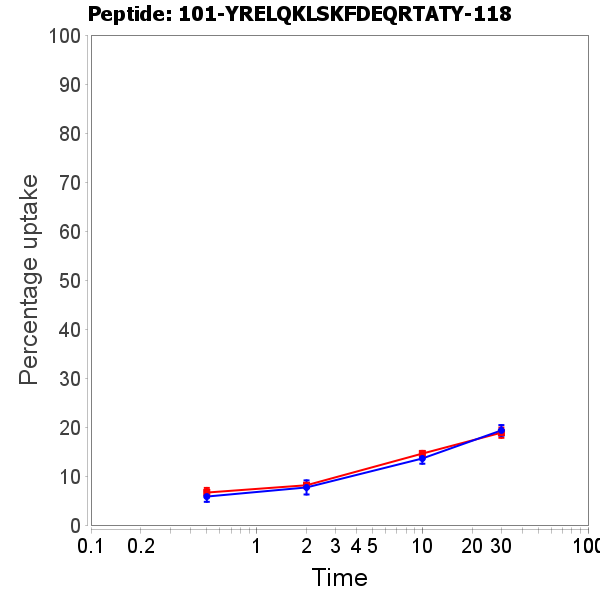

Supplement: Supplementary file 10 — EV and Appendix Figure Source Data [file 44318_2024_240_MOESM10_ESM.zip › Expanded View/EV2/EV2D/Source_Data_Fig_EV2D_HDX_MS_Uptake_Plots_AurA/chart_output101-118.png]

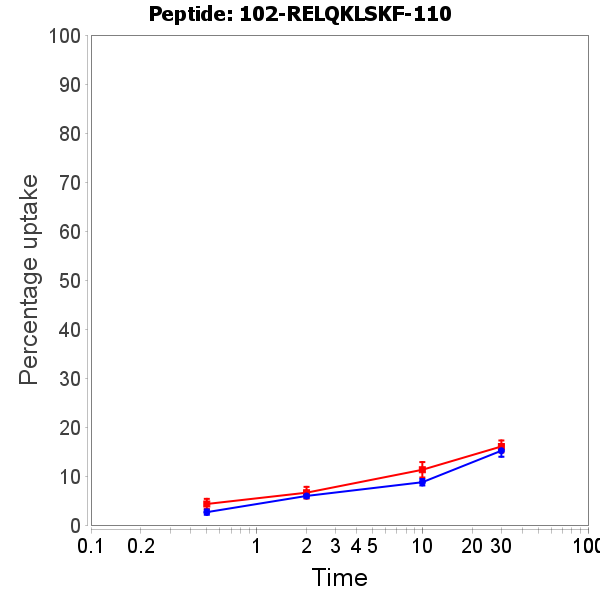

Supplement: Supplementary file 10 — EV and Appendix Figure Source Data [file 44318_2024_240_MOESM10_ESM.zip › Expanded View/EV2/EV2D/Source_Data_Fig_EV2D_HDX_MS_Uptake_Plots_AurA/chart_output102-110.png]

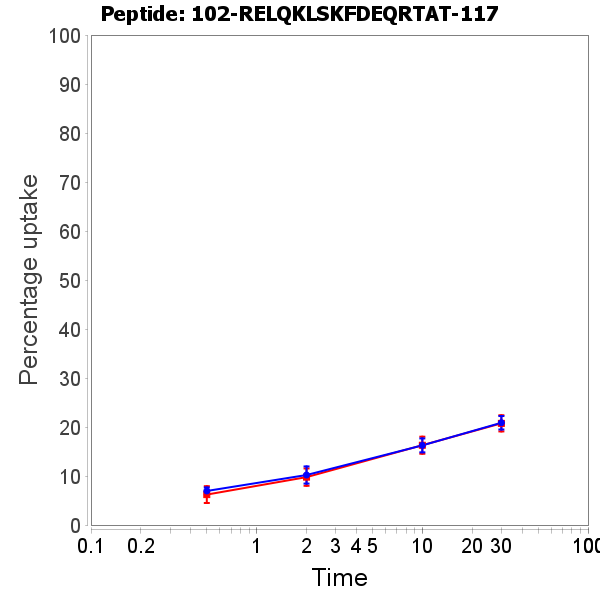

Supplement: Supplementary file 10 — EV and Appendix Figure Source Data [file 44318_2024_240_MOESM10_ESM.zip › Expanded View/EV2/EV2D/Source_Data_Fig_EV2D_HDX_MS_Uptake_Plots_AurA/chart_output102-117.png]

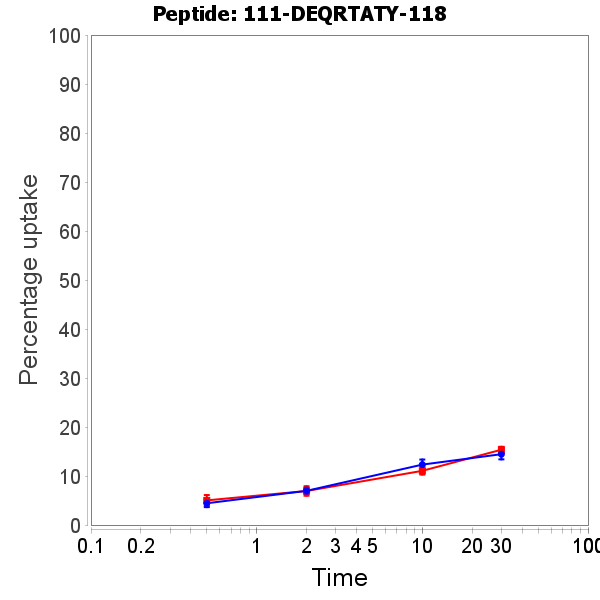

Supplement: Supplementary file 10 — EV and Appendix Figure Source Data [file 44318_2024_240_MOESM10_ESM.zip › Expanded View/EV2/EV2D/Source_Data_Fig_EV2D_HDX_MS_Uptake_Plots_AurA/chart_output111-118.png]

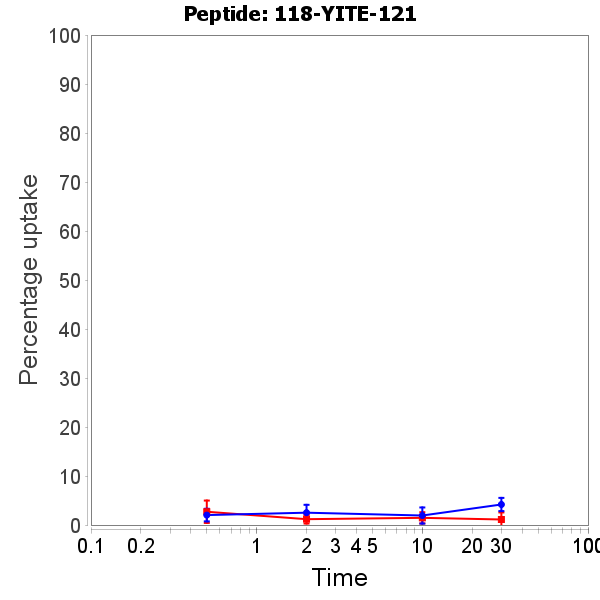

Supplement: Supplementary file 10 — EV and Appendix Figure Source Data [file 44318_2024_240_MOESM10_ESM.zip › Expanded View/EV2/EV2D/Source_Data_Fig_EV2D_HDX_MS_Uptake_Plots_AurA/chart_output118-121.png]

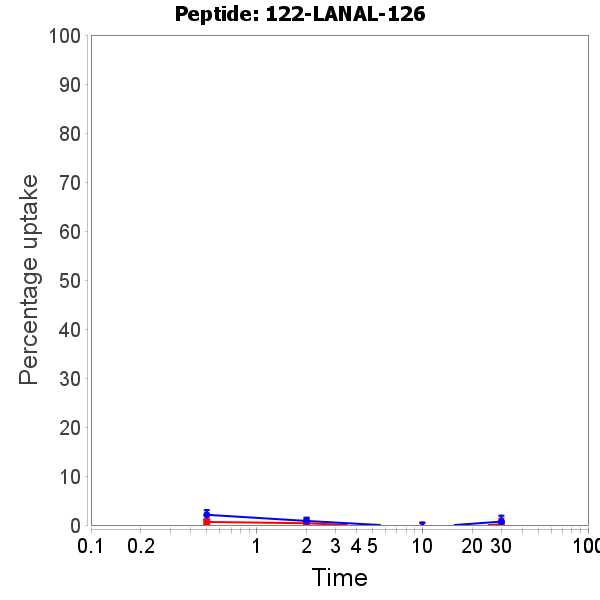

Supplement: Supplementary file 10 — EV and Appendix Figure Source Data [file 44318_2024_240_MOESM10_ESM.zip › Expanded View/EV2/EV2D/Source_Data_Fig_EV2D_HDX_MS_Uptake_Plots_AurA/chart_output122-126.png]

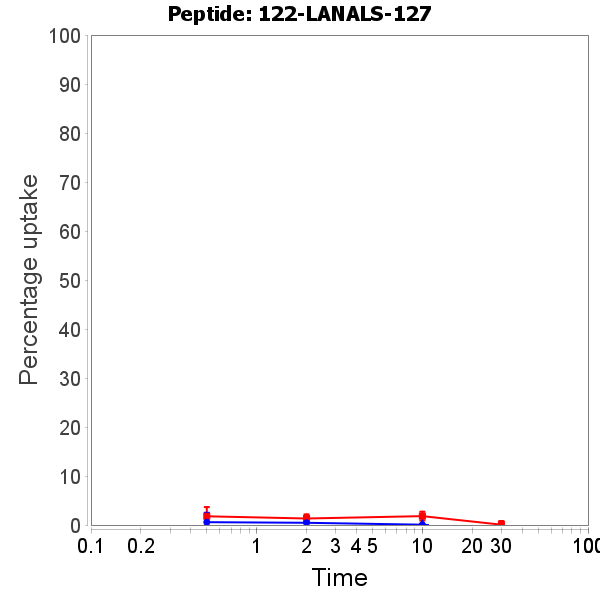

Supplement: Supplementary file 10 — EV and Appendix Figure Source Data [file 44318_2024_240_MOESM10_ESM.zip › Expanded View/EV2/EV2D/Source_Data_Fig_EV2D_HDX_MS_Uptake_Plots_AurA/chart_output122-127.png]

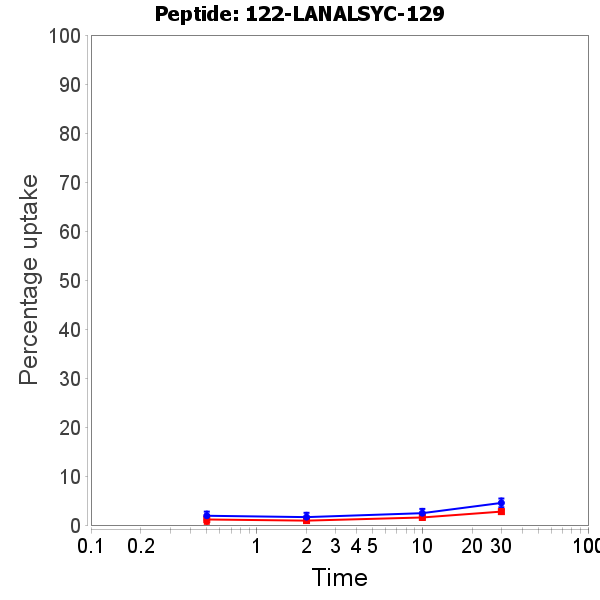

Supplement: Supplementary file 10 — EV and Appendix Figure Source Data [file 44318_2024_240_MOESM10_ESM.zip › Expanded View/EV2/EV2D/Source_Data_Fig_EV2D_HDX_MS_Uptake_Plots_AurA/chart_output122-129.png]

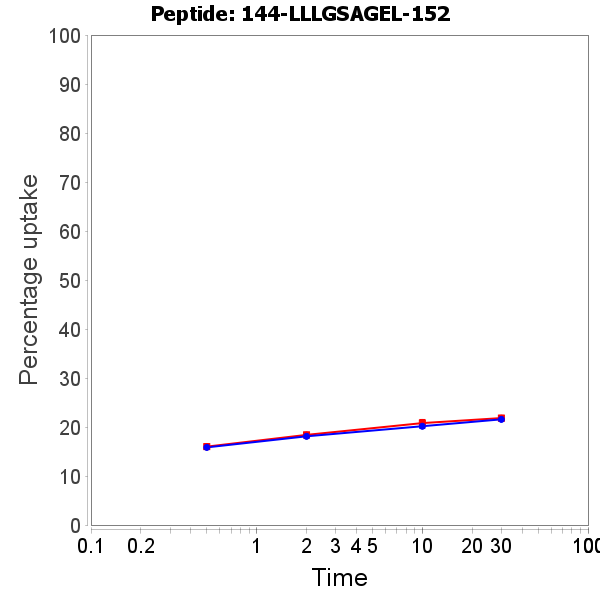

Supplement: Supplementary file 10 — EV and Appendix Figure Source Data [file 44318_2024_240_MOESM10_ESM.zip › Expanded View/EV2/EV2D/Source_Data_Fig_EV2D_HDX_MS_Uptake_Plots_AurA/chart_output144-152.png]

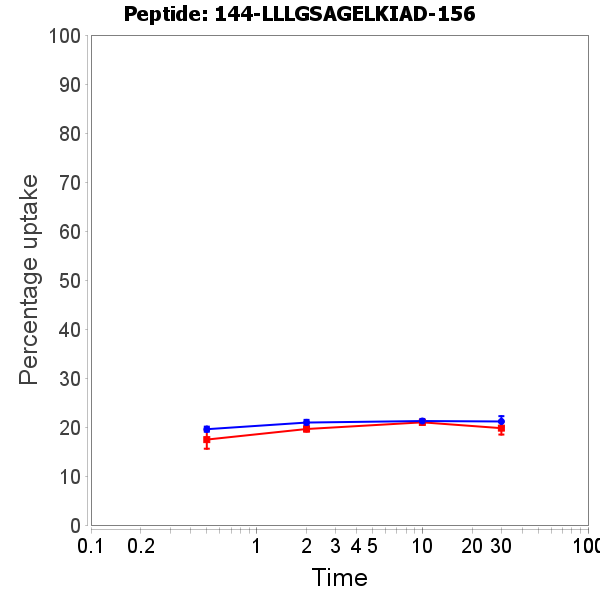

Supplement: Supplementary file 10 — EV and Appendix Figure Source Data [file 44318_2024_240_MOESM10_ESM.zip › Expanded View/EV2/EV2D/Source_Data_Fig_EV2D_HDX_MS_Uptake_Plots_AurA/chart_output144-156.png]

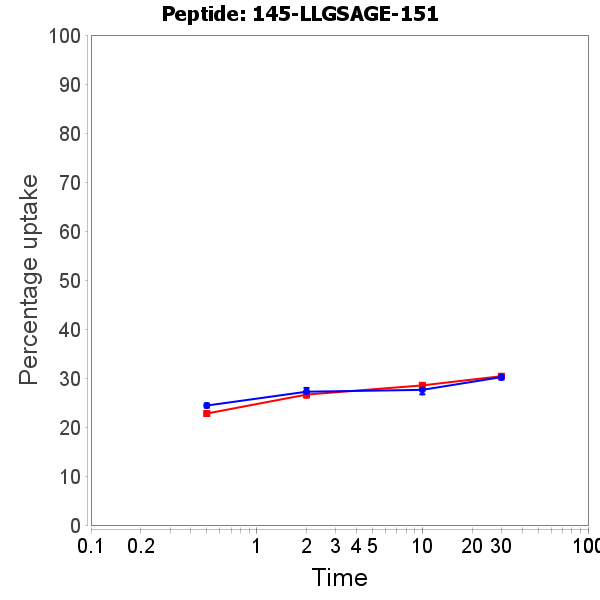

Supplement: Supplementary file 10 — EV and Appendix Figure Source Data [file 44318_2024_240_MOESM10_ESM.zip › Expanded View/EV2/EV2D/Source_Data_Fig_EV2D_HDX_MS_Uptake_Plots_AurA/chart_output145-151.png]

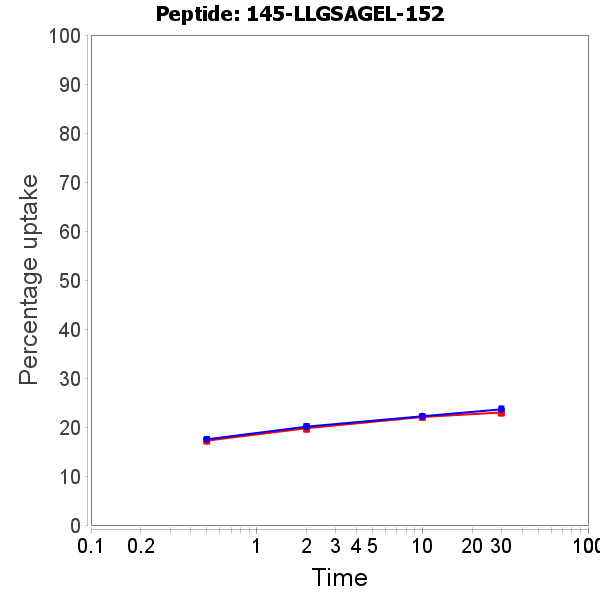

Supplement: Supplementary file 10 — EV and Appendix Figure Source Data [file 44318_2024_240_MOESM10_ESM.zip › Expanded View/EV2/EV2D/Source_Data_Fig_EV2D_HDX_MS_Uptake_Plots_AurA/chart_output145-152.png]

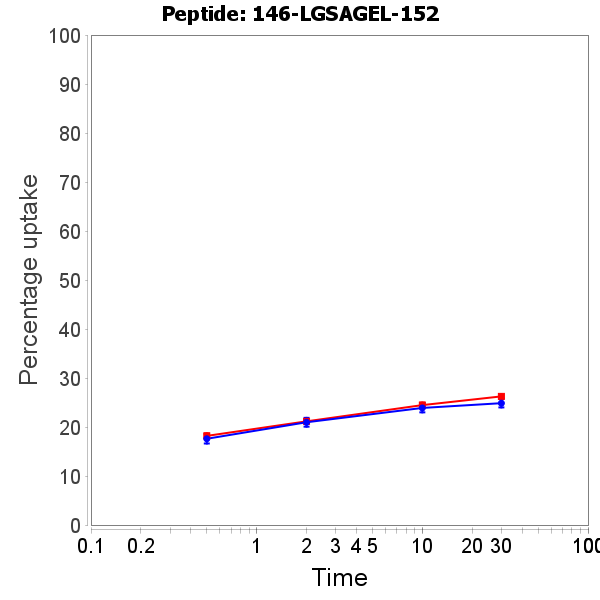

Supplement: Supplementary file 10 — EV and Appendix Figure Source Data [file 44318_2024_240_MOESM10_ESM.zip › Expanded View/EV2/EV2D/Source_Data_Fig_EV2D_HDX_MS_Uptake_Plots_AurA/chart_output146-152.png]

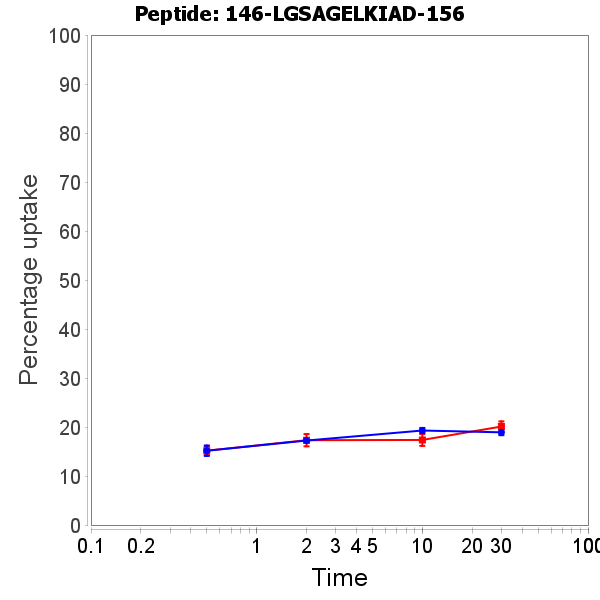

Supplement: Supplementary file 10 — EV and Appendix Figure Source Data [file 44318_2024_240_MOESM10_ESM.zip › Expanded View/EV2/EV2D/Source_Data_Fig_EV2D_HDX_MS_Uptake_Plots_AurA/chart_output146-156.png]

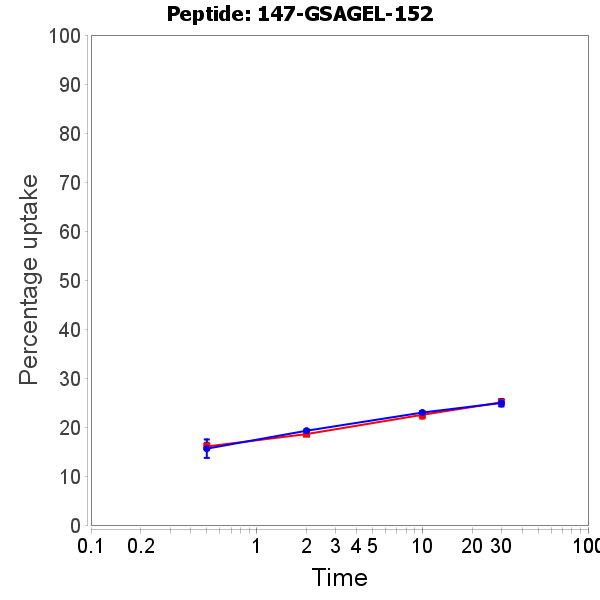

Supplement: Supplementary file 10 — EV and Appendix Figure Source Data [file 44318_2024_240_MOESM10_ESM.zip › Expanded View/EV2/EV2D/Source_Data_Fig_EV2D_HDX_MS_Uptake_Plots_AurA/chart_output147-152.png]

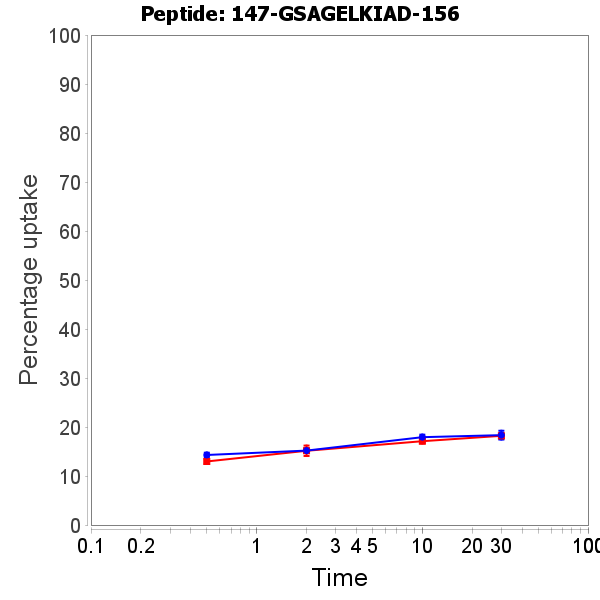

Supplement: Supplementary file 10 — EV and Appendix Figure Source Data [file 44318_2024_240_MOESM10_ESM.zip › Expanded View/EV2/EV2D/Source_Data_Fig_EV2D_HDX_MS_Uptake_Plots_AurA/chart_output147-156.png]

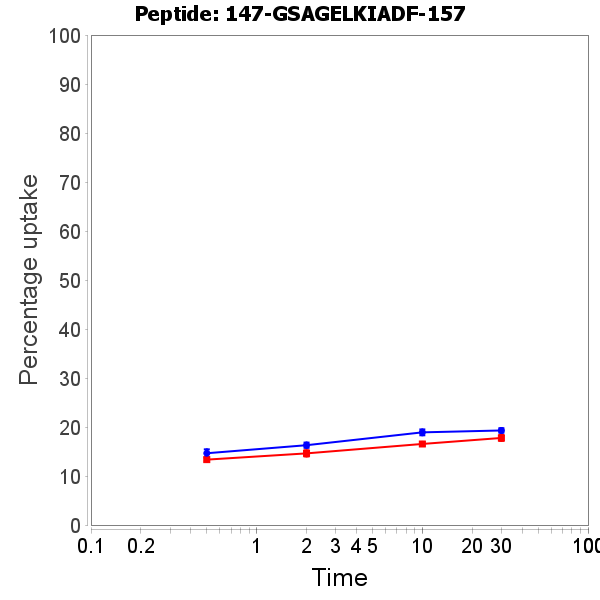

Supplement: Supplementary file 10 — EV and Appendix Figure Source Data [file 44318_2024_240_MOESM10_ESM.zip › Expanded View/EV2/EV2D/Source_Data_Fig_EV2D_HDX_MS_Uptake_Plots_AurA/chart_output147-157.png]

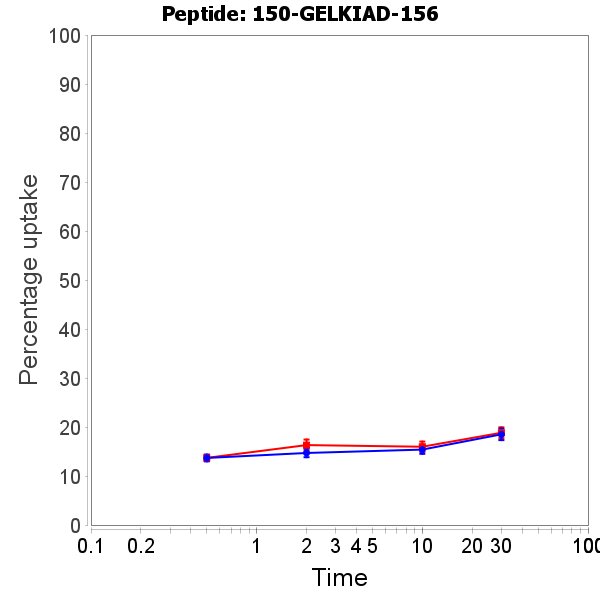

Supplement: Supplementary file 10 — EV and Appendix Figure Source Data [file 44318_2024_240_MOESM10_ESM.zip › Expanded View/EV2/EV2D/Source_Data_Fig_EV2D_HDX_MS_Uptake_Plots_AurA/chart_output150-156.png]

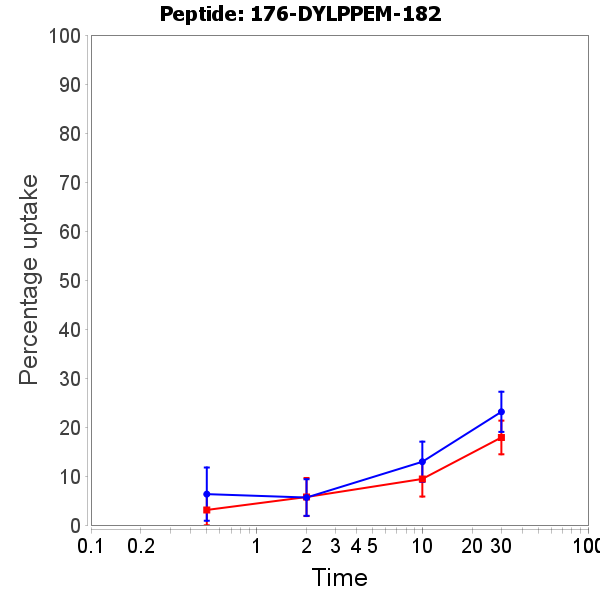

Supplement: Supplementary file 10 — EV and Appendix Figure Source Data [file 44318_2024_240_MOESM10_ESM.zip › Expanded View/EV2/EV2D/Source_Data_Fig_EV2D_HDX_MS_Uptake_Plots_AurA/chart_output176-182.png]

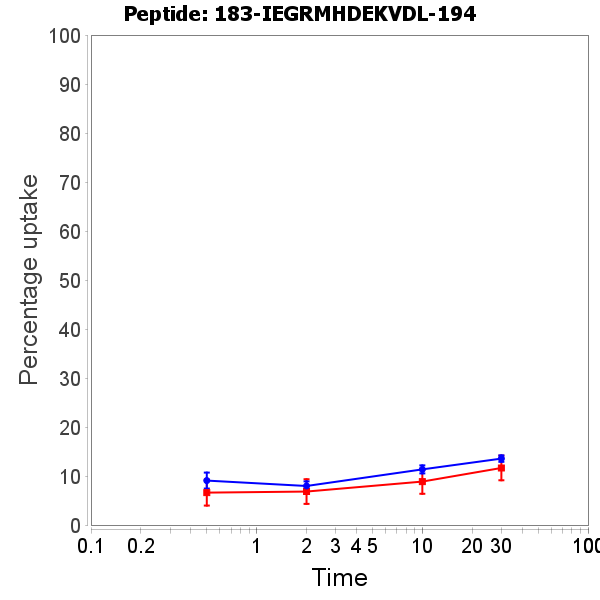

Supplement: Supplementary file 10 — EV and Appendix Figure Source Data [file 44318_2024_240_MOESM10_ESM.zip › Expanded View/EV2/EV2D/Source_Data_Fig_EV2D_HDX_MS_Uptake_Plots_AurA/chart_output183-194.png]
