# Supplementary material for: CEP192 localises mitotic Aurora-A activity by priming its interaction with TPX2
Source: EMBO J. 2024 Sep 26;43(22):5381–420. doi: 10.1038/s44318-024-00240-z (PMC11574021; doi:10.1038/s44318-024-00240-z)
Supplement: Supplementary file 11 — Expanded View Figures [file 44318_2024_240_MOESM11_ESM.pdf]

Expanded View Figures

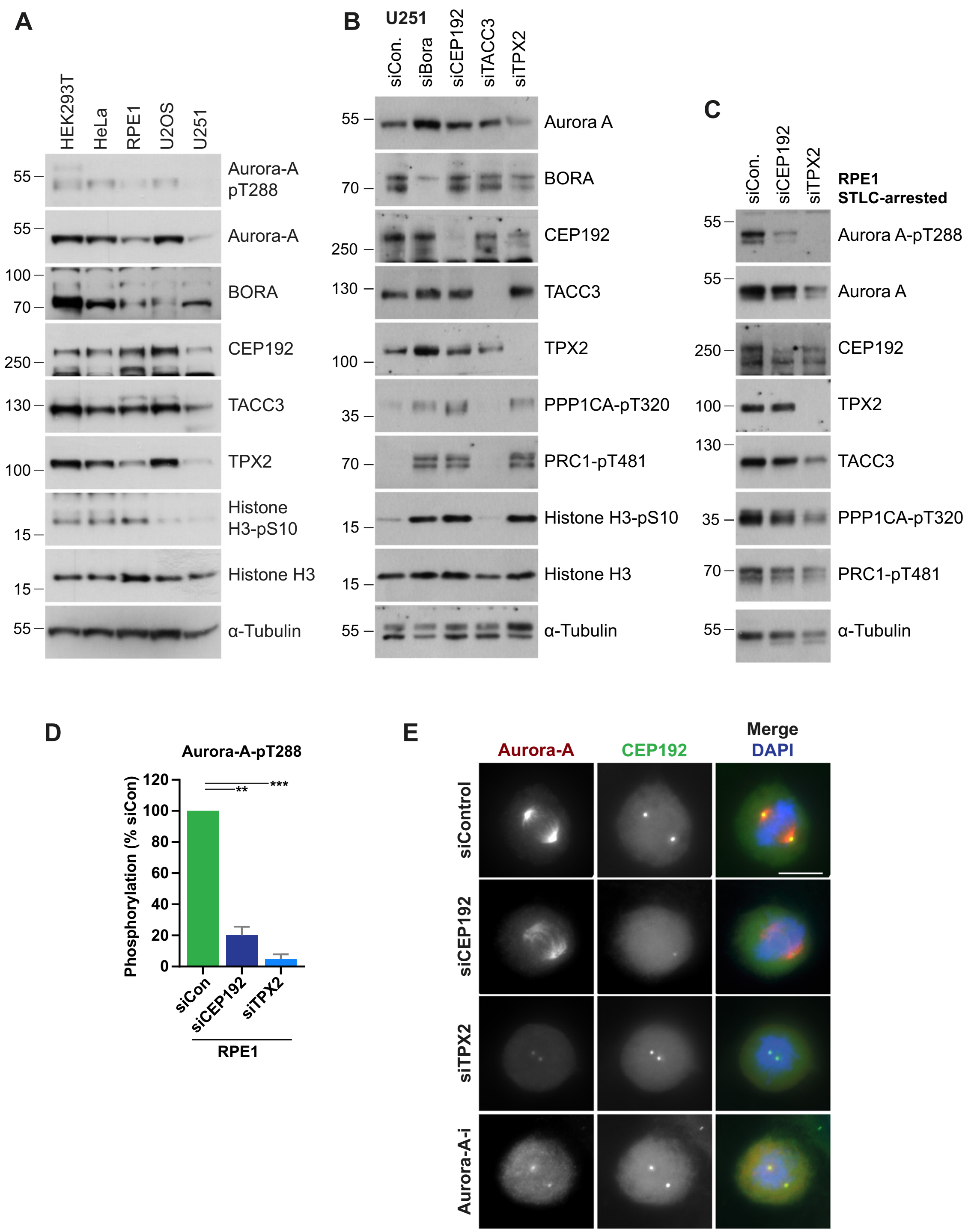

Figure EV1. Aurora-A colocalizes with CEP192 in the absence of TPX2 or Aurora-A activity.

(**A**) Western blot analysis of Aurora-A and its binding partners in a panel of asynchronous cell lines. (**B**) Western blot analysis of asynchronous U251 cells treated with the indicated siRNA (48 h). Antibodies against the mitotic phosphorylations PPP1CA-pT320, PRC1-pT481 and Histone H3-pS10 highlight the enrichment of mitotic cells following depletion of certain Aurora-A co-activators. (**C**) Western blot analysis of RPE1 cells treated with the indicated siRNA (48 h total) and arrested in mitosis with STLC (20 h). (**D**) Densitometric quantification of Aurora-A-pT288 signal from (**C**). Grey bars indicate mean ± S.D ($n = 3$ biological replicates). Exact p values (L-R): 0.0016, 0.0003. (**E**) Immunofluorescence images of U251 cells treated with siRNA as in (**B**), prior to 30 min incubation with DMSO control or Aurora-A inhibitor and then methanol fixation. Antibodies against Aurora-A and CEP192 are red and green in merged images, respectively, with DNA stained with DAPI (blue). Data information In (**D**), p values are denoted as follows: ***$p < 0.001$, **$p < 0.01$, (Welch's t-test). The scale bar in (**E**) represents 10 μm. Source data are available online for this figure.

© The Author(s)

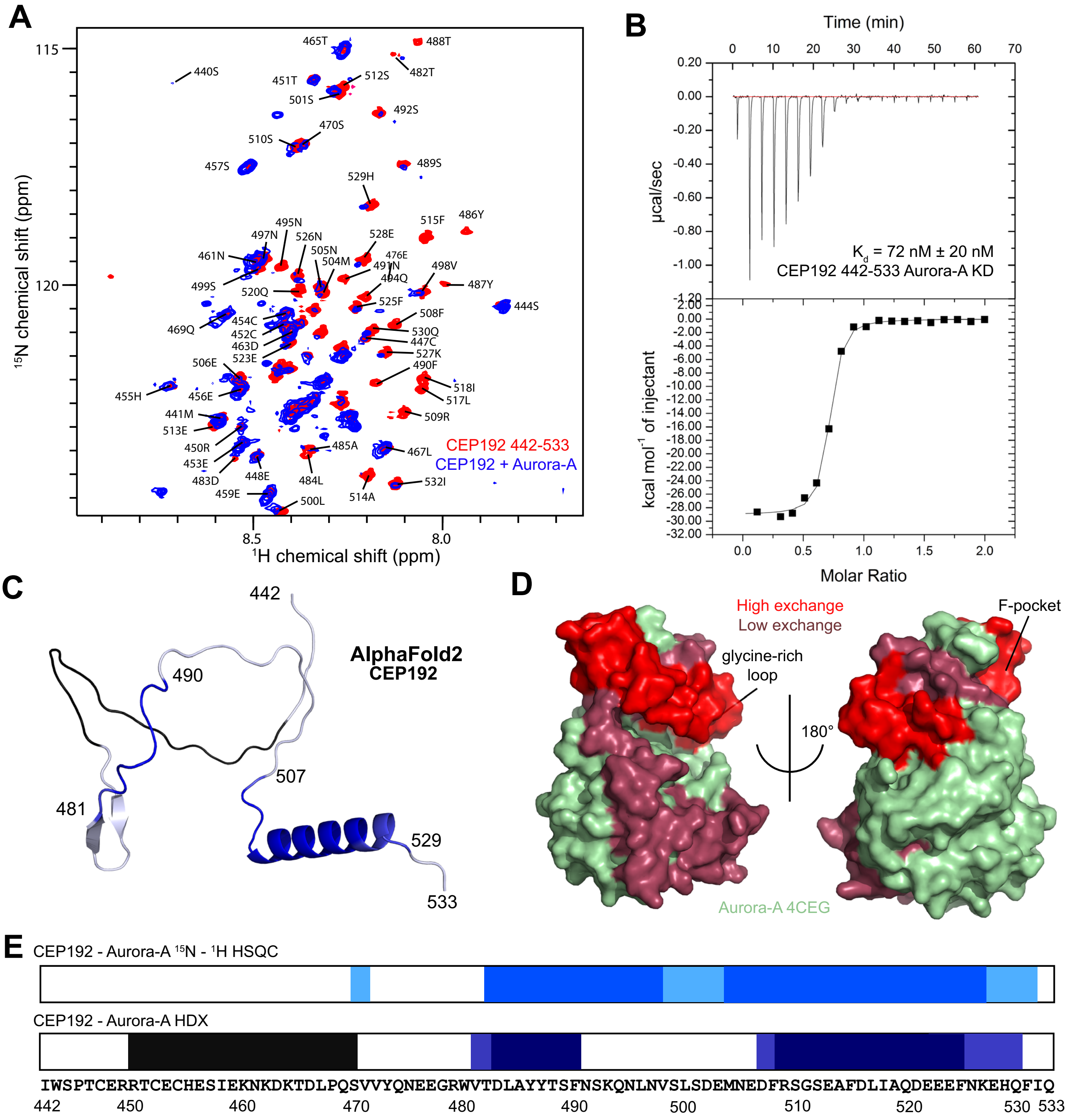
A
115
120
15N chemical shift (ppm)
8.5
8.0
1H chemical shift (ppm)
465T 488T 440S 451T 512S 482T 501S 492S 470S 510S 457S 489S 529H 495N 515F 486Y 497N 526N 528E 461N 505N 504M 476E 491N 498V 499S 494Q 487Y 520Q 525F 444S 454C 508F 469Q 452C 530Q 463D 447C 523E 527K 506E 490F 518I 455H 456E 517L 441M 509R 513E 485A 450R 467L 453E 448E 483D 484L 532I 459E 514A 500L
CEP192 442-533
CEP192 + Aurora-A
B
Time (min)
0 10 20 30 40 50 60 70
0.20 0.00 -0.20 -0.40 -0.60 -0.80 -1.00 -1.20
µcal/sec
Kd = 72 nM ± 20 nM
CEP192 442-533 Aurora-A KD
2.00 0.00 -2.00 -4.00 -6.00 -8.00 -10.00 -12.00 -14.00 -16.00 -18.00 -20.00 -22.00 -24.00 -26.00 -28.00 -30.00 -32.00
kcal mol-1 of injectant
0.0 0.5 1.0 1.5 2.0
Molar Ratio
C
442
490
481
507
529
533
AlphaFold2
CEP192
D
High exchange
Low exchange
glycine-rich
loop
180°
F-pocket
Aurora-A 4CEG
E
CEP192 - Aurora-A 15N - 1H HSQC
CEP192 - Aurora-A HDX
IWSPTCERRTCECHESIEKNKDKTDLPQSVVYQNEEGRWVTDLAYYTSFNSKQNLNVSLSDEMNEDFRSGSEAFDLIAQDEEEFNKEHQFIQ
442 450 460 470 480 490 500 510 520 530 533

 © The Author(s)

Figure EV2. CEP192 binds Aurora-A kinase domain with an extensive and high-affinity interaction.

(**A**) ^{1}H-^{15}N HSQC recorded on *Homo sapiens* CEP192 442–533 in the absence (red) and presence (blue) of Aurora-A 122–403 C290A C393A. A significant number of CEP192 peaks disappeared in the presence of Aurora-A, and following assignment, these residues were mapped between 470 to 533. (**B**) Isothermal titration calorimetry experiment showing titration of CEP192 442–533 into Aurora-A kinase domain (122–403 C290A C393A D274N). The measured K_d was 72 nM, with a molar ratio of 0.67 from two experimental repeats. (**C**) Mapping of the HDX-MS data from CEP192 442–533 in the presence of Aurora-A 122–403 C290A C393A onto an AlphaFold2 model of human CEP192 442–533. The dark blue shows regions with a greater than 10% difference in uptake, with lighter blue for differences in uptake between 5 and 10%. Regions where there was no change are shown in grey, with the region in black where no peptides were identified. (**D**) Mapping of the HDX-MS experiment as (**C**), mapped onto the surface of human Aurora-A^{CAKD} (PDB: 4CEG, shown in light green). The darker the red, the greater the difference in uptake when CEP192 468–533 is present. (**E**) Summary of the results of the interaction mapping data from NMR and HDX-MS on CEP192 442–533. The residues in the ^{1}H-^{15}N-HSQC with the highest peak loss are shown in the top section in blue, with dark blue indicating peaks that completely disappeared and light blue indicating peaks that decreased and shifted significantly. The protected residues in the CEP192 HDX-MS are shown in the bottom section in blue, with peptides where uptake differed by over 10% shown in dark blue and uptake differences between 5 and 10% shown in light blue. The region in black shows where no peptides were identified. Source data are available online for this figure.

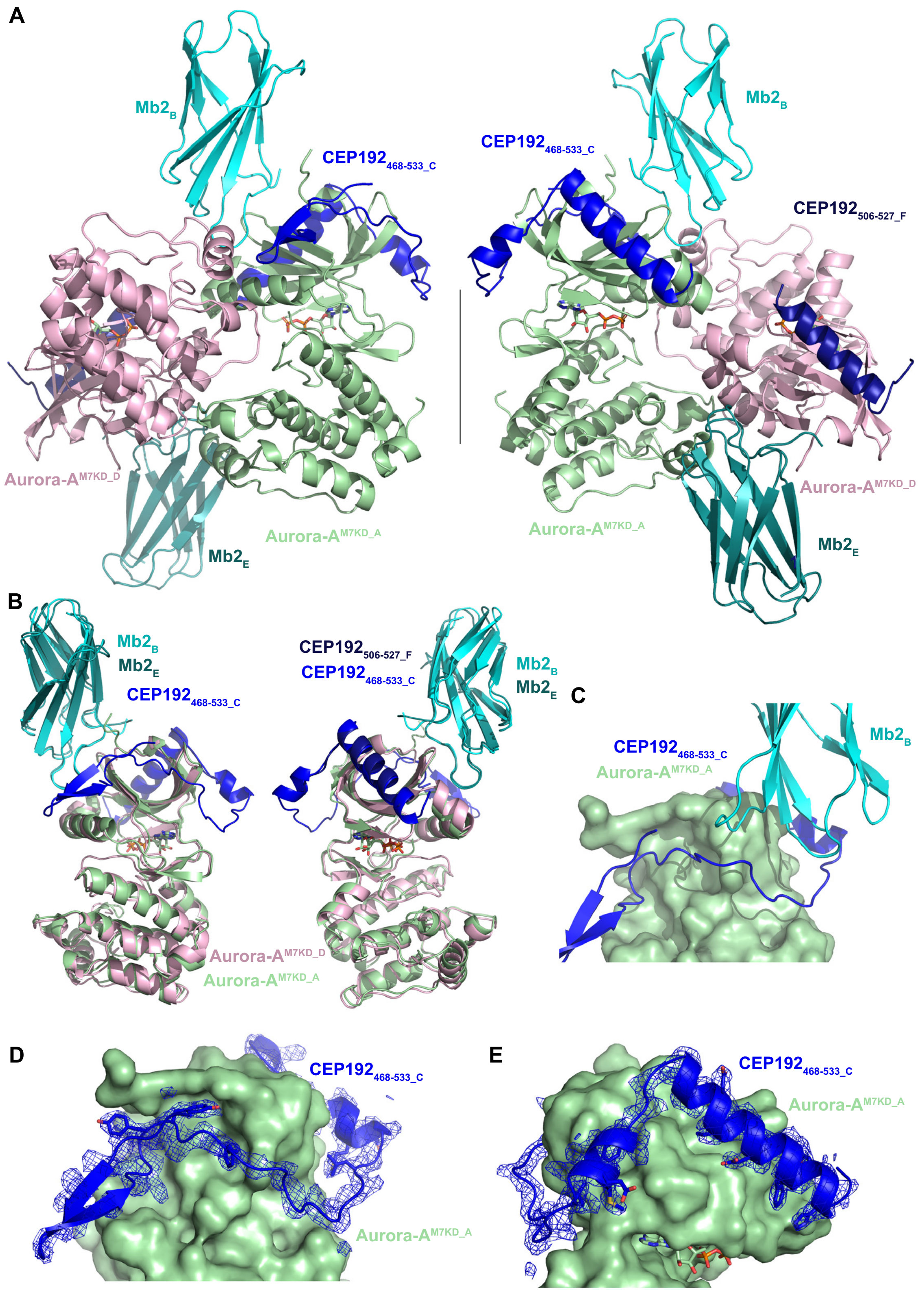
A
Mb2B
CEP192468-533_C
CEP192468-533_C
Mb2B
CEP192506-527_F
Aurora-AM7KD_D
Aurora-AM7KD_A
Mb2E
Aurora-AM7KD_A
Aurora-AM7KD_D
Mb2E
B
Mb2B
Mb2E
CEP192468-533_C
CEP192506-527_F
CEP192468-533_C
Mb2B
Mb2E
Aurora-AM7KD_D
Aurora-AM7KD_A
C
CEP192468-533_C
Aurora-AM7KD_A
Mb2B
D
CEP192468-533_C
Aurora-AM7KD_A
E
CEP192468-533_C
Aurora-AM7KD_A

 © The Author(s)

Figure EV3. Contents of the asymmetric unit of the crystal structure of Aurora-A^{M7KD} bound to CEP192$_{468\text{-}533}$ and the inhibitory monobody (Mb2).

(**A**) Cartoon representation of the asymmetric unit of the crystal structure of Aurora-A^{M7KD} bound to CEP19$_{468\text{-}533}$ and the inhibitory monobody. There are two copies of Aurora-A^{M7KD} (Chain A in light green, Chain D in light pink), 2 copies of the inhibitory monobody (Chains B and E in cyan) and 2 copies of CEP192$_{468\text{-}533}$ (Chains C and F in blue and dark blue). Only part of the CEP192 was visible in chain F (residues 506–526). (**B**) Overlay of the cartoon representations of the two Aurora-A^{M7KD} copies in the asymmetric unit. Chain A is shown in light green, with chain D in light pink. There are no significant differences between the two copies in the asymmetric unit. (**C**) Cartoon representation of the complex between CEP192$_{468\text{-}533_C}$ (dark blue) and Aurora-A^{M7KD_A} (light green) with a symmetry-related copy Mb2 (teal). The interface was analysed on PDBePISA, giving an interface score of 0.00 suggesting that this is merely a crystal contact and not a biologically relevant interface. (**D**) Representation of the electron density around CEP192$_{468\text{-}533}$ (dark blue) when bound to Aurora-A^{M7KD} (light green). The mesh represents a 2mFo-DFc map contoured at 1.2σ. (**E**) A second view of the electron density around CEP192$_{468\text{-}533}$ (dark blue) when bound to Aurora-A^{M7KD} (light green) to show the αS/αL region. The mesh represents a 2mFo-DFc map contoured at 1.2σ. Data information: Regions modelled for the different chains were 124–275 and 289–389 (chain A, Aurora-A^{M7KD}); 3–93 (chain B, Mb2); 468–531 (chain C, CEP192$_{468_533}$); 126–277 and 290–388 (chain D, Aurora-A^{M7KD}); 3–93 (chain E, Mb2); 506–527 (chain F, CEP192$_{468_533}$).

© The Author(s)

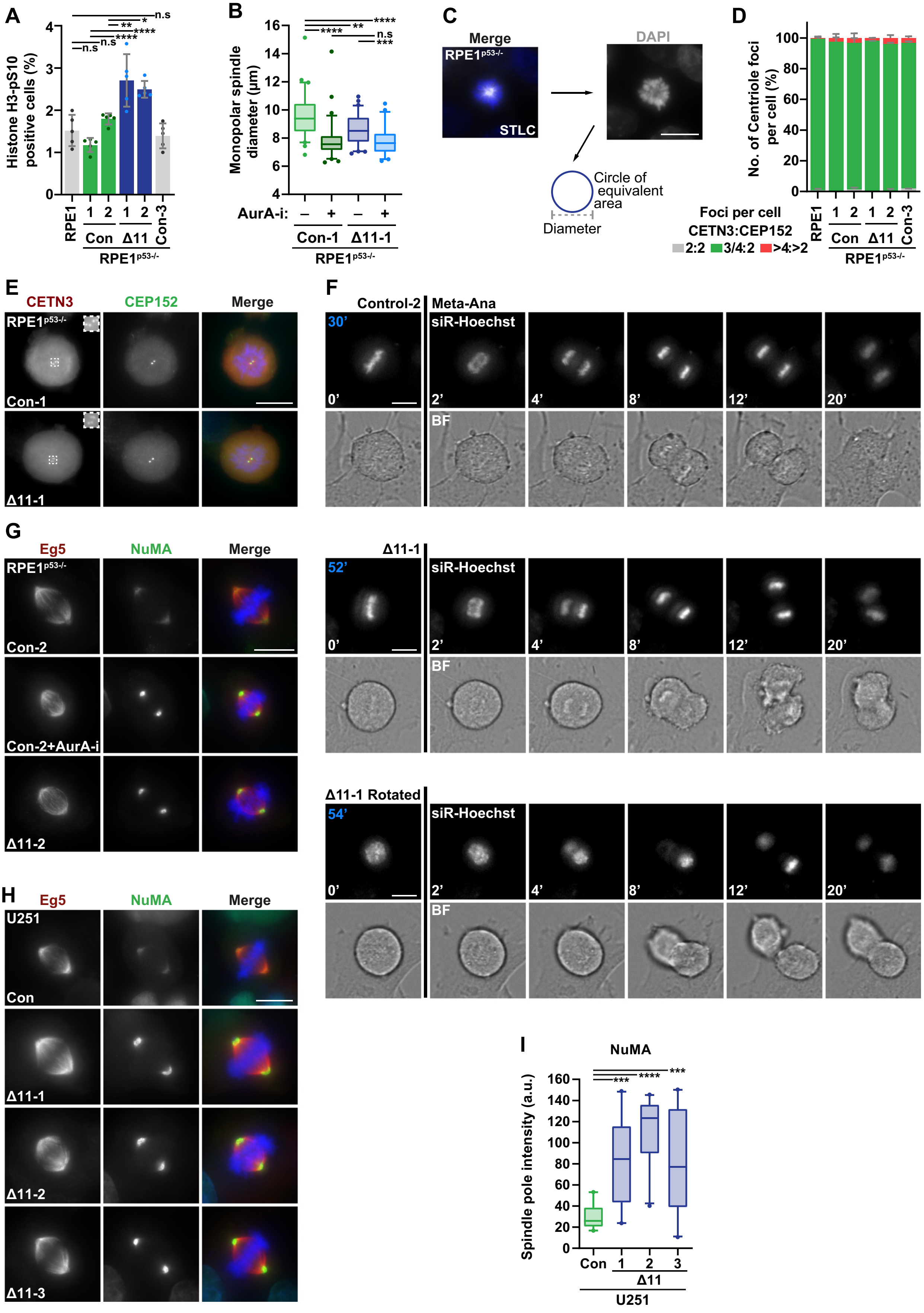
A
Histone H3-pS10 positive cells (%)
n.s
*
**
****
****
n.s
n.s
RPE1
1 2
Con
1 2
Δ11
Con-3
RPE1p53-/-
B
Monopolar spindle diameter (µm)
****
**
****
n.s
***
AurA-i:
Con-1
Δ11-1
RPE1p53-/-
C
Merge
RPE1p53-/-
STLC
DAPI
Circle of equivalent area
Diameter
D
No. of Centriole foci per cell (%)
Foci per cell
CETN3:CEP152
2:2
3/4:2
>4:>2
RPE1
Con
Δ11
Con-3
RPE1p53-/-
E
CETN3
CEP152
Merge
RPE1p53-/-
Con-1
Δ11-1
F
Control-2
Meta-Ana
30'
siR-Hoechst
0'
2'
4'
8'
12'
20'
BF
Δ11-1
52'
Δ11-1 Rotated
54'
G
Eg5
NuMA
Merge
RPE1p53-/-
Con-2
Con-2+AurA-i
Δ11-2
H
Eg5
NuMA
Merge
U251
Con
Δ11-1
Δ11-2
Δ11-3
I
NuMA
Spindle pole intensity (a.u.)
***
****
***
Con
1 2 3
Δ11
U251

 © The Author(s)

Figure EV4. The Aurora-A:CEP192 complex is required to establish proper mitotic spindle length and orientation.

(**A**) Bar chart showing the percentage of Histone H3-pS10 positive cells in multiple RPE1 and RPE1-derived cell lines ($n = 5$ biological replicates). Exact p values from (bottom-top): 0.4834, 0.6124, <0.0001, <0.0001, 0.0026, 0.0264, 0.6927. (**B**) Box plot of monopolar spindle diameter in control and Δ11-1 RPE1$^{p53-/-}$ cells treated with either DMSO control or Aurora-A inhibitor for 30 min prior to methanol fixation ($n = 3$, >15 cells/biological replicate). Exact p values from (bottom-top): 0.001, >0.9999, <0.0001, 0.0048, <0.0001. (**C**) Schematic detailing how monopolar spindle diameter, plotted in (**B**), was calculated based on each DAPI-stained DNA signal area (blue). (**D**) Bar chart showing the number of CETN3 and CEP152 foci in multiple STLC-arrested RPE1 and RPE1-derived cell lines ($n = 3$, ≥50 cells/biological replicate). (**E**) Representative immunofluorescence images of control and Δ11 RPE1$^{p53-/-}$ cells from (**D**). Antibodies against CETN3 and CEP152 are red and green in merged images, respectively, with DNA stained with DAPI (blue). Enlarged inserts centred on centriolar foci have sides 2 µm in length. (**F**) Representative images of control and Δ11 RPE1$^{p53-/-}$ progressing through anaphase, siR-Hoechst and brightfield (BF) channels are shown for each image. The black line indicates the metaphase-anaphase transition (Meta-Ana). Numbers in white indicate time relative to Meta-Ana, while numbers in blue indicate the time from NEBD-Ana (nuclear envelope breakdown to anaphase onset) for that cell (see also Fig. 7A-B). For Δ11-1 rotated, note how one of the daughter cells moves out of focus, indicating an out-of-plane division. (**G**, **H**) Immunofluorescence images of methanol fixed control and Δ11 (**G**) RPE1$^{p53-/-}$ and (**H**) U251 cells. Antibodies against Eg5 and NuMA are red and green in merged images, respectively, with DNA stained with DAPI (blue). Quantification of (**G**) is found in Fig. 7I, J. (**I**) Box plot of NuMA spindle pole signal intensity in U251 cells, with representative images shown in (**H**) ($n = 2$, ≥10 cells/biological replicate). Exact p values (L-R): <0.0001, <0.0001, <0.0001. Data information In (**A**) and (**D**) RPE1 indicates the parental p53$^{+/+}$ line from which other clones were derived. Con-3 is an additional RPE1$^{p53-/-}$ control cell line generated previously within the lab. Grey bars in (**A**) and (**D**) indicate mean ± S.D. Box plots in (**B**) and (**I**) indicate the median and interquartile ranges (25th–75th percentile) with coloured whiskers representing 5th–95th percentile ranges. p values are denoted as follows: ****$p < 0.0001$, ***$p < 0.001$, **$p < 0.01$, *$p < 0.05$, n.s not significant (**A** ANOVA, **B**, **I** Mann–Whitney test). Scale bars in (**C**) and (**E–H**) represent 10 µm. Source data are available online for this figure.

© The Author(s)

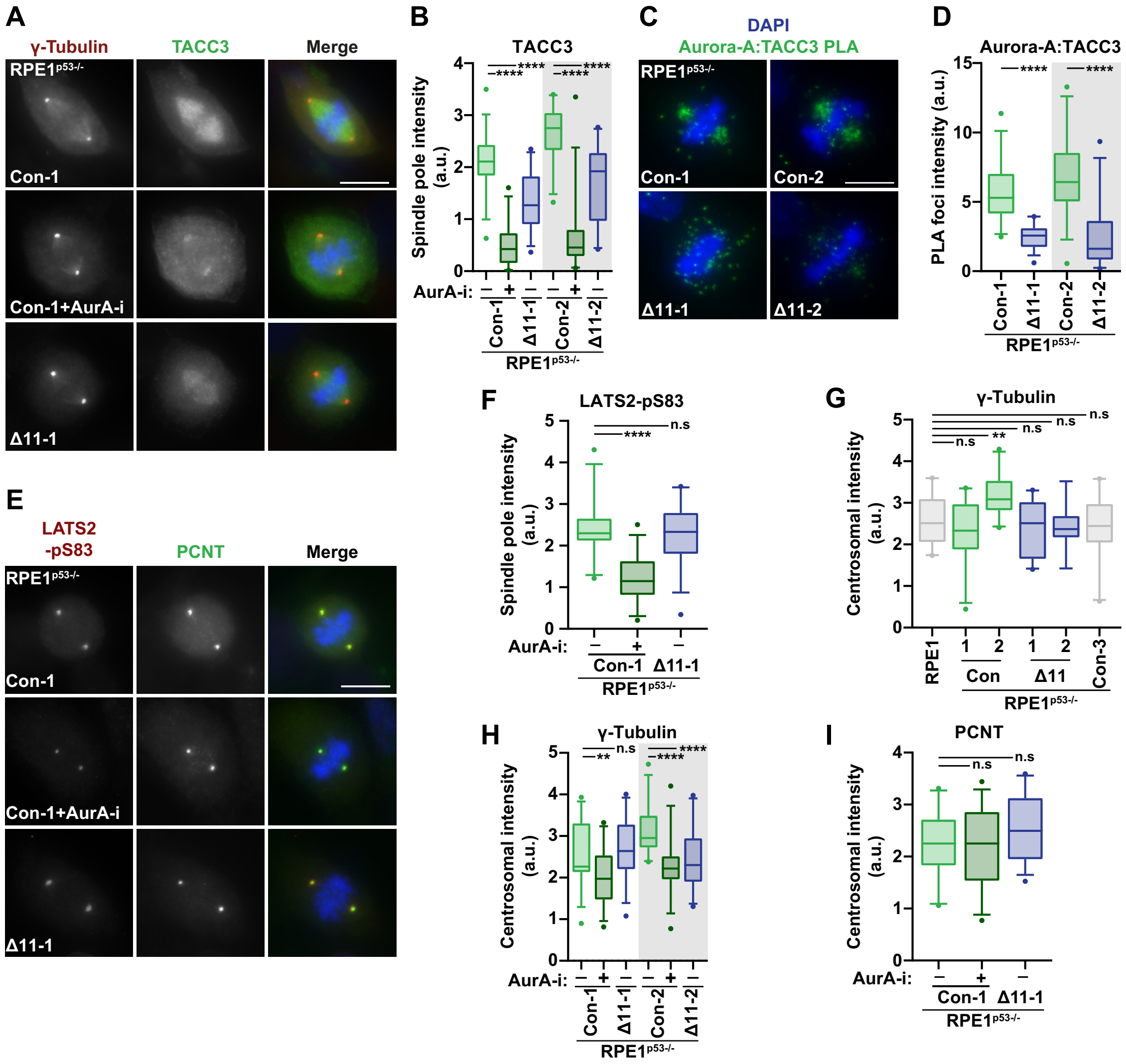

Figure EV5. Loss of the interaction between Aurora-A and CEP192 impairs TACC3 spindle recruitment.

(**A**) Immunofluorescence images of control and Δ11 RPE1$^{p53-/-}$ cells treated with either DMSO control or Aurora-A inhibitor (30 min) prior to methanol fixation. Antibodies against γ-tubulin and TACC3 are red and green in merged images, respectively, with DNA stained with DAPI (blue). (**B**) Box plot TACC3 spindle signal intensity in RPE1$^{p53-/-}$ cells, with representative images shown in (**A**) ($n = 2$, ≥15 cells/biological replicate). Exact p values (L-R): <0.0001. <0.0001, <0.0001, <0.0001. (**C**) Proximity ligation assay (PLA) between Aurora-A and TACC3 specific antibodies in control and Δ11 RPE1$^{p53-/-}$ cells. PLA signal is green in merged images with DNA stained with DAPI (blue). (**D**) Box plot of Aurora-A:TACC3 PLA signal intensity in mitotic RPE1$^{p53-/-}$ cells, with representative images shown in (**B**) ($n = 2$, ≥15 cells/biological replicate). Exact p values (L-R): <0.0001, <0.0001. (**E**) Immunofluorescence images of control and Δ11 RPE1$^{p53-/-}$ cells treated as in (**A**). Antibodies against LATS2-pS83 and PCNT are red and green in merged images, respectively, with DNA stained with DAPI (blue). (**F**) Box plot of LATS2-pS83 spindle pole signal intensity in RPE1$^{p53-/-}$ cells, with representative images shown in (**E**) ($n = 2$, ≥15 cells/biological replicate). Exact p values (L-R): <0.0001, 0.6749. (**G**) Box plots of γ-tubulin centrosomal signal intensity in multiple RPE1 and RPE1-derived cell lines ($n = 2$, 10 cells/biological replicate). RPE1 indicates the parental p53$^{+/+}$ line from which other clones were derived. Con-3 is an additional RPE1$^{p53-/-}$ control cell line generated previously within the lab. Exact p values (L-R): 0.1865, 0.0022, 0.4935, 0.3363, 0.6783. (**H**) Box plot of γ-tubulin centrosomal signal intensity in RPE1$^{p53-/-}$ cells treated as in (**A**), with representative images also shown in (**A**) ($n = 2$, ≥15 cells/biological replicate). Exact p values (L-R): 0.0017, 0.3941, <0.0001, <0.0001. (**I**) Box plot of PCNT centrosomal signal intensity in RPE1$^{p53-/-}$ cells treated as in (**A**), with representative images shown in (**E**) ($n = 2$, ≥15 cells/biological replicate). Exact p values (L-R): 0.7364, 0.0919. Data information Box plots in (**B**, **D**) and (**F**–**I**) indicate the median and interquartile ranges (25th–75th percentile) with coloured whiskers representing 5th–95th percentile ranges. Grey shading in (**B**, **D**, **H**) denotes independently completed biological replicates. p values are denoted as follows: ****$p < 0.0001$, **$p < 0.01$, n.s not significant (Mann–Whitney test). Scale bars in (**A**, **C**, **E**) represent 10 µm. Source data are available online for this figure.
