# Supplementary material for: CEP192 localises mitotic Aurora-A activity by priming its interaction with TPX2
Source: EMBO J. 2024 Sep 26;43(22):5381–420. doi: 10.1038/s44318-024-00240-z (PMC11574021; doi:10.1038/s44318-024-00240-z)
Supplement: Supplementary file 10 — EV and Appendix Figure Source Data [file 44318_2024_240_MOESM10_ESM.zip › Movies_EV4F.zip!/Movies EV4F/Movies EV4F legend.docx]

**Movies EV4F**

Widefield time lapse imaging of control and Δ11 RPE1^p53-/-^ cells progressing through mitosis into anaphase. These movies correspond to the representative images displayed in **Figure EV4F.** Left: SiR-Hoechst, right: brightfield. Time is relative to nuclear envelope breakdown and expressed as hh:mm, frames were taken at 2 minute intervals. Scale bar corresponds to 10 µm.

Files:

**Source Data_Movies EV Figure 4F_Control-2**

Movie of a representative RPE1^p53-/-^ Control-2 cell.

**Source Data_Movies EV Figure 4F_Delta 11-1**

Movie of a representative RPE1^p53-/-^ Δ11-1 cell.

**Source Data_Movies EV Figure 4F_Delta 11-1_Rotated**

Movie of a representative RPE1^p53-/-^ Δ11-1 cell that was rotated at the point of metaphase to anaphase transition.
